# Bioinformatic Analysis Reveals Conservation of Intrinsic Disorder in the Linker Sequences of Prokaryotic Dual-family Immunophilin Chaperones

**DOI:** 10.1016/j.csbj.2017.12.002

**Published:** 2017-12-30

**Authors:** Sailen Barik

**Affiliations:** 3780 Pelham Drive, Mobile, AL 36619, USA

**Keywords:** CYN, cyclophilin, CFBP, cyclosporin- and FK506-binding protein, FCBP, FK506- and cyclosporin-binding protein, DFI, dual-family immunophilin, ID, intrinsic disorder, TPR, tetratricopeptide repeat, Chaperone, Immunophilin, Intrinsic disorder, Flexible linker, Flavobacteria, Spirochetes

## Abstract

The two classical immunophilin families, found essentially in all living cells, are: cyclophilin (CYN) and FK506-binding protein (FKBP). We previously reported a novel class of immunophilins that are natural chimera of these two, which we named dual-family immunophilin (DFI). The DFIs were found in either of two conformations: CYN-linker-FKBP (CFBP) or FKBP-3TPR-CYN (FCBP). While the 3TPR domain can serve as a flexible linker between the FKBP and CYN modules in the FCBP-type DFI, the linker sequences in the CFBP-type DFIs are relatively short, diverse in sequence, and contain no discernible motif or signature. Here, I present several lines of computational evidence that, regardless of their primary structure, these CFBP linkers are intrinsically disordered. This report provides the first molecular foundation for the model that the CFBP linker acts as an unstructured, flexible loop, allowing the two flanking chaperone modules function independently while linked in *cis*, likely to assist in the folding of multisubunit client complexes.

## Highlights

• Dual-family immunophilins (DFIs) are recently discovered chimeric chaperones.
• Bacterial DFIs possess the structure CYN-linker-FKBP, abbreviated as CFBP.
• The linker sequences in CFBP are diverse but possess intrinsic disorder (ID).
• The large CFBP linker with ID is a novel discovery in prokaryotes.
• ID befits the linker's role as a flexible connector between two chaperone modules.

## Graphical Abstract

**Figure:**
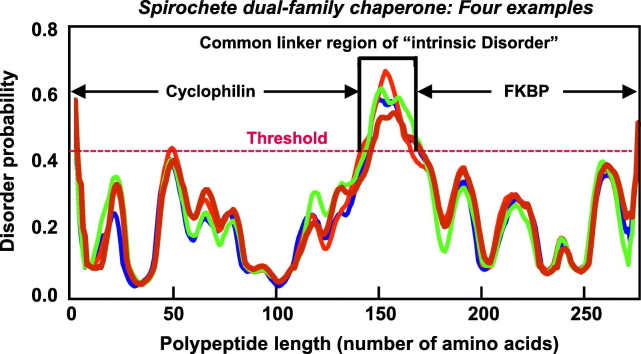

## 1 Introduction

Immunophilins of the CYN and FKBP families are ubiquitous chaperones that facilitate and regulate the folding of client proteins [1,2]. Previously, we characterized a novel class of dual-family immunophilins that are naturally occurring chimera of CYN and FKBP [1,3]. Biochemical studies of recombinant proteins showed that both the CYN and the FKBP modules in the DFIs were functional, possessing protein prolyl isomerase (PPIase) and chaperone activities [3]. We also showed that the DFI chaperones occur either as CYN-linker-FKBP or as FKBP-linker-CYN, which were given the distinguishing acronyms of CFBP and FCBP, respectively, to indicate the domain order [4]. Our recent survey showed that the DFIs are found in select microbes that are primarily extremophiles and aquatic, and suggested a model in which the double-pronged chaperoning role of a DFI is essential in simultaneous folding of multisubunit protein complexes, especially in rapidly denaturing, high-stress environments [5]. Since both CYN and FKBP domains are relatively well-known I have focused attention on the linker sequence connecting them, which is responsible for the creation of the chimera. I reasoned that the linkers hold the key to the properties and features of the DFIs that make them unique and distinct from the individual CYN and FKBP chaperones, separate homologs of which also occur in the same organisms.

In the previous report [5], I conducted a detailed study of the linkers of the FCBP-type DFIs, and showed that they consist of three tandem tetratricopeptide repeat (TPR) motifs (3TPR), likely from an evolutionary fusion of a ‘large’ FKBP with C-terminal TPR with a CYN. TPR motifs are approximately 34 amino acids long (hence their name) that are not conserved in sequence but contain amino acids of similar properties in strategic locations [6]. Structural studies have shown that each TPR unit is made of alpha-helices, connected by short peptides [7], such that a multi-TPR sequence can act as a spring-like linker [8]. Since FCBPs contain a 3TPR linker, these results formed the basis of our conjecture that DFI linkers allow flexible movement of the FKBP and CYN domains to accommodate multisubunit proteins of diverse sizes for fast and simultaneous chaperoning of two or more subunits. However, the linkers in the CFBP-type DFIs, which are diverse and featureless short sequences, averaging only about 51 amino acids in length [5], have not been characterized or analyzed further. The current study presents a comprehensive analysis of linkers in 277 CFBP sequences, mined from GenBank, and documents that they possess the hallmarks of “intrinsic disorder” (ID), a recently appreciated feature of protein sequences that are natively unstructured and often constitute regions of intramolecular flexibility [[9], [10], [11], [12], [13], [14]].

## 2 Materials and Methods

### 2.1 Sequence Retrieval, Comparison and Phylogenetic Analysis

All CFBP sequences were retrieved from NCBI GenBank as described before [5]. Various known CYN and FKBP sequences of human, *Drosophila*, yeast, *A. thaliana* and from known FCBP and CFBP [3] were joined in silico in the order CYN-FKBP and used as query in BLAST search of NCBI protein databank. Only those hits that contained both CYN and FKBP in the same polypeptide were selected by visual inspection, as most CFBP organisms possessed individual CYN and FKBP genes that were also returned by the search. Multiple and exhaustive iterations were performed, which retrieved new sequences, until no new sequences were found.

Multiple sequence alignments were performed by Clustal Omega [15] at the EMBL-EBI web server [16], as described [5]. The output, saved in Newick format, was drawn using Dendroscope 3 (http://www.dendroscope.org), an open source and interactive software for phylogenetic display [18], whereby the rectangular Cladogram format was preferred, as many CFBP organisms are highly related but placed in separate clades.

### 2.2 Disorder Analysis

Since 2010, several dozen programs have been developed for prediction of intrinsic disorder (ID) in protein sequences, highlighting the flurry of research in this area [19,[20], [21], [22]]. As disorder prediction is the major focus in this paper, a brief description of the technique is in order. Fundamentally, all predictors are based on the premise that disordered regions should have a higher frequency of hydrophilic and charged residues, and lower sequence complexity. Technically, the current predictors rely on physicochemical properties or machine learning classifiers, or a combination [17,[20], [21], [22], [23]]. Several methods use a meta-approach that combines predictions from multiple predictors, but this often results in slow computing. Here, I have chosen PrDOS [24] because it is relatively fast, offers a simple graphical user interface, allows batch analysis of up to 50 sequences, and is a hybrid that uses both template-based and machine-based predictions. It is also relatively unbiased, without favoring and disfavoring any features or motifs such as disulfide bonds or metal-binding regions [17]. For each sequence, a scoring matrix is generated after two-rounds of PSI-BLAST search of sequence databases. The profiles are then used for a template-based search for a homolog with known disorder status in the PDB. For sequences that do not have a homolog, a support vector machine (SVM) algorithm is used to obtain the position-specific scoring matrix. PrDOS allows interactive user-selected false-positive rates (FPR) that range from 1 to 25%. The FPR determines the threshold, above which the disordered prediction is increasingly more reliable. After optimizations of the FPR with several known ID sequences (e.g. in p53) [11] and 3D structures (e.g. CYN and FKBP proteins), and thereby ascertaining that PrDOS correctly showed both the presence and absence of ID regions, an FPR of 8% was selected as the most optimal for routine analysis, which translated into a “disorder probability” (DP) threshold of 0.43. In analyzing a CFBP, this threshold was used as the baseline. Nevertheless, to rule out any computational bias, we subjected a single CFBP sequence to disorder prediction by multiple programs, and the PrDOS results agreed with essentially all of them, including several meta-predictors (data not shown), such as MetaDisorder, which compares nearly two dozen different methods [25], and PONDR-FIT, which combines five [26]. For routine analysis, the PrDOS results were downloaded as CSV (comma-separated values) files, and then imported into Excel for further analysis and graphing.

For ab initio structure prediction, the Rosetta software [27] was used in the Robetta server [28].

### 2.3 Analysis of Amino Acid Enrichment

Amino acid composition of the linkers was determined by the use of Composition Profiler (www.cprofiler.org), a web-based tool that automates detection of enrichment or depletion patterns of amino acids classified by user-chosen properties [29]. For the linker analysis, “disorder propensity” was chosen and all 277 CFBP linkers were collectively analyzed as “query sample”, and the rest of the CFBP sequence (i.e., CYN + FKBP) as “background sample”.

## 3 Results

### 3.1 CFBP Sequences and the Organisms

In the last survey [5], I reported a total of 84 CFBP sequence, in which two kinds of bacteria, namely Flavobacteria and Spirochetes, contributed essentially all the sequences. Since then, I have continued to collect more CFBP sequences from GenBank, sometimes using and reusing the newly mined sequences as query in various BLAST formats. In such cases, the new sequence fetched its closest relatives in the same subclade, which did not appear previously due to their weaker homology with the distant query and the stringency of the search. In my most updated and current list, reported here (Supplementary Material 1), the CFBPs number at 277. In the shorter previous list, I made the observation that all CFBP organisms were bacterial in nature. In the current CFBP roster (Supplementary Material 2), Flavobacteria and Spirochetes still dominate, but now two eukaryotic organisms were also noted, namely *Ancylostoma ceylanicum*, which is a nematode (hookworm in man and other mammals), and *Hyalella azteca*, an arthropod (a small shrimp). However, the predicted polypeptides of both sequences were noted to have some unusual or abnormal features (Supplementary Material 1). The *A. ceylanicum* polypeptide has a unique extension of DATVQKDDHHGHDHSDPNHKH at the C-terminus; when BLAST search was performed with this peptide as query, it retrieved this same entry as expected, but unexpectedly, also found significant homology with P-type ATPase fragments from several bacteria, including Bacteroidetes and Flavobacteria. In addition, it retrieved a second CFBP (NCBI accession number WP_010521440.1) from *Aquimarina agarivorans* (i.e., besides the one in the Supplementary Material 1, Supplementary Material 2), a Flavobacterium, which had a similar extension at the C-terminus (KDNH---DHSDPNHKH). It is to be noted that in their natural environment, the larvae of hookworm, including several species of the *Ancylostoma* genus, feed on soil bacteria and molt twice before they become third-stage larvae [30]. It is tempting to speculate that the bacterial feeding results in occasional genetic recombination between *Ancylostoma* and bacteria, which can be an area of interesting future study. The *H. azteca* polypeptide is missing ~60 amino acids at the N-terminus, including the structurally important “N-terminal loop” of CYN (residue 19–24 in hCyPA) [31] and multiple highly conserved peptide sequences, such as FHR. Neither the *A. ceylanicum* nor the *H. azteca* protein has been biochemically characterized. Thus, both sequences may need to be curated and validated further, and barring these putative candidates, CFBP remains a prokaryotic enzyme.

### 3.2 Identification of the Linker Region in CFBP

**Fig. 1:**
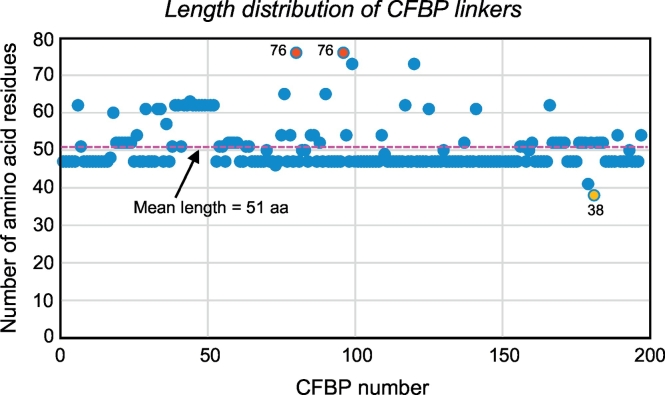
Length distribution of CFBP linkers. The number of amino acid residues in all 277 CFBP linkers was manually counted and tabulated in Excel (not shown). In plotting them in graph, however, the over-crowding was minimized by removing sequences that were in the same phylogenetic clade (from Clustal Omega ordering) and nearly identical to each other (>90% identical), while including many representative ones (Supplementary Material 1). For instance, *Treponema putidum* CFBP was removed because it was 94% identical to *Treponema denticola* CFBP. However, the mean length (51 amino acids, pink dotted line in color) was in fact calculated from the full roster of 277 sequences; the shortest (38 amino acids, orange in color) and two longest sequences (76 amino acids, red in color) were also not removed, as they were evidently different from all others. The final graph, shown here, represents 197 sequences. (For interpretation of the references to color in this figure legend, the reader is referred to the web version of this article.) Length distribution of CFBP linkers. The number of amino acid residues in all 277 CFBP linkers was manually counted and tabulated in Excel (not shown). In plotting them in graph, however, the over-crowding was minimized by removing sequences that were in the same phylogenetic clade (from Clustal Omega ordering) and nearly identical to each other (>90% identical), while including many representative ones (Supplementary Material 1). For instance, *Treponema putidum* CFBP was removed because it was 94% identical to *Treponema denticola* CFBP. However, the mean length (51 amino acids, pink dotted line in color) was in fact calculated from the full roster of 277 sequences; the shortest (38 amino acids, orange in color) and two longest sequences (76 amino acids, red in color) were also not removed, as they were evidently different from all others. The final graph, shown here, represents 197 sequences.

Demarcation of the boundaries of the linker between CYN and FKBP essentially involved determining where the CYN sequence ends and the FKBP sequence begins. This was performed in several steps. First, full-length CFBPs as well as their various parts were BLAST-searched against the GenBank protein database, and also aligned against various known CYN and FKBPs. During these procedures, the goal was to assign the maximal amount of CFBP sequence to CYN and FKBP domains, so that the leftover sequence between them is purely the linker, not contaminated by CYN C-terminal or FKBP N-terminal residues. The CYN and FKBP sequences were also recognized by their respective conserved signature peptides and invariant residues, appreciated by multiple alignment, such as FHR, MAN, W, and H in CYN [31] and HY and FDSS in FKBP [19] (highlighted in the first sequence in Supplementary Material 1). Finally, multiple alignment of all CFBP sequences confirmed the CYN and FKBP regions because of their high degree of similarity among all sequences, whereas the linker region between them was diverse (see Phylogeny below). Once fully identified (colored blue in Supplementary Material 1), the 277 linkers were further analyzed for their sequence features. Their mean length was 51 amino acids, ranging from 38 in *Treponema saccharophilum* to 76 in *Flavobacterium hydatis* and *Flavobacterium succinicans* (Fig. 1). Intriguingly, the most common length of the linker appeared to be 47 amino acids, the significance of which is unknown, as the linker length did not follow phylogeny.

### 3.3 Phylogeny of CFBP Linkers

**Fig. 2:**
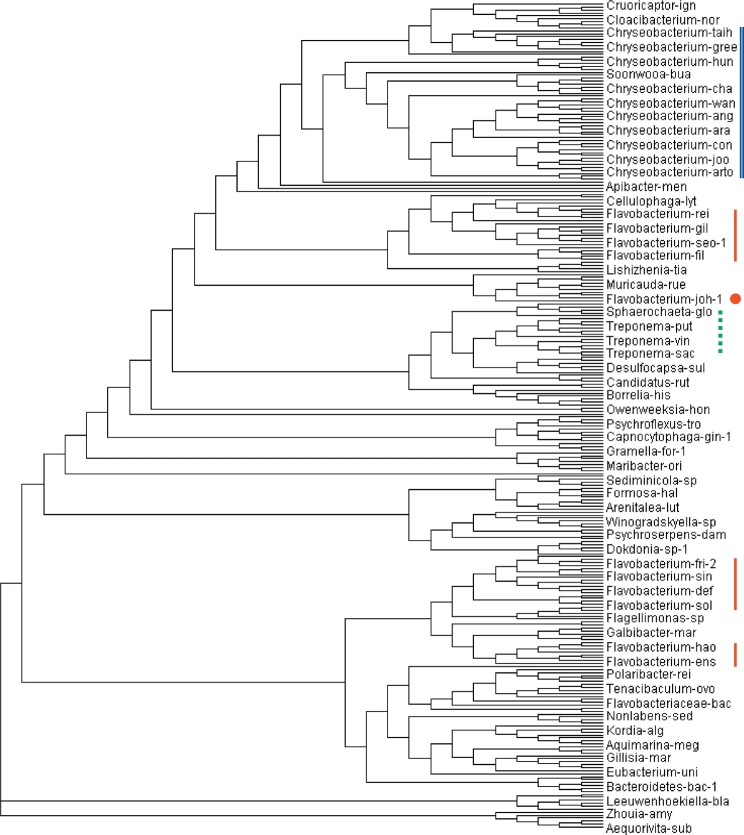
Phylogenetic relationship among the CFBP linkers. The cladogram tree was generated with 277 linker sequences as described in Materials and Methods. For space constraints, only the major nodes and clade-representative names are shown but this should not detract from the conclusions described in the Results. The scattered *Flavobacteria* branches are indicated by solid lines and a dot (red in color), the *Chrysobacterium* clade by double-line (blue in color), and *Treponema*/*Sphaerochaeta* (*Spirochaeta*) clades by dotted line (green in color). Note that another spirochete clade, belonging to *Borrelia*, is also close by (unmarked).

In the preceding study [5], comparison of full-length CFBP sequences by multiple alignment showed an overall close relationship among all, although each type of organism showed clustering; for example, all Flavobacteria were clustered together, and so were all Spirochetes. Addition of the newly discovered CFBP sequences reported here did not alter the pattern (data not shown). However, to what extent the relationship was contributed by the CYN and FKBP sequences or by the linkers was not known. To specifically focus on the evolutionary relationship among the CFBP linkers, the linker sequences identified above (Supplementary Material 1) were subjected to similar multiple alignment and found a generally similar pattern with minor changes. For example, Chrysobacterial linkers were clustered together, while the Flavobacterial linkers were spread over three rooted clusters as shown (Fig. 2). The linker of *Soonwooa buanensis*, a marine bacterium of the *Flavobacteriaceae* family, recently discovered in the Yellow Sea off the Korean coast, was placed in the Chrysobacterial group (Fig. 2), but this is not an aberration, since 16S RNA sequencing revealed that *S. buanensis* is most closely related to several *Chrysobacterium* species (94.4% similarity in 16S RNA sequence) [32]. Thus, although named in memory of Soon-Woo Hong, a Korean microbiologist, this bacterium is essentially a *Chrysobacterium* sp. Following the same pattern, the *Spirochaete* (e.g. *Treponema* and *Sphaerochaeta*) linkers are also clustered together, and separated from the *Flavobacteria* (Fig. 2). We conclude that the CFBP linker sequences generally follow the similarities among their host bacterial species, which may suggest their evolution through genetic exchange between phylogenetically close strains rather than large-scale horizontal transfer across distant genera. Importantly, no single common sequence motif could be found among all the linkers (Supplementary Material 1).

### 3.4 Predicted Disorder in the CFBP Linker

**Fig. 3:**
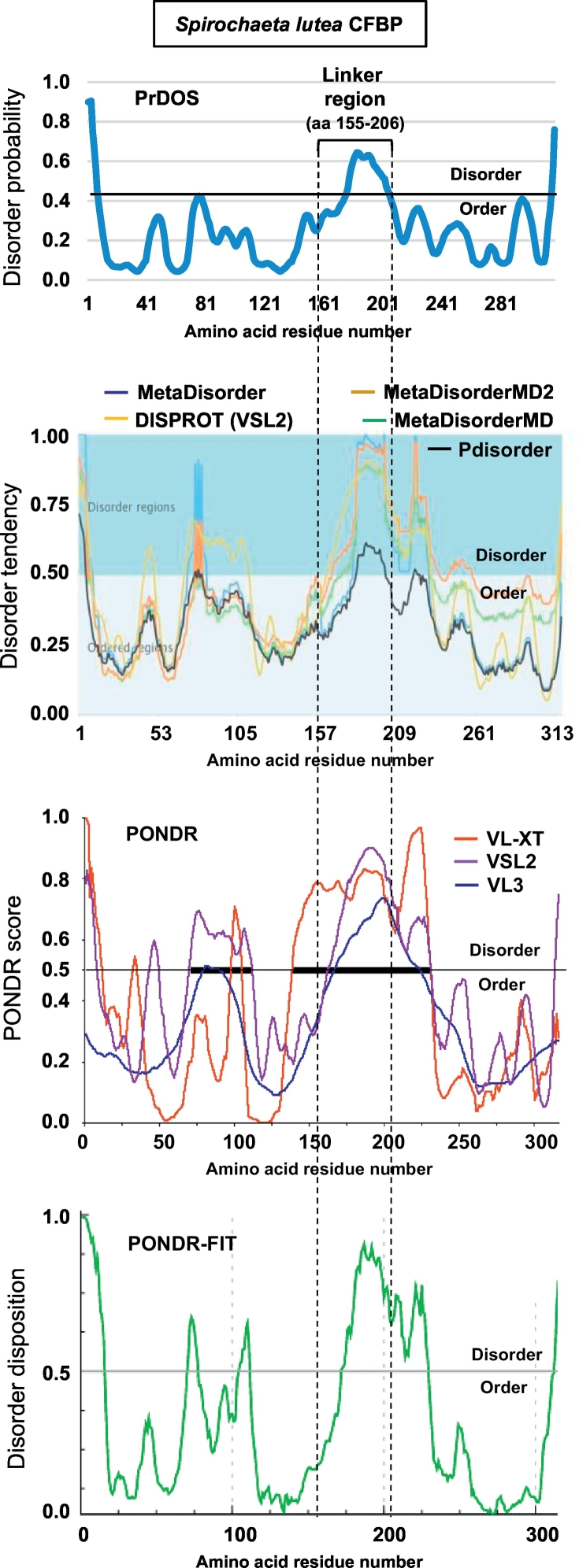
Comparison of multiple disorder prediction programs and validation of PrDOS. The *Spirochaeta lutea* CFBP (with a 52-amino acid linker, close to the average linker size in all CFBPs, see Fig. 1), was arbitrarily chosen for disorder prediction by multiple programs that are freely available, as described in Materials and Methods [17, 18]. The PrDOS result (used in the rest of the paper) is compared with the major programs as shown. The default order/disorder cut-off for each program is indicated, and 8% FPR (false positive rate) was chosen for PrDOS, which was translated into 0.43 cut-off, as explained in Materials and Methods. The default terminology and scale of the respective programs were also used to describe their Y-axes. The dotted lines running through all plots bracket the linker sequence (amino acid 155–206) and show the agreement among the programs. Note that in this particular CFBP, the disorder happens to be slanted towards the C-terminal half of the linker. The high spike of disorder in the two termini of the CFBP is a general feature of most proteins, and is to be ignored for this study.

Since the linker regions of CFBP, unlike the CYN and FKBP domains, had no consensus motif or recognizable protein family domain, a logical query was whether they are in fact unstructured; in other words, whether intrinsic disorder (ID) is a commonality among them. As mentioned earlier, lack of a fixed structure, by which ID is defined, has been found to allow flexibility of polypeptides, which would ideally suit the function of the linker connecting CYN and FKBP. To test this, and at the same time locate the disordered region without bias, the full length of each CFBP sequence was subjected to intrinsic disorder prediction. We first compared multiple web-based tools [17] to rule out any programmatic bias as described in Materials and Methods. A single, arbitrary CFBP, namely that of *Spirochaeta lutea*, was chosen and default parameters of each program was used. A total of 15 programs were tested, results from 10 of which are shown (Fig. 3), all of which identified the linker as the region of highest consistent disorder. Results of SPINE-D, Poodle, RONN, and Spritz [17], which use very similar algorithms, were essentially identical, and therefore, not presented to avoid crowding. Having shown that the output of all major programs matched with the PrDOS prediction, we used PrDOS [24] for the rest of our studies, as elaborated in Materials and Methods.

**Fig. 4:**
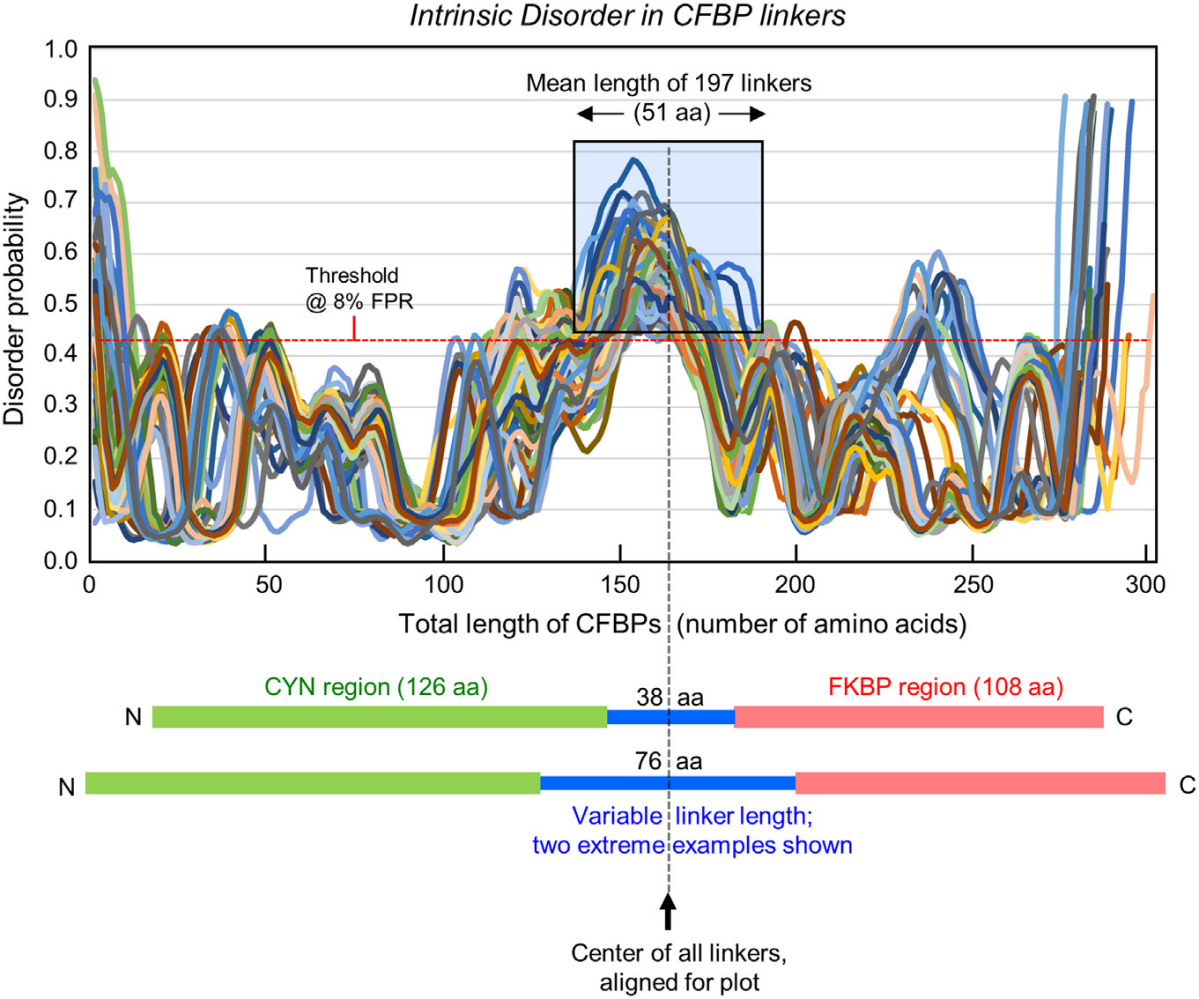
Intrinsic disorder is a common feature in all CFBP linkers. Analysis and plot of the disorder probability have been described in detail under Materials and Methods. Each CFBP graph is color-coded by the default color pattern of Excel. Amino acid number is on X-axis, and disorder probability, on Y-axis. The range of linker sequence length in the population (38 and 76 amino acids, and shortest and the longest, respectively) is illustrated schematically at the bottom (Blue in color view), along with the constant lengths of the conserved CYN (Green in color) and FKBP (Red in color) regions, chosen for this analysis. The baseline (threshold) and the FPR (False Positive Rate) have been described in Materials and Methods. Within the premise of this paper, the abnormally high disorder probabilities at the very termini of all CFBP are to be ignored, as terminal disorder is a general feature of all proteins, commonly found in X-ray crystallography [26].

Localization of disorder to the linker region then required a superimposed plot of the disorder probability of all CFBP sequences. However, as the CFBPs and the linkers had diverse lengths, a straightforward superimposition of all plots would create a large and fuzzy mix, difficult to interpret. To circumvent this problem, PrDOS analysis was conducted with a shorter version of the CFBPs, in which the relatively diverse terminal regions of CFBPs were removed, saving the conserved core regions of CYN and FKBP (126 and 108 amino acids, respectively) as reference points (Fig. 4) and the full-length linker in between. Subsequently, the probability plots were all aligned along the center of the linkers. The resulting graph (Fig. 4) clearly revealed that essentially the full lengths of all linkers were disordered, with probability significantly above the threshold of 0.43 (see Materials and Methods).

**Fig. 5:**
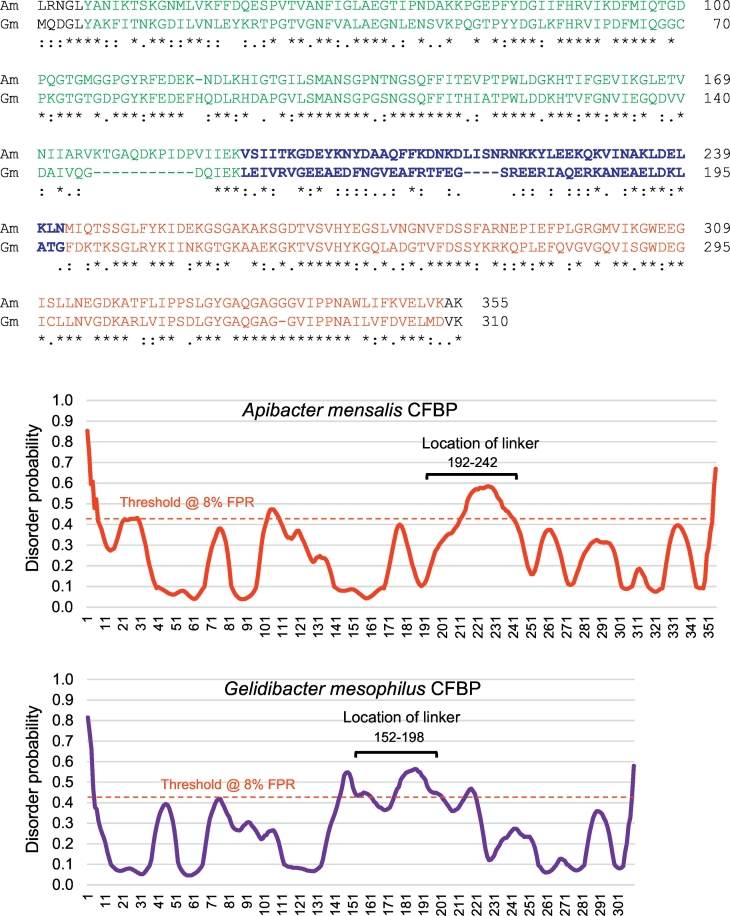
Intrinsic disorder in linkers of distant homology. Top: Primary sequences of the *Apibacter mensalis* (Am) and *Gelidibacter mesophilus* (Gm) CFBPs were aligned by Clustal Omega, and the three regions (CYN, loop, FKBP) are indicated by color (Teal, Dark Blue, and Red, respectively); the linker is additionally in bold. Asterisks and dots respectively indicate identical and conservatively replaced amino acids. Note the significantly lower amino acid identity in the linker region, but higher conservative replacements, contrasting with the high amino acid identity in the CYN and FKBP regions. Bottom: Disorder graph for the two single CFBPs as named, plotted as in Fig. 4. Note that the highest regions of disorder coincide with the respective linker positions.

To further confirm that the linkers are disordered regardless of their primary structure, two CFBPs that were highly separated in sequence phylogeny (see Fig. 2) were chosen for disorder comparison; they were CFBPs of *Apibacter mensalis* and *Gelidibacter mesophilus*. Alignment of the two sequences (Fig. 5 top) confirmed that they are indeed most dissimilar in the linker region, while the ‘control’ CYN and FKBP regions are well conserved. Since the individual graphs were difficult to distinguish in the joint plot, these two disorder plots were examined separately (Fig. 5 bottom), which clearly showed that both linkers were disordered. These results document that disorder is indeed a common structural feature of the CFBP linkers regardless of the diversity in their amino acid sequences.

As described below, the linker disorder prediction was further supported by the correct prediction of the unstructured regions in the ‘control’ CYN and FKBP sequences and by the Rosetta prediction program.

### 3.5 Intrinsic Nature and Portability of the Linker Disorder

**Fig. 6:**
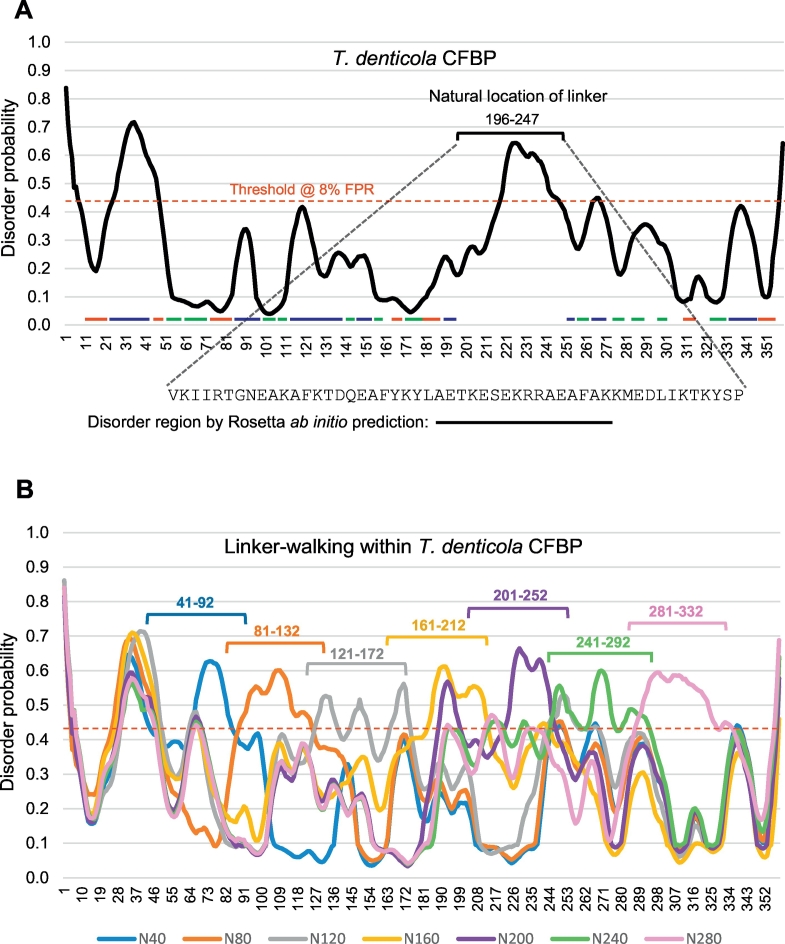
Disorder plot for *Treponema denticola* CFBP with relocated linker sequences. Disorder analysis and plot were performed essentially as in the two preceding Figures (Fig. 4, Fig. 5). (A) Top: Plot for native *T. denticola* CFBP with the 52-amino acid long linker region in its natural location. In addition, secondary structural elements, corresponding to known structures of homologous CYN (bovine CyP40, PDB 1iip) and FKBP (*Arabidopsis thaliana* FKBP42, PDB 2if4) sequences, are so marked (in color: Green = β-strand; Red = α-helix; Blue = unstructured coiled-coil) (Supplementary Material 3). Bottom: Rosetta ab initio prediction of disordered region is shown below, along with the corresponding amino acid sequence, which is also located within the linker. (B) Plots for seven in silico constructs with the same linker region positioned every 40 amino acids. The constructs are code-named by linker location, e.g., N40 means that the linker starts after 40 amino acids from the N-terminus (residue 41 to 92). (For interpretation of the references to color in this figure legend, the reader is referred to the web version of this article.) Disorder plot for *Treponema denticola* CFBP with relocated linker sequences. Disorder analysis and plot were performed essentially as in the two preceding Figures (Fig. 4, Fig. 5). (A) Top: Plot for native *T. denticola* CFBP with the 52-amino acid long linker region in its natural location. In addition, secondary structural elements, corresponding to known structures of homologous CYN (bovine CyP40, PDB 1iip) and FKBP (*Arabidopsis thaliana* FKBP42, PDB 2if4) sequences, are so marked (in color: Green = β-strand; Red = α-helix; Blue = unstructured coiled-coil) (Supplementary Material 3). Bottom: Rosetta ab initio prediction of disordered region is shown below, along with the corresponding amino acid sequence, which is also located within the linker. (B) Plots for seven in silico constructs with the same linker region positioned every 40 amino acids. The constructs are code-named by linker location, e.g., N40 means that the linker starts after 40 amino acids from the N-terminus (residue 41 to 92).

Absence or presence of structure is an intrinsic property of the amino acid sequence, although the structure can be modestly influenced by allostery [33,34]. In particular, for an unstructured or disorder region to act as a linker as proposed, it should ideally function as an independent module, unaffected by the neighboring sequences. To test that disorder of the CFBP linker is unaffected by the flanking CYN or FKBP sequences, we moved the linker sequence to various locations in the CFBP sequence in silico and subjected each virtual construct to PrDOS analysis. This analysis was performed on multiple, arbitrarily chosen CFBPs, representing diverse sequences from different clades, but only the results of the *Treponema denticola* CFBP (TdCFBP) were presented due to space constraints. On each CFBP, the linker was moved every 20 nucleotides, although only the 80-residue movement data have been presented to save space. These results (Fig. 6) make several important observations. First of all, the single isolated TdCFBP disorder prediction plot allowed a clearer structural view of the CFBP that can be authenticated by independent means, which was not possible with the large number of superimposed plots in Fig. 4. Experimentally determined tertiary structures are available for several CYN and FKBP sequences, which has revealed overall conservation of the tertiary structure for each class. By BLAST homology search with TdCFBP as query, one CYN and one FKBP sequence were retrieved that were most similar to TdCFBP and for which the X-ray crystal structures were available (Supplementary Material 3). The secondary structure elements were then marked on the TdCFBP amino acid sequence as shown (Fig. 6A). It was indeed found that the structural elements fully complemented the disorder plot, i.e., the areas of α-helix and β-strand scored lower in disorder, whereas the coiled-coil areas scored higher. It is to be noted that the coiled-coil elements in proteins can also be relatively flexible and unstructured, and believed to act as molecular rulers between flanking structures [35]. Thus, it is natural that PrDOS assigned them a generally higher disorder probability than the neighboring helix and strand areas. Nonetheless, the linker region in TdCFBP is much larger (52 amino acids) than the longest coiled-coil (27 amino acids) segment, and also had a pronouncedly higher disorder probability (Fig. 6A), consistent with its distinctive role as the inter-chaperone linker. Overall, the known structural elements in the CYN and FKBP segments served as internal controls, validating the PrDOS prediction and adding confidence to the finding that the CFBP linker region is indeed highly disordered. However, homology modeling could not be used to validate the disorder in the linker, since the linker sequence is uniquely found in dual-family immunophilins, and has no structural homolog. Thus, a homology-independent, ab initio folding model, namely Rosetta [27], was employed specifically for this region, which indeed confirmed disorder in the linker (Fig. 6A, bottom). Considering that PrDOS and Rosetta use different algorithms and cut-offs for folding prediction, this agreement is remarkable and offers unbiased cross-validation.

**Fig. 7:**
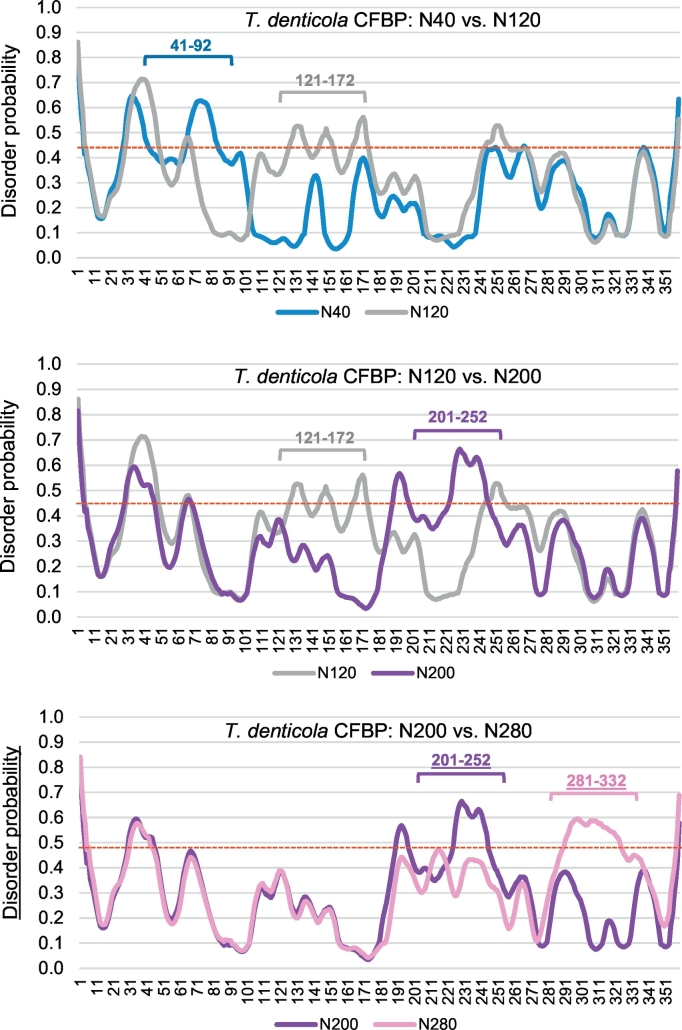
Pairwise disorder plots for *T. denticola* CFBP with relocated linker sequence. The analysis and graphing were performed as in Fig. 6. These are the same plots as in Fig. 6, but presented pairwise for easy viewing and comparison; they also share the same color code for the graphs.

Once the authenticity of PrDOS prediction was confirmed and the disordered nature of the linker in its native location in TdCFBP was fully ascertained, the linker-walking analysis was ready to be undertaken. As stated above, the PrDOS results are presented for the linker region, conceptually translocated every 40 residues, at distances of 40, 80, 120, 160, 200, 240 and 280 residues from the N-terminus (Fig. 6B). For the full perspective, note that the natural location of the TdCFBP linker starts at 196 residues from the N-terminus. As seen (Fig. 6B), the highest peak of disorder in every construct coincides with the linker region in the construct. However, while the superimposed presentation of the seven plots helped to visualize the movement of the disordered region from one construct to the next, it made viewing of the individual plots difficult. Thus, in a different series of graphs (Fig. 7), disorder of each construct and its next neighbor were presented, although for space reasons, only three pairs are shown, namely N40-N120, N120-N200, and N200-N280. A close examination of these graphs clearly reveals that the linker region disorder moves with linker, from one location to the next, whereas the patterns of all other regions (belonging to CYN or FKBP) remain stationary, in their respective native locations. Taken together, these results (Fig. 4, Fig. 5, Fig. 6, Fig. 7) establish that disorder is associable and portable with the linker, and is, therefore, an intrinsic property of the linker sequence.

### 3.6 Amino Acid Composition of the CFBP Linkers

**Fig. 8:**
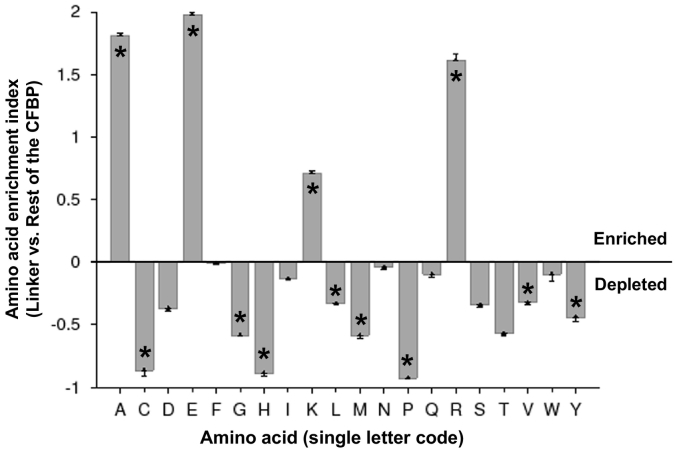
Amino acid composition bias in the CFBP linker. The computation of the enrichment index by Composition Profiler has been described in Materials and Methods. Amino acid names are in standard single-letter codes; significant enrichment and depletion of residues, constituting a characteristic flavor of disorder [17], are indicated with asterisks.

Areas of internal disorder (ID) also exhibit a characteristic amino acid compositional bias [11]. Specifically, ID regions tend to have a higher content of polar and small amino acids (Asp, Glu, Arg, Lys, Gln, Ala) and lower content of bulky hydrophobic and aromatic amino acids (Cys, Gly, Ile, Leu, Val, Met, Phe, Pro, Trp, Tyr, His) [11,17,36]. This allows the ID region to be unstructured and solvent-exposed for dynamic linker function and/or interaction with other proteins. To see if the CFBP linkers exhibit this amino acid composition of IDs, the linker sequences were collectively analyzed by the Composition Profiler toolset as described in Materials and Methods. The results (Fig. 8) indeed show a preponderance of Ala, Glu, Lys and Arg, and scarcity of Gly, His, Leu, Met, Pro, Trp, Val and Tyr in the CFBP linker sequences, authenticating their disordered nature.

## 4 Discussion

In this paper, multiple lines of evidence are presented for intrinsic disorder (ID) in the linker region of dual-family immunophilins (DFI) of the CFBP (CYN-linker-FKBP) type. This study complements the previous study showing that dual-family chaperones of the other kind, i.e., the FCBPs, in which the order of the two chaperones is reversed, contain three TPRs in their linker region. As mentioned before, TPR domains have been noted for their spring-like flexibility, promoting folding as needed [8]. Thus, in both types of DFI, the linker regions have hallmarks of flexibility, important for their postulated role in facilitating a coordinated movement of the two flanking chaperone domains, assisting in the postulated dual-chaperone function [3,5].

Many ID regions have been reported in vivo, a large number being found in proteins important in signal transduction and transcription [11,13]. Although some ID regions have been associated with specific roles, such as ligand-binding, transcriptional regulation and post-translation modification, and also contain cognate sequence motifs [11], the CFBP linkers contain no discernible motif or repeat (Supplementary Material 1), which adds significance to the discovery of ID as the common feature in all of them. There is another aspect of this discovery that appears to be novel and deserves mention. Survey has shown that the average fraction of decent-size ID (>30 residues) in a protein is ~33% by length in eukaryotes, but only ~4% in prokaryotes [13,37]. Furthermore, ID regions in eukaryotic nuclear proteins have been estimated to be 42% of the length of the proteins, whereas in the proteins of the mitochondria, the symbiotic organelle of prokaryotic origin, this fraction is only ~10% [13]. These findings have led to the speculation that prokaryotes may not contain proteins with bona fide ID [13,37] and that IDs only appeared with the evolution of eukaryotes from prokaryotes. In CFBPs, however, the linker constitutes nearly 11% of the length of the CFBP (51-residue linker in CFBP length of ~475 amino acids), which is intermediate between prokaryotes and eukaryotes. The discovery of ID in the CFBP linkers may reopen the prokaryote versus eukaryote debate, while supporting the previous conjecture [5] that prokaryotes encoding the dual-immunophilin chaperones are phylogenetically more advanced and sophisticated than simple bacteria (such as *E. coli*), thus approaching the eukaryotes to some extent [38]. Of the three major kingdoms of life, the *Archaea* show the lowest ID content, constituting <1% of protein length [37], which is also consistent with our finding that *Archaea* do not contain CFBPs. Lastly, the uniqueness of the linker may offer an opportunity to design specific small molecule inhibitors by probing the disordered structure for the proper conformer that will allow stable binding [39]; such drugs may act as potent antibiotics against the CFBP bacteria, many of which are pathogenic to diverse hosts [5].

The origin and evolution of the CFBP linkers continue to be a mystery, particularly because they are not found in any bacteria (or any organism) outside the CFBP family. Nevertheless, sequence comparison within the CFBP family (Fig. 2) suggested that CFBP genes may have been laterally transferred between genetically close bacteria, with evolutionary mutations in the linker to best fit the needs of each species or strain. The discovery of internal disorder as the only identifiable feature in the linkers – and likely important for function – allowed the primary sequence the freedom to evolve so long as the disorder was maintained. Evidently, this could be achieved by conservative replacement with non-identical amino acids, which is indeed found to be the case. In comparing two phylogenetically distant linkers that share very few identical amino acids, for example, those of *A. mensalis* and *G. mesophilus*, the large number of conservatively replaced amino acids is easily noticeable (Fig. 5), which also fit the compositional criteria for disordered sequences (Fig. 8). This is in sharp contrast to the CYN and FKBP regions, in which most amino acids are identical rather than conservative (Fig. 4), likely due to the need of identical amino acid side chains for PPIase function. In summary, intrinsic disorder in the CFBP linker may offer three major biological advantages: conformational flexibility to serve as a linker between two disparate chaperones, modular portability in genetic recombination, and resistance to loss of function because of its ability to draw on a broad repertoire of conservative replacements that can also allow finer mutational selection at the same time.

The following are the supplementary data related to this article.

Supplementary Material 1 All collected CFBP sequences are presented in FASTA format. Font colors are: Green = CYN part, Blue = the linker (which is intrinsically disordered, as shown in this paper), Red = FKBP. A few invariant residues and peptides are highlighted in the first sequence, which served as signature in recognizing the CYN and FKBP sequences and their limits. Sequences that are highlighted in yellow are nearly redundant in sequence to several others of the same bacterial genus, and were not used in Fig. 1 to reduce crowding; however, all sequences were included in all analyses and conclusions. Supplementary Material 1

Supplementary Material 2 List of all CFBP organisms.
In the “Others” category, two multicellular organisms are highlighted (nematode, shrimp).
The nematode (hookworm in man and other mammals) has a unique C-terminus and short N-terminus.
In the arthropod (small shrimp), the CYN part is missing ~60 aa, including the “N-terminal loop” (19–24 in hCyPA).
GAL66646.1 was not used because of its short N-terminus. Supplementary Material 2

Supplementary Material 3 Secondary structure map of *Treponema denticola* CFBP, based on homology modeling against bovine CyP40 (PDB 1iip) [1] and *Arabidopsis thaliana* FKBP42 (2if4) [2]. Supplementary Material 3
