# Supplementary material for: Bioinformatic Analysis Reveals Conservation of Intrinsic Disorder in the Linker Sequences of Prokaryotic Dual-family Immunophilin Chaperones
Source: Comput Struct Biotechnol J. 2017 Dec 30;16:6–14. doi: 10.1016/j.csbj.2017.12.002 (PMC5852385; doi:10.1016/j.csbj.2017.12.002)
Supplement: Supplementary Material 1 — All collected CFBP sequences are presented in FASTA format. Font colors are: Green = CYN part, Blue = the linker (which is intrinsically disordered, as shown in this paper), Red = FKBP. A few invariant residues and peptides are highlighted in the first sequence, which served as signature in recognizing the CYN and FKBP sequences and their limits. Sequences that are highlighted in yellow are nearly redundant in sequence to several others of the same bacterial genus, and were not used in Fig. 1 to reduce crowding; however, all sequences were included in all analyses and conclusions. [file mmc1.docx]

**Supplementary Material 1**

All collected CFBP sequences are presented in FASTA format. Font colors are: Green = CYN part, Blue = the linker (which is intrinsically disordered, as shown in this paper), Red = FKBP. A few invariant residues and peptides are highlighted in the first sequence, which served as signature in recognizing the CYN and FKBP sequences and their limits. Sequences that are highlighted in yellow are nearly redundant in sequence to several others of the same bacterial genus, and were not used in Fig. 1 to reduce crowding; however, all sequences were included in all analyses and conclusions.

>Aequorivita-cap

MQDGIYAKITTEKGEILIKLTYDKTPGTVGNFVALAEGNLENSAKPQGTPYYDGLKFHRVIPDFMVQGGDPKGSGSGGPGYSFEDEFHPELRHDTPGVLSMANSGPASNGSQFFITHVATPWLDDKHTVFGNVVEGQDVVDSITQGDTMQKVEIIRVGEEAEKWNAVEGFRSFTGEREKNLAKKKQQEEDEIKKVSEGFDRTDSGLLYKMIVKGNGKKAEKGKTVSVHYKGMLTDGTEFDSSYKRKQPIDFPLGKGHVIEGWDEGIELLQVGDKARFVIPSHLAYGERGAGGVIPPGATLIFDVELMDVK

>Aequorivita-sp

MQDGIYAKITTEKGEILIKLTHDKTPGTVGNFVALAEGNLENSAKPQGTPYYDGLKFHRVIPDFMIQGGDPNGTGAGGPGYNFDDEFHQDLRHDTPGVLSMANAGPASNGSQFFITHVATPWLDNKHTVFGNVVEGQDVVDSVAQGDTMQKVEIIRVGEEAKKWNAVEAFRSFTGEREQRIAKMKEAQEAELKKISEGFDRTDSGLLYKIIQKGNGKKAEKGKTVSVHYKGALTDGTEFDSSYKRKQPIDFQLGVGQVIAGWDEGIQLLQVGDKARFVIPSHLAYGERGAGGVIPPSATLIFDVELMDVK

>Aequorivita-sub

MQDGIYAKITTEKGEILIKLTHDKTPGTVGNFVALAEGNLENKAKPQGTPYYDGLKFHRVLPDFMIQGGDPKGTGSGGPGYDFEDEFHQDLRHDTPGVLSMANSGPASNGSQFFITHVATPWLDNKHTVFGNVVEGQDVVDSVAQGDTMKKVEIIRVGEEAKNWNAVEAFRSFTGEREQRISKMKEAQEAELKKVSEGFDRTDSGLLYKIIQKGSGKKAEKGKTVSVHYKGALTDGTEFDSSYKRKEPIDFQLGVGQVISGWDEGVALLQVGDKARFVIPSHLGYGERGAGGVIPPNATLIFDVELMDVK

>Aequorivita-vla

MQDGIYAKITTEKGEILIKLTHDKTPGTVGNFVALAEGNLENSAKPQGTPYYDGLKFHRVIPDFMIQGGDPKGTGSGGPGYAFEDEFHQDLRHDTPGVLSMANAGPASNGSQFFITHVATPWLDNKHTVFGNVVEGQDVVDSIAQGDTMQKVEIIRVGEEAKNWNAVEAFRKFTGEREQRIAAKKAKEEAELKKVSEGFDRTDSGLLYKIIQKGNGKKAEKGKTVSVHYKGALTDGTEFDSSYKRKQPIDFQLGVGQVIAGWDEGIQLLQVGDKARFVIPSHLAYGERGAGGVIPPSATLIFDVELMDVK

>Algibacter-lec

MQDGLYAKFNTTKGEILVALEYKKTPGTVGHFVALAEGNLENKVKPQGTPYYDGLKFHRVIEDFMIQGGCPQGTGTGSPGYKFDDEFHPDLKHDGPGVLSMANAGPGTNGSQFFITHVETAWLDGKHTVFGKVVEGQNIVDAIAQGDLIDTLEIVRVGAEAEAFNGVEAFRTFEGSREAKLAAAKAAGEAELDKLAAGFEKTESGLRYQIIQEGNGVKAEKGKTVSVHYKGQLADGTVFDSSYKRNSPIDFAIGVGQVIPGWDEGIGLLKVGDKARLVIPSHLGYGSAGAGGVIPPNATLVFDVELMDVK

>Ancylostoma-cey

MTVANFVGLAEGNFTFQDGPSFNKPYYDGLKFHRVIADFMIQGGDPKGTGAGDPGYKFYDETRPDLRHDKAGVLSMANSGPATNGSQFFITHKETPWLDGKHTVFGHVVSGQDVVDAIKQDDVIETVKIIRVGNAAKKFDATKVFNAEYGKIFEEQKKEAEEIERISKMSQEEYRDFMYKEVLKKHPKAKLSATGLVYIIEKEGEGAAIEKGSPVSLHYTGTFRRDGGKFDSSYDRGQPMDFQFQVNRMIPGFEEGIAMLKKGGKAKLIIPYYQAYGPSGRPGAIPPFSDLIFDIEIVDVKDATVQKDDHHGHDHSDPNHKH

>Apibacter-men

MICKILIYCLLILTFINCKTLEIDKQMYNGLRNGLYANIKTSKGNMLVKFFDQESPVTVANFIGLAEGTIPNDAKKPGEPFYDGIIFHRVIKDFMIQTGDPQGTGMGGPGYRFEDEKNDLKHIGTGILSMANSGPNTNGSQFFITEVPTPWLDGKHTIFGEVIKGLETVNIIARVKTGAQDKPIDPVIIEKVSIITKGDEYKNYDAAQFFKDNKDLISNRNKKYLEEKQKVINAKLDELKLNMIQTSSGLFYKIDEKGSGAKAKSGDTVSVHYEGSLVNGNVFDSSFARNEPIEFPLGRGMVIKGWEEGISLLNEGDKATFLIPPSLGYGAQGAGGGVIPPNAWLIFKVELVKAK

>Aquimarina-aga

MQNGLYAKFNTTKGAILVNLEFEKTPGTVGNFVALAEGNLENSAKPQGEKYYDGLKFHRVIPDFMIQGGCPQGTGSGNPGYKFDDEIHPDLTHSGPGILSMANAGPGTNGSQFFITHVETAWLDGKHTVFGNVVEGQDVVDAIAQGDAIETMEIIRVGAAAEAFNAVEAFRLFNGVKAEREAAAKKESEALLADLAEGFDKTESGLHYKVINKGSGAQAEKGKTVSVHYKGSLPDGTVFDSSYKRNEPIDFPLGMGHVIAGWDEGIALLQVGDKARFVIPPHLGYGSQGAGGVIPPDATLVFDVELMDVK

>Aquimarina-lon

MQDGLYAKFNTTKGSILVNLEYKKTPGTVGNFVGLAEGNLENEVIPQGKPYYNGLKFHRVIADFMVQGGDPQGTGAGGPGYQFDDEIHPDLKHDGPGVLSMANAGPGTNGSQFFITHVETSWLDGKHTVFGKVVEGQDIVNAIEQGDEITSLEIQRVGAEAENFNAVETFRSFNGAKAEREAIAQKKSEELLQEVAAGFDKTDSGLRYKIIQNGDGVKAEKGKTVSVHYKGSLVDGTVFDSSYKRNQPIDFPLGMGQVISGWDEGIELLQVGDKARFVIPSHLAYGERGAGGVIPPNATLIFDVELMNVK

>Aquimarina-mac

MQEGLYAKFNTSKGSILVNLEYKKTPGTVGNFVGLAEGSIENQAIPQGKPYYDGLKFHRVIADFMIQGGDPQGTGAGGPGYQFDDEIHSDLKHDGPGVLSMANAGPGTNGSQFFITHVETSWLDGKHTVFGKVVEGLEIVNTIEQGDEIESIEIQRVGTEAESFNAVETFRSFNGAKAEREAAARKKTDDLLEELAAGFEKTDSGLRYKIIQNGDGVKAEKEKTVSVHYKGSLTDGTVFDSSYKRNQPIDFPLGVGQVIPGWDEGIALLQVGDKARFVIPPYLGYGDRGAGGVIPPNATLIFDVELVEVK

>Aquimarina-meg

MQDGLYAKFNTSKGSILVNLEYKKTPGTVGNFVGLAEGNIDNQAIPQGKPYYNGLKFHRVIADFMIQGGDPQGTGAGGPGYQFDDEIHSDLKHDGPGVLSMANAGPGTNGSQFFITHVETSWLDGKHTVFGKVVEGLEVVNTIEQGDEIESVEIQRVGAEAESFNAVETFRSFNGAKAEREAAARKKAEDLLEEIAAGFEKTDSGLRYKIIQKGDGAKAEKGKTVSVHYKGSLTDGTVFDSSYKRNQPIDFPLGMGQVISGWDEGIALLQVGDKARFVIPPYLGYGDRGAGGVIPPNATLIFDVELVEVK

>Aquimarina-pac

MQEGLYAKFNTTKGSILVNLEFEKTPGTVGNFVGLAEGTIENQVIPQGKPYYNGLKFHRVIADFMIQGGDPQGTGAGGPGYQFDDEIHPDLKHDGPGVLSMANAGPGTNGSQFFITHTETAWLDGKHTVFGKVVEGLDVVNAIEQGDEIESLEIQRVGAKAEGFNATETFRVFNGAKAEREAAAKKKAEDLLEEIAAGFDKTESGLRYQMIQKGDGVKAEKGKTVSVHYKGSLTDGTVFDSSFKRNQPIDFPLGVGQVIPGWDEGISLLHVGDKARFVIPPHLGYGANGAGGVIPPNATLIFDVELMEVK

>Aquimarina-spo

MQDGLYAKFKTTKGDILINLEYKKTPGTVGNFVGLAEGNLENQAIPQGKPYYNGLKFHRVIADFMIQGGDPQGTGAGGPGYQFDDEIHPDLKHDGPGVLSMANAGPGTNGSQFFITHVETPWLDGKHTVFGKVVEGQNVVNEVSQGDAIEEVEIIRIGGDAEGFNAVETFRQFNGAKAEREAAAKKKAEELLEELATGFDKTDSGLRYQIIQKGDGAKAEKGNTVSVHYKGSLPDGTVFDSSYKRNQPIDFPLGMGHVIPGWDEGIALLQVGDKARFVIPPYLGYGERGAGGVIPPNATLIFDVELVNVK

>Arenibacter-cer

MQDGIYAKFNTSKGEILVKLTHDKTPGTVGNFVALAEGTLENSVKPQGKPYYDGIKFHRVIPDFMIQGGCPLGTGTGDGGYKFDDEFHPELTHDGPGVLSMANAGPGTNGTQFFITHVATPWLDNKHTVFGHVVEGQDVVDSISQGDKIETLTIERVGEDAQNWNAIEAFRTFEGSREKRLAEEKRKVAAELDKVAAGFEETESGLRYKIIQKGNGAKPESGENVSVHYEGALLNGQVFDSSYKRKEPITFQLGVGQVIPGWDEGIQLLKVGDKARFVIPSSLAYGSAGAGGVIPPNATLVFDVELMGVS

>Arenibacter-pal

MQDGIYAKFNTSKGEILVKLTHDKTPGTVGNFVALAEGNLENSIKAQGKPYYDGLKFHRVIPDFMIQGGCPLGTGTGDGGYKFDDEFHPDLTHDGPGVLSMANAGPGTNGTQFFITHVATPWLDNKHTVFGHVVEGQDVVDAIKQGDKIESLTIERIGAEADKWNAVEAFRTFEGSREKRLAEEKKKIAAELDKVAAGFNETESGLRYKIVQEGKGPKSVSGKNVSVHYEGSLLNGQVFDSSYKRKEPISFQLGVGQVIPGWDEGIGLLKVGDKARFVIPSNLAYGSAGAGGVIPPNATLIFDVELMGIG

>Arenitalea-lut

MQDGLYAKFNTTKGEILIALEYTKTPGTVGNFVALAEGNLENKVKPQGTPYYDGLKFHRVIPDFMVQGGCPQGSGAGNPGYQFDDEFHPDLKHDGPGVLSMANAGPGTNGSQFFITHIETPWLDNKHTVFGKVEHGQEVVDAIAQGDKIESIEILRQGTEAEAFNAVEAFRTFEGSREKRLAEAKAAAEAELDKLAAGFSKTESGLRYQIIQEGAGVKAEKGKTVSVHYKGQLSDGTVFDSSYKRNAPIDFPLGVGQVIPGWDEGIQLLKVGDKARLVIPSNLAYGSAGAGGVIPPDATLVFDVELMDVK

>Bacteroidetes-bac-1

MNNGLYAKFNTSKGEILVNLEFKKTPGTVGNFVALAEGNMENGVKPQGTPYYDGLKFHRVIADFMIQGGCPQGTGTGNPGYSFDDEFHPELKHDKPGVLSMANSGPATNGSQFFITHVETPWLDNKHSVFGNVVEGQDIVDAIAQGDELTSIEIVRVGDAAEKFNAIEAFRKFEGAREARIAEALKAQDELLDGVSAGYTITDSGLRYTILQKGDGKQATKGAMVSVHYKGQLLDGTVFDSSYKRKQPIDFNVGIGQVISGWDEGIQLLKVGDKARFVIPSELAYGSRGAGGVIPPDAPLIFDVELMNVK

>Bacteroidetes-bac-2

MLLLLVASMQLTTYAQNKTQKNNMDNEFLANQVDGMYAKFETNKGNIYTVLEFKKTPMTVANFIGLAEGKIKNTARPDGTPYYDGLKFHRVIPNFMIQGGCPLGNGTGGPGYKFADEFDPTLRHTGPGILSMANAGPGTNGSQFFITHVATPWLDNKHTVFGHVIKGQDVVDAIVQNDSIISITILRKGAEAEAFDTPKVFEEEQVKAVQKAAEEAARLEAEAQKIKSQFTHSTPSGLRYDIIKEGTGKKPASAASKVTVHYTGTFLNGQVFDSSVQRGEPIEFGLNQVIPGWTEGVQLMNEGSVYKFYIPYQLAYGERGYPGAIPPKSDLIFEVELIKVNE

>Bergeyella-zoo

MNLKLISYIGLGISLIGCTPMYKKMNVDKELFNGLQDGVYANLQTSKGNMLVKFEDKKSPVTVANFVGLAEGKIDNKAKKAGEPFYDGTKFHRVIENFMIQGGDPQGTGMGDPGYRFDDEKNDLKHTGKGILSMANSGPNTNGSQFFITQVATPWLDGKHTVFGEVVHGLDVIDTIAKVEKGPQDKPVTDVVLEKVSVFTKGDEYKKYDAAKIFNEGKAKIQENNKAYLAKLEEEAKKQLEELSKGMEKTASGLFYKITQTNAEGKAPSKGSMVAVHYAGRLVNGTEFDNSFKRGEPIEFPVGTGRVIPGWDEGIMLLKEGEKATLLIPSELAYGARGAGGVIPPNAWLIFDVELVKVK

>Bizionia-arg

MKLLALALFVSFTSCSQQYPDLEDGLYAEFVTNKGTMVTKLFFNEVPVTVANFVSLAEGTHPDVSEDFKGKRYYDSLAFHRIIDKFMIQGGDPTGTGSGDPGYKFTDEFHPDLKHDKPGILSMANSGPNTNGSQFFITEVPTPHLDNKHSVFGEVVVGLDVLETISNVEVDPGNNKPLVPVVIEKLNIIRKGKAAEDFNAAEVYKEELPKVLEKQEALKEAAKQKLKESASESAEAFITKNADLGTVKKLDTGVVMIMTEEGENTKPRSTDKVLISYSGSFEDGRLFDTNWADVAKANNVYNEQRDTQGGYKPFAMIYNETAGLVPGFREAMLNMNVGNKATVFIPSYLGYGENGSGPIPPNSNLIFEVKIEGIQGE

>Borrelia-cro-1

MNKYLLFFILILMLVACASKRKNLMEEHGIFALIDTNKGAIKIKLYYKIAPLTVMNFIGLSEGLIENSVTNQPYFDNLIFHRVVDGFVIQTGDPTGTGTGGPGYVFPDEFTKGVRHDEAGVVSMANAGPDTNGSQFFITLADNLTYLDFKHSIFGKVVEGMETVRSISLGDKIERVKIIRVGDHANSFKVNNEEFLKLKSSYETKKLKEYEKHMAWQFEIIDRDCKDFEKDESGILYKVIKQGNGKCAKNGDIVRVDYEGFLLNGFKFDSSLERGNIMEFELGLGRVIKGWEIMLSHMCEGEERVIIIPPNLAYGDRDFTSMIKANSFLKFNIILRKVS

>Borrelia-cro-2

MEEHGIFALIDTNKGAIKIKLYYRIAPLTVMNFIGLSEGLIENSVTNQPYFDNLIFHRVVDGFVIQTGDPTGTGTGGPGYVFPDEFTKGVRHDEAGVVSMANAGPDTNGSQFFITLADNLTYLDFKHSIFGKVVEGMETVRSISLGDKIERVKIIRVGEHANSFKVNNEEFLKLKSSYETKKLKEYEKHMAWQFEIIDRDCKDFEKDESGILYKVIKQGNGKCAKNGDIVRVDYEGFLLNGFKFDSSLERGNIMEFELGLGRVIKGWEIMLSHMCEGEERVIIIPPNLAYGDRDFTSMIKANSFLKFNIILRKVS

>Borrelia-dut

MNKYLLFFILILMLVACASKRKNLMEEHGIFALIDTNKGTIKIKLYYKIAPLTVMNFIGLSEGLIENSVTNQPYFDNLIFHRVVDGFVIQTGDPTGTGTGGPGYVFPDEFTKGVRHDEAGVVSMANAGPDTNGSQFFITLADNLTYLDFKHSIFGKVVEGMETVRSISLGDKIERVKIIRVGDHAHSFKVNNEEFLKLKSSYETKKLKEYEKHMAWQFEIIDRDCKDFEKDESGILYKVIKQGNGKCAKNGDIVRVDYEGFLLNGFKFDSSLERGNIMEFELGLGRVIKGWEIMLSNMCEGEERVIIIPPNLAYGDRDFTSMIKANSFLKFNIILRKVN

>Borrelia-her

MSRYLLYFLLILMLVACGSKRTGLMERDGIFALIETNKGTIEVELYYKIVPLTVMNFIGLSEGAIKNSVTNRPYFENIVFHRVIDNFVIQTGDPTGTGTGGPGYVFPDEFNKDLSHNEPGIVSMANAGPDTNGSQFFITLKDNLTYLDFRHSIFGKVISGMDTVRSISQGDKIERVEIIRVGEDAKAFKVDNEGFLELKKSYEAKKIEEAEKYMASQLAIIDQDYKDFQRDKSGILYKINKKGNGKHVKNGNVLKVDYEGFLLSGVKFDSSIDRGEPIELVVGSGQVIEGWDIMLSDMCEGEDRVIIIPPSLAYGDKIVNEIIKANSFLKFNIILRKIN

>Borrelia-his

MNKYLLFFILILMLVACASKRRNLMEEHGIFALIDTNKGTMKIKLYYKIAPLTVMNFIGLSEGLIENSVTNQPYFDNLIFHRVVDEFVIQTGDPTGTGTGGPGYMFPDEFTKGVSHNEVGVVSMANAGPDTNGSQFFITLKDNLTYLDFKHSIFGKVIEGMDTARSISRGDKIERVRIIRVGDDANSFKVNNEEFLKLKSSYEAKKLKEYEKHMAWQFEIIDRDCKDFEKDESGILYKVIKQGNGKCAKNGDIVRVDYEGFLLTGFKFDSSLERGNIMEFELGSGRVIKGWEIMLSNMCEGDERVIIIPPNLAYGDRDFTSMIKANSFLKFNIILRKVN

>Borrelia-per

MNKYLLCFVIILMLVACSSKKEDLMKKNGIFALIDTNKGTIKIELYYKLAPLTVMNFIGLSECVIKNSVTNQPYFDNLIFHRVVDGFVVQTGDPTGTGTGGPGYVFPDEFSKDLSHNEAGIVSMANAGSDTNGSQFFITLSDNLTYLDYKHSIFGKVIEGMNIVQSISQGDKIEKVRIVRVGDDANSFKVDDEEFAKLKSSYEAKKREEVEKYMSLQLEIIDKDYKDFKKDKSGVLYKLTKSGSGKCVKDGSVVRVDYEGFLLNGVKFDSSIDRGNPIELVVGSGQVIEGWDVMLSNMCEGEERVIIIPPNLAYGNKQIGDLIKANSFLKFNIILRKVT

>Borrelia-rec

MNKYLLFFILILMLVACASKRKNLMEEHGIFALIDTNKGTIKIKLYYKIAPLTVMNFIGLSEGLIENSVTNQPYFDNLIFHRVVDGFVIQTGDPTGTGTGGPGYVFPDEFTKGVRHDEAGVVSMANAGPDTNGSQFFITLADNLTYLDFKHSIFGKVVEGMETVRSINLGDKIERVKIIRVGDHANSFKVNNEEFLKLKSSYETKKLKEYEKHMAWQFEIIDRDCKDFEKDESGILYKVIKQGNGKCAKNGDIVRVDYEGFLLNGFKFDSSLERGNIMEFELGLGRVIKGWEIMLSNMCEGEERVIIIPPNLAYGDRDFTSMIKANSFLKFNIILRKVN

>Borrelia-tur

MKRCLLYFLLILMLITCDSKRKDLVEKKGIFALIDTNKGNIEIELYYKIAPLTVMNFIGLSEGTIKNSVTSQPYFENIIFHRVVDEFVIQTGDPTGTGTGGPGYVFPDELNKNLSHNEPGIVSMANSGPDTNGSQFFITLADNLTYLDLKHSIFGKVVAGMDTVRNIRQGDKIEKVRIIRVGEDAKAFKVDNEEFLKLKKSYEARKIAEVKKYMASQLEIIDKDYKDFQKDESGILYKINKQGNGKNVKSGNIVKVDYEGFLLSGIKFDSSIDRGKPIEFVVGSGQVIEGWDVMLSDMCEEEERVVIIPPNLAYGERSIGNVIKPNSFLKFNIILRKIS

>Candidatus-rut

MTYRLFLLLTLIISLPSQAKLEEGLYANLHTNQGDIILKFEFEKTPLTVINFVGLAQGKKHSNIQIGKPFYNGLKFHRVIDNFIVQGGDPKGNGTGGPGYQFIDEITDDLKHDDGGILSMANSGPNTNGSQFFITYKAAPWLDGKHTVFGRVVEGMNVVNRIKQDDFIRKVNIIRIGEKAKNFQTDEAAFQATNAKYTSKEEKQLAHKKQSLVKFVNQNYPNTKLMTAGYFVEINQIGKDNQPKKGDLVKINLSIDLSDGTSIRKAEKPLQFAAGSGALIKLIDDEVLQMTLNEKRIIIASLNQIYSDNKRSNLSQDSILIFKLELLSINDIR

>Candidatus-thi

MRRLLVIFALLFSTFSIANLDDGIYAHLTTSKGEITVELAYKKAPLTVTNFIALSEGTKVSNKELGTPFYDGLVFHRVIDEFMIQGGDPKGNGTGGPGYMFADEFSDLIHDRPGVLSMANSGPNTNGSQFFITHVPTPWLDGKHSVFGSVVEGLDAVNSIKQGDILEAVRIERVGEEANQFAANEDSFNALISKANENILTSLKRRQKDFESYVSSTYPNAKETDLGYFTSVNKQGGGVSPNKGQIVSVDIALKANTGEVMRAAGSPIPFILGNGEIISIIEENAQEMTIGEERTVIATYESVFGDAPSGNIPQDAFIIFDLILIAAEDN

>Capnocytophaga-can-1

MQEGIYVKFITEKGDILIKLTHDKTPATVGNFVALVEGTQKNSRKPLGEPYYNGLKFHRVIPDFMIQGGCPLGTGTGSPGYQFDDEFHPELKHDRPGVLSMANAGPGTNGSQFFITHVPTPWLDNKHTVFGHVIEGQDVVDAIKQNDLMRELQIIRVGEAAQKWDAVTAFEKFNAEKAQRIEAERRKAEEILKNETIGFDKTESGLFYKIEQKGNGKQAQAGKTVRVHYTGMLLDKTIFDSSYKRNQPLEFVVGIGQVISGWDEGILLLQEGDKARFVIPSELAYGSRGAGGVIPPNAPLIFDVELVQVK

>Capnocytophaga-can-2

MKKMNFMMLLLASVSFLSCKTAKYPELGEGLFADIQTSKGDIVVKLHYKETPVTVANFVTLAEGKNKHVTEEYKGKPYYDGTIFHRIISGFMIQGGDPTGTGMGSPGYRFDDEIVESLKHNKKGILSMANAGPATNGSQFFITHVPTPFLDGRHTVFGETVLGDAVIDSIANVKTGANDRPEQAVTIKKIDIIRNGNEAIEFDAVKVFDNYVAERNRKEQEMEIKTKAQSEKFLSEIKVQESQAKVLPSGVKIFMLNEGNGEKPNNTQEVLVNYAGYLTNGTLFDSNIKLVNELYGKYDIKREEMEGYKPFPMPYHTSANLIPGFKEGLLTLKVGDKARVFIPSALAYGERGAGDVIPPNSDLIFDIEIVDIVQ

>Capnocytophaga-cyn

MQNGIYAKFKTTKGEILVELTYDKTPGTVGNFVALAEGKQKNTSKSLGEPYYNGLKFHRVIPDFMIQGGCPLGTGTGNPGYQFDDEFVPELKHDKPGVLSMANAGPGTNGSQFFITHVPTPWLDGKHTVFGQVVEGQNIVDSIVQGDKIEEISIVRVGEEAENWDAVKAFETFNAEKEKRITEERKKIEQALASETEGFEKTESGLFYKITNQGNGKKAKAGDEVAVHYTGMLLDKTVFDSSYRRNQPLIFTVGVGRVIEGWDEGILLLREGDKARFVIPPDLAYGSQGAGGIIPPNAPLIFDVELVKIVD

>Capnocytophaga-gin-1

MDITKLEEGIYAQIHTAKGDILLRLTYEKTPATVANFVALAEGKMKNSAKPIGVPFYDGLKFHRVIADFMIQGGCPQGTGTGTPGYRFDDEFHPDLKHDRPGILSMANAGANTNGSQFFITHRPTPHLDNKHTVFGYVVEGQKVVDTISQNDKIESVKIIRVGKKAEEWDALAAFEKFNAEKQARIEAEKRKQQEALAQLTEGFEKTASGLYYKITEKGNGKKPKKGDQVAVHYTGMLLDGKVFDSSLYRGQPLNFAVGIGQVIEGWDEGILLLNEGDKARLVIPSDLAYGSQGAGGVIPPNAALVFDVELVRVL

>Capnocytophaga-gin-2

MKKVKFWALLLPLLFVACKSAKYPDLKDGLYADIQTNHGDMLVELFYKATPGTVANFVSLAEGTNTYVADSLKGKRYYDGTKSHRVIKNFMLQAGDRTATGEGSPGYQFADEFVDSLKFTKKGQLAMANSGPATNGSQFFITEVATDWLTFRHTIFGQVVKGEEVISKITDEQTSKEDSRPKNPVIIKKIEIIRVGKDAQKWDAPKAFDAFMKEQDALAKKREEEAQEATKIRMEQAQRNLSLISEQEKQAKALPSGIKILSLNEGKGVKPQDGQKVLVNYAGYIRDTGMLFDSNIKEVAQENGVFDPQRAQDPQYGYMPYPWEYSQKVSLIAGFKEALLSMKVGDKLRVFIPAALAYGEQGIQGLIPPNSDLVFQIEIVDIAK

>Capnocytophaga-och

MQNGIYAKITTTKGVILLRLTYEKTPATVGNFVALAEGKMKNSQKPLGTPYYDGLTFHRVIPNFMIQGGCPLGTGTGDPGYRFDDEFHPELKHNKPGVISMANAGPGTNGSQFFITHVATPWLDNKHTVFGEVVEGQEVVDAIAQGDKIEKVEIVRVGEEAEKWDALKAFNDFNEAKAVRLAEEKRKAEEAFTKEVVGFEKTNSGLYYQITHKGNGKKAEAGQKVAVHYTGMLLDKTVFDSSYRRKEPLQFTVGVGQVIAGWDEGILLLHEGDKARLVIPSELAYGSRGAGGVIPPNAPLIFDVELVSVG

>Capnocytophaga-spu

MENGIYAKITTNKGVILLRLTYDKTPATVGNFVALAEGKMKNSQKPLGTPYYNGLTFHRVIPNFMIQGGCPLGTGTGDPGYRFDDEFHPELKHNKAGVISMANAGPGTNGSQFFITHLATPWLDNKHTVFGEVVEGQEVVDAIAQGDKIEKVEILRVGAEAEKWDALKAFEDFKAAKAARLAEEKRKAEEALAKEVVGFDKTDSGLYYQITHKGNGKKAVAGQKVAVHYTGMLLDKSVFDSSHRRREPLQFTVGVGQVIQGWDEGILLLSEGDKARLVIPSELAYGSRGAGGVIPPNAPLIFDVELVSVG

>Capnocytophaga-sp

MRRLNFIALLSAMFVFFSCNSQKKAYKDLGDGLFADIETTQGNIIVKLNYKETPVTVANFVTLAEGKNTFVKAEYKGKPFYNGTIFHRVIKDFMIQGGDPTGTGMGEPGYRFEDEIVPTLKHDKKGILSMANAGPATNGSQFFITQVPTPHLDGRHTVFGETVKGLEVIDAIANTKTVMNDKPEKDIKINKITIIANGKDAKNFNAVKVFEDYFKEINKREREKEAKTKAASAKFLEEVKVQEPQAKALPSGVKIFKLVDGKGKQPNHTHQVMVNYAGYLKNGTLFDSNVKEIEEAYGKYNSLREQQGGYQAFPMPYNTSAQLIPGFRDALLTMKVGDKIRVFIPAALGYGERGAGDVIPPNSDLIFDIEITDIAK

>Cellulophaga-alg-1

MKDGIYAKFNTSKGEILVKLTHDKTPGTVGNFVALAEGNLENKVKPQGTPYYDGLKFHRVIPDFMVQGGCPQGTGTGDAGYKFDDEFHVDLKHDGPGVLSMANAGPGTNGSQFFITHIATPWLDNKHTVFGNVVEGQDIVDAIKQGDKIDSLEIVRVGAEAEAFNAVEAFRTFEGSRAKRIEEERAKKSSELDEISAGFEETESGLRYKIIQKGNGKKAEAGMQVSVHYEGSLINGTVFDSSYKRKEPIDFQVGVGQVIAGWDEGICLLQVGDKARFVIPSDLGYGSAGAGGVIPPDATLIFDVELMKIS

>Cellulophaga-alg-2

MKNILSLFLIATVLVSSCKSSKTADLGDGIFADIETTQGDIIVKLEFDKTPVTVANFVSLAEGKNPFVTDSLKGKNYYDGLTFHRVMKDFMIQGGDPTASGMGNPGYRFIDEFADSLSHSKKGILSMANSGPKTNGSQFFITHKATPWLDNKHTVFGEVVSGIEVVDSIANVAVGQNNKPVDAVIMNKVKIIRNGKEARKFDAVAIMKQYFADEEVREKEALRIEAEREEKLNLATSSFVEAIEGQKKTAKTLASGLKIFTLEEGTGDKPKVGQTANLYYAGYFEDGRLFDSNVEETSTLYGQFNFNRRDQGGYEPMPMQISMDAPMIAGFKEAVMTMKVGQKIRVFIPSHLAYGESGRGPIPASTDLVFDLEMVSIGE

>Cellulophaga-bal

MKNILSLLLIATVLVSSCKSSKTADLGDGIFADIQTTQGDIIVKLEYDKTPVTVANFVSLAEGKNPFVTDSLKGKNYYDGLTFHRVMKDFMIQGGDPTGTGMGNPGYRFIDEFVDSLSHSKKGILSMANSGPKTNGSQFFITHKETPWLDNKHTVFGEVVNGMDVLDSIATVAVGENNKPVEAVIMNKVKIIRNGKEAKKFDAVAVMKKYFADEELREKEAARLEAEREQKLAEAKAAFVTNLESQKKAAQTLASGLKIFTLEEGTGEKPKVGQTANLYYAGYFEDGRLFDSNVEETSTLYGQFNWNRRDQGGYEPMPMVVSMDAPMIAGFKEAVMKMKVGQKARVFIPSHLAYGESGRGPIPANTDLIFDLEMVDIAQ

>Cellulophaga-geo

MQDGIYAKFNTTKGEILVKLTHDKTPGTVGNFVALAEGNLENSVKPQGTPYYNGLKFHRVIPDFMIQGGCPLGTGTGDAGYKFDDEFHPELTHSGPGVLSMANAGPGTNGSQFFITHIATPWLDNKHTVFGHVESGQDVVDAVAQGDTIETLEIVRVGEEAEKWNAVEAFRTFEGSREKRLAEEKAKAEAQMEKLAAGFDATESGLRYKIIQKGNGAKAESGKTVSVHYEGSLVSGQVFDSSYKRNQPIDFQLGVGQVIPGWDEGIALLQVGDKARFVIPSNLAYGSAGAGGVIPPNATLIFDVELMDVK

>Cellulophaga-lyt

MKKSFLILLIAVSAMVGCKSGQYADLEDGLYADIKTNKGDMLVKLEYNLAPVTVANFVSLAEGKSPFVSEEYKGKKYYDGIIFHRVMKDFMIQGGDPTGTGMGNPGYKFKDEFNDSLRHTKKGIISMANSGPKTNGSQFFITHAPTPFLDGLDEFGNLKNCDNPRVGCHSVFGEVVKGLEVVDSIANVKVAAKNRPIEDVVISTIEIVKKGKEAKKFDAIKVMTAYFEEEKEEEKRQLALAEEAKKTEAAFAAETAEQIKKAETLPSGLKVLVLKEGTGKKPKIGQKALVNYAGWLSNGTLFDTTMEDVAKEFGKYDIIYKMHGNDLSPYPMEYSPESRLTAGFKEGLLRMRVGDKVRLFIPPHLGYGEQGGGPIPPNADLIFDVEITGIQ

>Cellulophaga-sp

MQDGIYAKIHTPKGEITLKLEHEKTPGTVGNFVALAEGNLENSQKPQGTPYYDGLKFHRVIPDFMIQGGCPLGTGTGDGGYKFDDEIHPDLKHDAPGKLSMANAGPGTNGTQFFITHIPTDWLDGKHTVFGNVVEGQDVVNSVAQGDEMEKIEIIRVGAEAEAWNAVEAFRTFEGSRAKRIEEERAAQAAEIEALATGFDTTDSGLRYQIIQKGDGVKAEKGKTVSVHYKGALPDGTVFDSSFKRNQPIDFQLGVGQVIPGWDEGIALLNVGDKARLVIPSDLAYGSAGAGGVIPPNATLVFDVELVAVK

>Chitinivibrio-alk

MKIDFSHMVLSQKGGRMNKIGVTILLGLLSFAVFSKESSVNEGVFAQMKTSKGSITIQLYYDKTPLTVANFVGLAEGNISNTAQDEGDPFYDRVVFHRVVDGFVVQGGDPTGTGRGGPGYQFPDEIVPGLTHSKAGILSMANAGPGTNGSQFFITLDAQPHLDGKHTVFGEVVSGMDVVNSLRQGDTIRYVDIERVGSTAQEFSADQDHFDQLLADVKEEETRRQEKERDAQAASIAEKYPDAQKSEAGFFYIREAEGTGDTPQKGATVSVHYTGSFLDGQVFDSSRRRGTPLEFSLGMGEVISGWDAALLEMKQGEKRTIILPPELAYGEQGAGGVIPPNAWLVFEVELIDF

>Chryseobacterium-ang

MKLFNKNIILAAASISLMSCTPIYKKMNVDKETYEGLNDGLYANLQTSKGNLIVQFEDKKAPVTVANFIGLAEGKIDNKAKAKGVPYYDGTIFHRVIKDFMIQGGDPQGTGAGDPGYKFEDEKNDLKHTGKGILSMANSGPNTNGSQFFITEVATPWLDGRHTIFGKVVKGNDVIDAIANVEKGAQDKPKTDVVLEKVSVFGKGDAYKNYDAAKTFTEGKAKIAENNKAYIAKEEAERKKKEEEFKANQEKMVENLKAGMQKTESGLYYKITKTVDGAKAPKSGDNVSVHYAGKLIDGTEFDSSFKRNEPIEIPIGMGRVIKGWDEGILLLKEGETATLLIPPAMAYGERGAGGVIPPNAWLVFDVELVKVQ

>Chryseobacterium-ant

MLKKNLLIAIAAISIASCTPIYKKMNVDKETYAGLKEGLYANFQTSKGNMIVKFEDKKAPVTVANFVGLAEGKIDNTAKGKGVPFYDGTIFHRVIKDFMIQGGDPKGTGMGDPGYKFDDERNDLQHTGKGILSMANSGPNTNGSQFFITEVPTPHLDGRHTIFGEVVNGLEVVDSIATVEKGAQDKPKTDVVLEKVAIFSKGDDYKTYDAAKIFSEGKGKIKENNKAILDKIEADKKKKAEEFAANQQKLVDDLKATMQVIPSGLYYKITEKTEGKKANVGDNVSVHYAGKLVDGTEFDSSFKRNEPIEIPIGVGQVIKGWDEGILLLNEGESATLLIPSELGYGANGAGGIIPPNAWLIFDVKLVKVNSISTK

>Chryseobacterium-aqu

MKLFNKNIILAAASVSLLSCTPIYKKMNVDKETYEGLKDGLYANLQTTKGNLIVQFEDKKSPVTVANFVGLAEGKIDNKAKAKGVPFYDGTIFHRVIKDFMIQGGDPQGTGMGDPGYKFEDEKNDLKHTGKGILSMANSGPNTNGSQFFITEVATPWLDGRHTIFGKVVKGDDVIDAIANVEKGAQDKPKTDVVLEKVSIFSKGDEYKGYDAAKTFNEGKAKIAANNKAMAEKAEAEAKKALEDLKAGMQVTESGLYYKITKKTEGKAPKAGDNIQVHYAGKLTNGTEFDSSFKRGEPLEFPVGTGRVIKGWDEGILLLKEGETATLLIPPAMGYGERGAGGVIPPNAWLIFDVELVKVP

>Chryseobacterium-ara

MKLFNKNIILAAASISLMSCTPIYKKMNVDKETYEGLNDGLYANLQTTKGNLIVKFEDKKAPVTVANFIGLAEGKIDNKAKAKGVPYYDGTIFHRVIKDFMIQGGDPQGTGMGDPGYKFEDEKNDLKHTGKGILSMANSGPNTNGSQFFITEVATPWLDGRHTIFGKVVKGEEVIDAIANVEKGAQDKPKTDIVLEKVSVFSKGDEYKNYDPAKTFTEGKAKIAENNKIAIAKEEADKKKKEEEFAANQLKMVEDLKAGMQKTESGLYYKITKTADGKAPKSGDNVSVHYAGKLVDGTEFDSSFKRNEPIEIPIGMGRVIKGWDEGILLLKEGETATLLIPPAMGYGERGAGGVIPPNAWLVFDVELVKVK

>Chryseobacterium-arth

MKLFNKNIILAAASISLMSCTPIYKKMNVDKETYEGLNDGLYANLQTTKGNMIVKFEDKKAPVTVANFIGLAEGKIDNKAKAKGVPYYDGTIFHRVIKDFMIQGGDPQGTGMGDPGYKFEDERNDLKHTGKGILSMANSGPNTNGSQFFITEVATPWLDGRHTIFGKVVKGNDVIDAIANVEKGAQDKPKTDIVLEKVSVFSKGDEYKNYDAAKTFNEGKAKIAENNKAFIAKEEAEKKKKEEEFKANQEKLVESLKAGMQKTESGLYYKITKTADGKAPKAGDNVSVHYAGKLVDGTEFDSSFKRNEPIEIPIGMGRVIKGWDEGILLLKEGETATLLIPPAMAYGERGAGGVIPPNSWLVFDVELVKVK

>Chryseobacterium-arto

MKLFNKNIILAAASISLMSCTPIYKKMNVDKETYEGLNDGLYANLQTTKGNMIVKFEDKKAPVTVANFIGLAEGKIDNKAKAKGVPYYDGTIFHRVIKDFMIQGGDPQGTGMGDPGYKFEDERNDLKHTGKGILSMANSGPNTNGSQFFITEVATPWLDGRHTIFGKVVKGIDVIDTIANVEKGAQDKPKTDIVLEKVSIFGKGDEYKNYDAAKTFNEGKAKIAENNKAFIAKEEAEKKKREEEFKANQEKLVEGLKAGMQKTESGLYYKITKTADGKAPKAGDNVSVHYAGKLVDGTEFDSSFKRNEPIEIPIGMGRVIKGWDEGILLLKEGETATLLIPPAMGYGERGAGGVIPPNSWLIFDVELVKVK

>Chryseobacterium-bal

MKFLNKNIILAAASVSLLSCTPIYKKMNVDKETYEGLKDGLYANIQTTKGNLIVKFEDKKSPVTVANFVGLAEGKIDNKSKAKGVPFYDGTIFHRVIKDFMIQGGDPQGTGMGDPGYKFEDEKNDLKHTGKGILSMANSGPNTNGSQFFITEIATPWLDGKHTIFGEVVKGDDVIDAIANVEKGAQDKPKTDIVLEKVSIFSKGDEYKGYDAAKTFTEGKGKIAANNKAMAEKAEADAKKALEDLKAGMQVTESGLYYKITKKTEGKAAKAGDNVQVHYAGKLTNGTEFDSSFKRNEPLEFPVGTGRVIKGWDEGILLLKEGETATLLIPPAMGYGERGAGGVIPPNAWLIFDVELVKVP

>Chryseobacterium-bov

MKKIIALSVTLLTLLNCKTLEIDKETYKSLPDGLYGNLTTSKGDILVKFEDQKSPVTVANFVGLAEGKIENKAKKKGEPFYDGTIFHRVIKDFMIQGGDPKGTGMGDPGYKFDDEKNDLQHTGKGILSMANSGPNTNGSQFFITEVATPWLDGRHTIFGKVVKGEAVIDSIANVEKGPQDKPKTDIVLNKVAIFGKGDQYKHYDAAKIFNEGKSKIQDNNKVYLAKAEEEKQRKLKEFAENQEKLVNDLKAGMQSTPSGLFYKITKTTAGAAPVAGDQVSVHYAGKLVSGEEFDSSFKRNEPIDIPIGVGQVIKGWDEGILLLKEGETATFLIPPSLGYGERGAGGVIPPNAWLIFDVELVKINK

>Chryseobacterium-cae

MKKIIALSITLLTLLTLLNCKTLEIDKEVYKSLPDGLYGNFVTSKGDILVKFEDEKSPVTVANFVGLAEGKIENKSKKKGEPFYDGTIFHRVIKDFMIQGGDPQGTGMGDPGYKFADEKNDLKHTGKGILSMANSGPNTNGSQFFITEVATPWLDGRHTIFGKVVGGEAVIDSIANVEKGAQDKPKTDVVLTKVAVFGKGDKYKHYDAAKIFTEGKDKIEEKNKAYLAKAEEEKKRKEEEFKANQQKLVDDLKAGMQSTPSGLFYKITKTTTGAAPTAGQTVAVHYAGKLVNGEEFDNSFKRGQPIDIPIGVGQVIKGWDEGIMLLKEGETATLLIPPALGYGERGAGGVIPPNAWLIFDVELVKIGG

>Chryseobacterium-car

MKLFNKNIILAAASISLMSCTPIYKKMNVDKETYEGLNDGLYANLQTSKGNLIVQFEDKKAPVTVANFIGLAEGKIDNKAKAKGVPFYDGTIFHRVIKDFMIQGGDPQGTGMGDPGYKFEDEKNDLKHTGKGILSMANSGPNTNGSQFFITEVATPWLDGRHTIFGKVVKGNDVIDAIANVEKGAQDKPKTDIVLEKVSVFGKGDAYKNYDAAKTFTEGKAKIAENNKAYIAKEEADKKKKEEEFKANQEKMVEDLKAGMQKTESGLYYKITKTVAGAKAPKSGDNVSVHYAGKLIDGTEFDSSFKRNEPIEIPIGMGRVIKGWDEGILLLKEGETATLLIPPAMAYGERGAGGVIPPNAWLIFDVELVKVQ

>Chryseobacterium-cha

MLKKNFLIAIAALSLTSCNSIYKKMNVDKETYEGLKEGLYANFQTSKGNMIVKFEDKDAPVTVANFVGLAEGKIDNKAKAKGVPFYDGTIFHRVIKDFMIQGGDPLGTGMGDPGYKFDDEKNSLQHTGKGILSMANSGPNTNGSQFFITEVATPWLDGRHTIFGKVVNGLDVIDVIAKVDKGAQDKPKTNVVLEKVSVFSKGDEYKGYDAAKIFAEGKAKITEGNKAIMAKIEADKKKKEEEFAANQQKLVDDLKATMQSTPSGLYYKITKKTEGVAPKKGEDVAVHYAGKLVDGTEFDSSFKRNQPIDIPIGVGQVIKGWDEGIMLLKEGETATLLIPSELGYGANGAGGVIPPNAWLIFDVELVKVKSILSK

>Chryseobacterium-con

MKLFNKNIILAAASVSLMSCTPIYKKMNVDKETYEGLNDGLYANLQTTKGNMIVKFEDKKAPVTVANFIGLAEGKIDNKAKAKGVPYYDGTIFHRVIKDFMIQGGDPQGTGMGDPGYKFEDERNDLKHTGKGILSMANSGPNTNGSQFFITEVATPWLDGRHTIFGKVVKGNDVIDAIANVEKGAQDKPKTDIVLEKVSVFSKGDEYKNYDAAKTFNEGKAKIAENNKAFIAKEEAERKKKEEEFKANQEKLVESLKAGMQKTESGLYYKITKTADGKAPKAGDNVSVHYAGKLVDGTEFDSSFKRNEPIEIPIGMGRVIKGWDEGILLLKEGETATLLIPPAMAYGERGAGGVIPPNSWLVFDVELVKVK

>Chryseobacterium-cuc

MKLFNKNIILAAASISLMSCTPIYKKMNVDKETYEGLNDGLYANLQTTKGNMIVKFEDKKAPVTVANFIGLAEGKIDNKAKAKGVPYYDGTIFHRVIKDFMIQGGDPQGTGMGDPGYKFEDERNDLKHTGKGILSMANSGPNTNGSQFFITEVATPWLDGRHTIFGKVVKGSEVIDAIANVEKGAQDKPKTDIVLEKVSVFSKGDEYKHYDAAKTFNEGKAKIAENNKAFIAKEEAEKKKREEEFKANQEKLVENLKAGMQKTESGLYYKITKTADGKAPKAGDNVSVHYAGKLVDGTEFDSSFKRNEPIEIPIGMGRVIKGWDEGILLLKEGETATLLIPPAMAYGERGAGGVIPPNSWLVFDVELVKVK

>Chryseobacterium-cul

MKLFNKNIILAAASISLMSCTPIYKKMNVDKETYEGLNDGLYANLQTTKGNLIVKFEDKKAPVTVANFIGLAEGKIDNKAKAKGVPYYDGTIFHRVIKDFMIQGGDPKGTGAGDPGYKFEDERNDLKHTGKGILSMANSGPNTNGSQFFITEVATPWLDGRHTIFGKVVKGEDVIDAIANVEKGAQDKPKTDIVLEKVSVFSKGDEYKHYDAAKTFNEGKSKIAENNKAFIAKEEAEKKKKEEEFKANQEKLVENLKAGMQKTESGLYYKITKTADGKAPKAGDNVSVHYAGKLVDGTEFDSSFKRNEPIEIPIGMGRVIKGWDEGILLLKEGETATLLIPPAMAYGERGAGGVIPPNSWLVFDVELVKVK

>Chryseobacterium-dae

MKLFNKNIILAAASVSLMSCTPIYKKMNVDKETYEGLNDGLYANLQTTKGNMIVKFEDKKAPVTVANFIGLAEGKIDNKAKAKGVPYYDGTIFHRVIKDFMIQGGDPQGTGMGDPGYKFEDEKNDLKHTGKGILSMANSGPNTNGSQFFITEVATPWLDGRHTIFGKVVKGEDVIDAIANTEKGANDKPKTDIVLEKVSVFSKGDEYKNYDPAKTFTEGKAKIAENNKAAMAKEEADKKKKEEEFKANQEKMVENLKAGMQKTESGLYYKITKTTTGAAPKAGDNVSVHYAGKLVDGTEFDSSFKRNEPIEIPIGMGRVIKGWDEGILLLKEGETATLLIPPAMGYGERGAGGVIPPNAWLVFDVELVKVK

>Chryseobacterium-for

MKFLNKNIILAAASVSLLSCTPIYKKMNVDKETYEGLKDGLYANIQTTKGNMIVKFEDKKSPVTVANFVGLAEGKIDNKAKAKGVPFYDGTIFHRVIKDFMIQGGDPQGTGMGDPGYKFEDEKNDLKHTGKGILSMANSGPNTNGSQFFITEVATPWLDGRHTIFGSVVSGTETIDAIAAVEKGAQDKPKTDIVLEKVSIFSKGDEYKNYDAAKTFNEGKGKIAANNKAMAEKAEAEAKKALEELKAGMQVTESGLYYKITKKTDGKAAKAGDNVKVHYAGKLTNGTEFDSSFKRNEPLEFPVGTGRVIKGWDEGILLLKEGETATLLIPPAMGYGERGAGGVIPPNAWLIFDVELVKVP

>Chryseobacterium-gal

MKLFNKNIILAAASVSLMSCTPIYKKMNVDKETYEGLNDGLYANLQTTKGNMIVKFEDKKAPVTVANFIGLAEGKIDNKAKAKGVPYYDGTIFHRVIKDFMIQGGDPQGTGMGDPGYKFEDEKNDLKHTGKGILSMANSGPNTNGSQFFITEVATPWLDGRHTIFGKVVKGTEVIDAIANVEKGAQDKPKTDIVLEKVSVFSKGDEYKNYDAAKTFNEGKAKIAENNKAFIAKEEAEKKKREEEFRANQEKLVENLKAGMQKTESGLYYKITKNTDGKAPKAGDNVSVHYAGKLVDGAEFDSSFKRNEPIEIPIGTGRVIKGWDEGILLLKEGETATLLIPPAMAYGERGAGGVIPPNSWLIFDVELVKVK

>Chryseobacterium-gam

MKLFNKNIILAAASVSLMSCTPIYKKMNVDKETYEGLNDGLYANLQTTKGNLIVKFEDKKAPVTVANFIGLAEGKIDNKAKAKGVPYYDGTIFHRVIKDFMIQGGDPQGTGMGDPGYKFEDERNDLKHTGKGVLSMANSGPNTNGSQFFITEVATPWLDGRHTIFGKVVKGEDVIDAIANVEKGAQDKPKTDIVLEKVSVFSKGDEYKNYDAAKTFTEGKAKIAENNKAFLAKEEAEKAKKEAEFKANQEKMVEDLKAGMQKTESGLYYKITKTTEGKAPKAGDNVSVHYAGKLVDGSEFDSSFKRNEPIEIPIGMGRVIKGWDEGILLLKEGETATLLIPPAMGYGERGAGGVIPPNAWLIFDVELVKVK

>Chryseobacterium-gle

MKLFNKNIILAAASISLMSCTPIYKKMNVDKETYEGLNDGLYANLQTTKGNMIVKFEDKKAPVTVANFIGLAEGKIDNKAKAKGVPYYDGTIFHRVIKDFMIQGGDPQGTGMGDPGYKFEDERNDLKHTGKGILSMANSGPNTNGSQFFITEVATPWLDGRHTIFGKVVKGTEVIDAIANVEKGAQDKPKTDIVLEKVSVFSKGDEYKHYDAAKTFNEGKAKIAENNKAFIAKEEAERKKKEEEFKANQEKLVESLKAGMQKTESGLYYKITKTADGKAPKAGDNVSVHYAGKLVDGTEFDSSFKRNEPIEIPIGMGRVIKGWDEGILLLKEGETATLLIPPAMAYGERGAGGVIPPNSWLVFDVELVKVK

>Chryseobacterium-gree

MKLFNKNIILAAASVSLLSCTPIYKKMNVDKETYEGLKDGLYANLQTTKGNLIVQFEDKKSPVTVANFVGLAEGKIDNKAKAKGVPFYDGTIFHRVIKDFMIQGGDPQGTGMGDPGYKFEDEKNDLKHTGKGILSMANSGPNTNGSQFFITEVATPWLDGRHTIFGKVVKGDDVIDAIANVEKGAQDKPKTDVVLEKVSIFSKGDEYKGYDAAKTFNEGKAKIAANNKAMAEKAEAEAKKALEDLKAGMQVTESGLYYKITKKTEGKAPKAGDNIKVHYAGKLTNGTEFDSSFKRGEPLEFPVGTGRVIKGWDEGILLLKEGETATLLIPPAMGYGERGAGGVIPPNAWLIFDVELVKVP

>Chryseobacterium-greg

MKLFNKNIILAAASISLMSCTPIYKKMNVDKETYEGLNDGLYANLQTTKGNLIVKLEDKKAPVTVANFVGLAEGKIDNKAKAKGVPFYDGTIFHRVIKDFMIQGGDPQGTGMGDPGYKFEDEKNDLQHTGKGILSMANSGPNTNGSQFFITEVATPWLDGKHTIFGKVVQGNDVIDAIANVEKGPQDKPKTDIVLEKVSVFSKGDEYKNYDPAKTFNEGKAKIAENNKAAMAKAEADKKKKEEEFKANQEKMVEDLKAGMQKTESGLYYKITKTAEGKAPKAGDNVSVHYAGKLVDGTEFDSSFKRNEPIDIPIGMGRVIKGWDEGILLLKEGEAATLLIPPAMGYGANGAGGVIPPNAWLVFDVELVKVK

>Chryseobacterium-hai

MIKKNFLIALASILLTSCTPIYKKMNVDKETYEGLNDGLYANFQTSKGNMIVKFEDKKAPVTVANFVGLAEGKIENKAKAKGVPFYDGTIFHRVIKDFMIQGGDPQGTGMGDPGYKFDDEKNDLQHTGKGILSMANSGPNTNGSQFFITEVATPWLDGRHTIFGEVVKGEEVIDAIANVEKGAQDKPKTDVVLEKVSIFSKGDEYKDYDAAKVFNDGKGKIQENNKAILAKIEADKKKKEEEFAANQQKLVDDLKAGMQVTPSGLYYKITKTTNGVAPKAGDEVSVHYAGKLVDGSEFDSSFKRNQPIEIPIGVGQVIKGWDEGILLLKEGEAATLLIPSELGYGARGAGGVIPPNAWLIFDVELVKVNGSAVK

>Chryseobacterium-hun

MKKIIALSITLLTLINCKTLEIDKEVYKSLPDGLYGNLVTSKGDILVKFEDEKSPVTVANFVGLAEGKIENKSKKKGEPFYDGTIFHRVIKDFMIQGGDPQGTGMGDPGYKFADEKNDLQHTGKGILSMANSGPNTNGSQFFITEVATPWLDGRHTIFGKVVRGEAVIDSIANVEKGAQDKPKTDIVLTKVAVFGKGDKYKHYDASKVFAEGKEKIEEKNKAYLTKAEEEKKRKEEEFKANQQKLVDNLKAGMQSTPSGLFYKITKTTTGAAPTAGQTVAVHYAGKLVNGEEFDNSFKRGQPIDIPIGVGQVIKGWDEGIMLLKEGETATLLIPPTLGYGERGAGGVIPPNAWLVFDVELVKIGG

>Chryseobacterium-indolo

MKLFNKNIILAAASVSLMSCTPIYKKMNVDKETYEGLNDGLYANLQTTKGNMIVKFEDKKAPVTVANFIGLAEGKIDNKAKAKGVPYYDGTIFHRVIKDFMIQGGDPQGTGMGDPGYKFEDERNDLKHTGKGILSMANSGPNTNGSQFFITEVATPWLDGRHTIFGKVVKGNDVIDAIANVEKGAQDKPKTDIVLEKVSIFGKGDEYKNYDAAKTFNEGKAKIAENNKAFIAKEEAEKKKKEEEFKANQEKLVEGLKAGMQKTESGLYYKITKTADGKAPKAGDNVSVHYAGKLVDGTEFDSSFKRNEPIEIPIGMGRVIKGWDEGILLLKEGETATLLIPPAMAYGERGAGGVIPPNSWLVFDVELVKIK

>Chryseobacterium-indolt

MKFLNKNIILAAASISLLSCTPIYKKMNVDKETYEGLKDGLYANLQTTKGNLIVKFEDKKSPVTVANFVGLAEGKIDNKSKAKGVPFYDGTIFHRVIKDFMIQGGDPKGTGAGDPGYKFEDEKNDLKHTGKGILSMANSGPNTNGSQFFITEVATPWLDGRHTIFGQVVKGDDVIDAIANVEKGAQDKPKTDIVLEKVSIFSKGDEYKGYDAAKTFNEGKGKIAENNKAFIAKEEAEKVKKEEEFKANQLKLVEGLKEGMQVTESGLYYKITKKTDGKAPKAGDNVFVHYAGKLTNGEEFDSSYKRNQPLDFPVGTGRVIKGWDEGILLLKEGETATLLIPPAMGYGERGAGGVIPPNAWLIFDVELVKVP

>Chryseobacterium-jej

MKLFNKNIILAAASVSLMSCTPIYKKMNVDKETYEGLNDGLYANLQTTKGNLIVKFEDKKAPVTVANFIGLAEGKIDNKAKAKGVPYYDGTIFHRVIKDFMIQGGDPQGTGMGDPGYKFEDERNDLKHTGKGILSMANSGPNTNGSQFFITEVATPWLDGRHTIFGKVVKGNDVIDTIANVEKGAQDKPKTDIVLEKVSIFGKGDEYKNYDAAKTFNEGKAKIAENNKAFIAKEEAEKKKKEEEFKANQEKLVENLKAGMQKTESGLYYKITKTADGKAPKAGDNVSVHYAGKLVDGTEFDSSFKRNEPIDIPIGMGRVIKGWDEGILLLKEGETATLLIPPAMGYGERGAGGVIPPNSWLVFDVELVKVK

>Chryseobacterium-jeo

MLKKNFLIAVAALSLTSCTSIYKKMNVDKETYEGLKEGLYGNFQTSKGNMIVKFEDKDAPVTVANFVGLAEGKIDNKAKAKGVPFYDGTIFHRVIKDFMIQGGDPQGTGMGDPGYKFDDEKNNLQHTGKGILSMANSGPNTNGSQFFITEVATPWLDGKHTIFGKVVNGLDIVDVIANVDKGPQDKPKTNVVLEKVSIFSKGDQYKGYDAAKIFTEGKSKIAEDNKAMMAKIEADKKKKEEEFAGNQQKLVDDLKATMKSTPSGLFYKITKKTDGVVPQKGDEVSVHYAGKLVDGTEFDSSFKRNQPIDIPIGVGQVIKGWDEGILLLKEGETATLLIPSELGYGANGAGGVIPPNAWLIFDVELVKVKSNLSK

>Chryseobacterium-joo

MKLFNKNIILAAASISLMSCTPIYKKMNVDKETYEGLNDGLYANLQTTKGNLIVKFEDKKAPVTVANFIGLAEGKIDNKAKAKGVPYYDGTIFHRVIKDFMIQGGDPQGTGMGDPGYKFEDERNDLKHTGKGILSMANSGPNTNGSQFFITEVATPWLDGRHTIFGKVVKGNDVIDAIANVEKGAQDKPKTDIVLEKVSVFGKGDEYKNYDAAKTFNEGKAKIAENNKAFIAKEEAEKKKKEEEFKANQEKLVENLKAGMQKTESGLYYKITKTADGKAPKSGDNVSVHYAGKLVDGTEFDSSFKRNEPIEIPIGMGRVIKGWDEGILLLKEGETATLLIPPAMGYGERGAGGVIPPNSWLVFDVELVKVK

>Chryseobacterium-kor

MIKKSFLIALAAITLTHCTPIYKKMNVDKETYEGLKDGLYANFQTSKGNMIVKFEDQKAPVTVANFIGLAEGKIDNSAKAKGVPYYDGTIFHRVIKDFMIQGGDPQGTGMGDPGYKFDDERNDLQHTGKGILSMANSGPNTNGSQFFITEVATPWLDGKHTIFGEVVKGQNVIDEIANVEKGPQDKPKTDIVLEKVSVFSKGDAYKNYDAAKTFTEGKAKIQENNKVILEKIEAERIKKEEEFAANQQKLVDELKAGMQSTPSGLYYKITKTTDGVKPNPGETVSVHYAGRLVDGTEFDSSFKRNQPIEIPIGVGQVIKGWDEGIMLLKEGETATLLIPSELGYGSRGAGGVIPPNAWLVFDVELVHVKSNATK

>Chryseobacterium-lim

MKLFNKNIILAAASVSLMSCTPIYKKMNVDKETYEGLKDGLYANLQTTKGNLIVKFEDKKSPVTVANFVGLAEGKIDNKAKAKGVPFYDGTIFHRVIKDFMIQGGDPKGTGMGDPGYKFEDEKNDLHHTGKGILSMANSGPNTNGSQFFITEVATPWLDGKHTIFGSVVKGNDVIDAIANVEKGAQDKPKTDIVLEKVSIFSKGDEYKNYDAAKTFTEGKAKIAENNKAYIAKEEADRKKKEEEFKANQLKMVEDLQAGMQKTESGLYYKITKTTTGKAPKAGDNISVHYAGKLVDGSEFDSSFKRNEPIEIPIGMGRVIKGWDEGILLLKEGETATLLIPPALGYGERGAGGVIPPNAWLVFDVELVKVQ

>Chryseobacterium-lut

MKLFNKNIILAAASISLMSCTPIYKKMNVDKETYEGLHDGLYANLQTSKGNLIVQFEDKKAPVTVANFIGLAEGKIDNKAKGKGVPYYDGTIFHRVIKDFMIQGGDPQGTGMGDPGYKFEDEKNDLKHTEKGILSMANSGPNTNGSQFFITEVATPWLDGRHTIFGRVIKGNDVIDAIANVEKGAQDKPKTDIVLEKVSVFSKGDEYKNYDAAKTFNEGKAKIAENNKAYIAKEEADRKKKEEEFKANQDKMVENLKAGMQKTESGLYYKITKTVAGAKAPKSGDNVSVHYAGKLIDGTEFDSSFKRNEPIEIPIGMGRVIKGWDEGILLLKEGETATLLIPPAMAYGERGAGGVIPPNAWLIFDVELVKVQ

>Chryseobacterium-mol

MKKIIALSITLLTLLNCKTLEIDKDTYKSLPDGLYGNFVTSKGEILVKFEDEKSPVTVANFVGLAEGKIENKSKKKGEPFYDGTIFHRVIKDFMIQGGDPQGTGMGDPGYKFDDEKNDLQHTGKGILSMANSGPNTNGSQFFITEVATPWLDGRHTIFGKVIKGEAVIDSIANVEKGAQDKPKTDIVLNKVAVFAKGNAYNHYDAAKIFSEGKSKIQDKNKVYLAKEEEEKAKKQKEFAENQERLVNEMKAGMQSTASGLYYKITKNSTGATPTAGQTVSVHYAGKLINGEEFDNSFKRGQPIDIPIGVGQVIKGWDEGILLLKEGEAATLLIPPALGYGERGAGGVIPPNSWLVFDVELVKIAK

>Chryseobacterium-ole

MKLFNKNIILAAASISLMSCTPIYKKMNVDKETYEGLNDGLYANLQTSKGNLIVQFEDKKAPVTVANFIGLAEGKIDNKAKAKGVPFYDGTIFHRVIKDFMIQGGDPKGTGMGDPGYKFEDEKNDLKHTGKGILSMANSGPNTNGSQFFITEVATPWLDGRHTIFGKVVKGNDVIDAIANVEKGAQDKPKTDIVLEKVSIFGKGDAYKNYDAAKTFTEGKAKIAENNKAYIAKEEADRKKKEEEFKANQEKMVEDLKAGMQKTESGLYYKITKTVAGAKAPKSGDNVSVHYAGKLIDGTEFDSSFKRNEPIEIPIGMGRVIKGWDEGILLLKEGETATLLIPPAMAYGERGAGGVIPPNAWLVFDVELVKVQ

>Chryseobacterium-ora

MKLFNKHIILAAASISLMSCTPIYKKMNVDKETYEGLHDGLYANLQTSKGNLIVQFEDKKAPVTVANFIGLAEGKIDNKAKAKGVPYYDGTIFHRVIKDFMIQGGDPQGTGMGDPGYKFEDEKNDLKHTGKGILSMANSGPNTNGSQFFITEVATPWLDGRHTIFGKVVKGNDVIDAIANVEKGAQDKPKTDVVLEKVSVFSKGDEYKNYDPAKTFNEGKAKIAENNKAYIAKEEAERKKKEEEFKANQEKMVENLKAGMQKTESGLYYKITKTVAGGKAPKAGDNVSVHYAGKLIDGTEFDSSFKRNEPIEIPIGMGRVIKGWDEGILLLKEGETATLLIPPAMAYGERGAGGVIPPNAWLVFDVELVKVQ

>Chryseobacterium-pal

MLKKNFLIAAAAISLASCTPIYKKMNVDKETYEGLKDGLYANFQTSKGNMIVKFEDEKAPVTVANFVGLAEGKIENKSKAKGVPFYDGTKFHRVIKDFMIQGGDPLGTGMGDPGYKFDDEKNDLEHTGKGILSMANSGPNTNGSQFFITEVATPWLDGKHTIFGKVVKGENVIDDIANVEKGPQDKPKTDVILEKVSIFTKGDEYKNYDAAQIFSEGKGKIQEKNKAAMAKIAADKKKKEEEFAANQQRLVDDFKKDMQVTPSGLYYKITKTTTGEAPKAGDQVSVHYAGKLIDGTEFDSSFKRNQPIDIPIGVGQVIKGWDEGIMLLKEGETATLLIPSELGYGAAGAGGIIPPNAWLVFDVELVKVKGNATR

>Chryseobacterium-pip

MKLFNKNIILAAASISLMSCTPIYKKMNVDKETYEGLKDGLYANLQTSKGNMIVKFEDKKSPVTVANFVGLAEGKIDNKSKAKGVPFYDGTIFHRVIKDFMIQGGDPQGTGMGDPGYKFEDEKNDLQHTGKGILSMANSGPNTNGSQFFITEIATPWLDGRHTIFGKVVKGDDVIDAIANVEKGAQDKPKTDVVLEKVSVFSKGDEYKNYDAAKTFTEGKAKIQENNKAYLAKEEAEKKKKEEEFKANQEKLVENLKAGMQKTESGLYYKITKTTDGKAPKAGDNVSVHYAGKLVDGTEFDSSFKRNEPIDIPIGMGRVIKGWDEGILLLKEGETATLLIPSSMGYGERGAGGVIPPNAWLIFDVELVKVK

>Chryseobacterium-pis

MKIFNKNIILAAASVSLMSCTPIYKKMNVDKETYEGLNDGIYANLQTTKGNMIVKLEDKKAPVTVANFVGLAEGKIDNKAKAKGVPYYDGTIFHRVIKDFMIQGGDPQGTGAGDPGYKFEDEKNDLKHTGKGVLSMANSGPNTNGSQFFITEVATPWLDGRHTIFGKVVKGLETVDAIANVEKGAQDKPKTDIVLEKVGIFSKGDEYKNYDAAKTFNEGKAKIAANNKAMADKAEAEANKKMEDLKAGMQKTESGLYYKITKTTSGATPKVGDNVSVHYAGKLVDGTEFDSSFKRNEPIEIPIGMGRVIKGWDEGILLLKEGETATLLIPPAMGYGANGAGGVIPPNAWLVFDVELVKIK

>Chryseobacterium-pol

MKLFNKNIILAAASISLMSCTPIYKKMNVDKETYEGLKDGLYANLQTTKGNLIVKFEDKKSPVTVANFVGLAEGKIDNKSKAKGVPFYDGTIFHRVIKDFMIQGGDPKGTGAGDPGYKFEDERNDLHHTGKGILSMANSGPNTNGSQFFITEVATPWLDGKHTIFGAVVKGNDVIDAIANVDKGAQDKPKTDIVLEKVSIFSKGDEYKHYDAAKTFTEGKAKIAENNKAYVAKEEADRKKKEEEFKANQLKMVEDLQAGMQKTESGLYYKITKTTDGKAPKSGDNISVHYAGKLVDGTEFDSSFKRNEPIEIPIGMGRVIKGWDEGILLLKQGETATLLIPPTLGYGERGAGGVIPPNAWLVFDVELVKVP

>Chryseobacterium-sco

MKFLNKNIILAAASVSLLSCTPIYKKMNVDKETYEGLKDGLYANIQTTKGNLIVKFEDKKSPVTVANFVGLAEGKIDNKAKAKGVPFYDGTIFHRVIKDFMIQGGDPQGTGMGDPGYKFEDEKNDLKHTGKGILSMANSGPNTNGSQFFITEVATPWLDGRHTIFGEVVKGTETIDAIATVEKGAQDKPKTDIVLEKVSIFSKGDEYKGYDAAKTFNEGKGKIAANNKAMAEKAEAEAKKALEDLKAGMQVTESGLYYKITKKTEGKAAKAGDNVQVHYAGKLTNGTEFDSSFKRNEPLEFPVGTGRVIKGWDEGILLLKEGETATLLIPPAMGYGERGAGGVIPPNAWLIFDVELVKVP

>Chryseobacterium-shi

MKLFNKNIILAAASISLMSCTPIYKKMNVDKETYEGLNDGLYANLQTSKGNLIVQFEDKKAPVTVANFIGLAEGKIDNKAKAKGVPFYDGTIFHRVIKDFMIQGGDPQGTGMGDPGYKFEDEKNDLKHTGKGILSMANSGPNTNGSQFFITEVATPWLDGRHTIFGKVVKGNDVIDAIANVEKGAQDKPKTDIVLEKVSVFGKGDAYKNYDAAKTFTEGKAKIAENNKAYIAKEEADKKKKEEEFKANQEKMVEDLKAGMQKTESGLFYKITKTVEGAKAPKSGDNVSVHYAGKLIDGTEFDSSFKRNEPIEIPIGMGRVIKGWDEGILLLKEGETATLLIPPAMAYGERGAGGVIPPNAWLIFDVELVKVQ

>Chryseobacterium-sold

MKLFNKNIILAAASISLMSCTPIYKKMNVDKETYEGLNDGLYANLQTTKGNLIVKLEDKKAPVTVANFVGLAEGKIDNKAKAKGVPFYDGTIFHRVIKDFMIQGGDPQGTGMGDPGYKFEDEKNDLKHTGKGILSMANSGPNTNGSQFFITEVATPWLDGRHTIFGKVVKGDDVIDAIANVEKGAQDKPKTDIVLEKVSVFSKGDEYKNYDPAKTFTEGKAKIAENNKAFIAKEEAEKKKKEEEFAANQLKMVEDQKAGMQKTESGLYYKITKTTDGKAPKSGDNVSVHYAGKLIDGTKFDSSFDRNEPIDIPIGMGRVIKGWDEGILLLKEGETATLLIPPAMGYGERGAGGVIPPNAWLVFDVELVKVH

>Chryseobacterium-soli

MIKKLVIAFAAISLTSCTPIYKKMNVDKETYEGLKDGLYANFQTSKGNMIVQFEDKKAPVTVANFVGLAEGKIDNKSKGKGVPFYDGTIFHRVIKDFMIQGGDPKGTGMGDPGYKFDDEKNDLKHTGKGILSMANSGPNTNGSQFFITEIATPWLDGKHTVFGKVINGIEVIDSIANVEKGAQDKPKTDVVLEKVSVFTKGDEYKNYDPAKIFSEGKGKIKENNKAILEKLEAEKKKKAEEFAANQQKMVDDLKAGMQVTPSGLYYKITESTDGAKPNVGDEVAVHYAGKLIDGTEFDSSFKRNEPIVIPIGVGQVIKGWDEGILLMKEGESATLLIPSELGYGARGAGGVIPPNAWLIFDVQLVDIKSAK

>Chryseobacterium-sp

MKLFNKNIILAAASVSLLSCTPIYKKMNVDKETYEGLKDGLYANLQTTKGNLIVQFEDKKSPVTVANFVGLAEGKIDNKAKAKGVPFYDGTIFHRVIKDFMIQGGDPQGTGMGDPGYKFEDEKNDLKHTGKGILSMANSGPNTNGSQFFITEVATPWLDGRHTIFGKVVKGDDVIDAIANVEKGAQDKPKTDVVLEKVSIFSKGDEYKGYDAAKTFNEGKAKIAANNKAMAEKAEAEAKKALENLKAGMQVTESGLYYKITKKTEGKAPKAGDNIQVHYAGKLTNGTEFDSSFKRGEPLEFPVGTGRVIKGWDEGILLLKEGETATLLIPPAMGYGERGAGGVIPPNAWLIFDVELVKVP

>Chryseobacterium-tae

MKLFNKNIILAAASISLMSCTPIYKKMNVDKETYEGLNDGLYANLQTTKGNMIVKFEDKKAPVTVANFIGLAEGKIDNKAKAKGVPYYDGTIFHRVIKDFMIQGGDPQGTGMGDPGYKFEDERNDLKHTGKGILSMANSGPNTNGSQFFITEVATPWLDGRHTIFGKVVKGEDVIDAIANVEKGPQDKPKTDIVLEKVSVFSKGDEYKNYDAAKTFNEGKAKIAENNKAYLAKEEAEKKKKEEEFKANQEKMVEDLKAGMQKTESGLYYKITKTTDGKAPKSGDNVSVHYAGKLVDGTEFDSSFKRNEPIEIPIGMGRVIKGWDEGILLLKEGETATLLIPPAMGYGERGAGGVIPPNAWLIFDVELVKVK

>Chryseobacterium-taih

MKFLNKNIIFAAAGISLLSCTPIYKKMNVDKETYAGLNDGLYANLQTTKGNMIVKFEDKKSPVTVANFIGLAEGKIDNKAKAKGVPFYDGTIFHRVIKDFMIQGGDPKGTGMGDPGYRFDDEKNDLKHTGKGILSMANSGPNTNGSQFFITEVATPWLDGRHTIFGKVVKGEEVIDAIANVEKGAQDKPKTDIILEKVSVFGKGDEYKNYDAAKIFSEGKEKIAANNKAMEEKAEAEAKKALEDLKAGMQVTESGLYYKITKKTDGKAAKAGDNVQVHYAGKLTNGTEFDSSFKRNEPLEFPVGTGRVIKGWDEGILLLKEGETATLLIPPAMAYGERGAGGVIPPNAWLIFDVELVKVP

>Chryseobacterium-taiw

MKLFNKNIILAAASISLMSCTPIYKKMNVDKETYEGLNDGLYANLQTTKGNMILKFEDKKAPVTVANFIGLAEGKIDNKAKAKGVPYYDGTIFHRVIKDFMIQGGDPQGTGMGDPGYKFEDERNDLKHTGKGVLSMANSGPNTNGSQFFITEVATPWLDGRHTIFGKIVKGEEVIDAIANVEKGPQDKPKTDIVLEKVSIFSKGDEYKNYDAAKTFNEGKAKIAEKNKEFVAKEEADKKRKEEEFKANQLKMVEDLKAGMQKTESGLYYKITKTADGKAPKVGDNVSVHYAGKLVDGTEFDSSFKRNEPIEIPIGMGRVIKGWDEGIILLKEGETATLLIPPAMGYGERGAGGVIPPNAWLIFDVELVKVK

>Chryseobacterium-ten

MKKIIALSITLLTLLNCKTLEIDKEVYKGLPDGLYGNFVTSKGEILVKFEDEKSPVTVANFVGLAEGKIENKAKKKGEPFYDGTIFHRVIKDFMIQGGDPQGTGMGDPGYKFGDERNDLQHTGKGILSMANSGPNTNGSQFFITEIATPWLDGKHTIFGKVVGGEATIDSIANVEKGPQDKPKTDIVLTKVAVFAKGDAYKHYDAAKIFAEGKAKIEENNKAYLAKAEAEKAKKLAEFAANQEKLVNEMKAGMQSTESGLYYKITKTTTGANPTPGQTVAVHYAGKLINGEEFDNSFKRNAPIDIPIGVGQVIKGWDEGILLLKEGEAATLLIPPALGYGERGAGGVIPPNSWLVFDVELVKIEK

>Chryseobacterium-tre

MIKKLFIAFAAISLTSCTPIYKKMNVDKETYEGLKDGLYANFQTSKGNMIVQFEDKKAPVTVANFVGLAEGKIDNKAKGKGVPFYDGTIFHRVIKDFMIQGGDPKGTGMGDPGYKFDDEKNDLKHTGKGILSMANSGPNTSGSQFFITEIATPWLDGKHTVFGKVINGIEVIDSIANVEKGAQDKPKTDVVLEKVSVFTKGDEYKNYDPAKIFSEGKGKIKENNKAILEKLEAEKKKKEEEFAANQQKMVDDLKAGMQVTPSGLYYKITESTDGAKPNVGDEVAVHYAGKLIDGTEFDSSFKRNEPIVIPIGVGQVIKGWDEGILLMKEGESATLLIPSELGYGARGAGGVIPPNAWLIFDVQLVDIKSAK

>Chryseobacterium-ure

MKLFNKNIILAAASISLMSCTPIYKKMNVDKETYEGLNDGLYANLQTTKGNMIVKFEDKKAPVTVANFIGLAEGKIDNKAKAKGVPYYDGTIFHRVIKDFMIQGGDPQGTGMGDPGYKFEDERNDLKHTGKGILSMANSGPNTNGSQFFITEVATPWLDGRHTIFGKVVKGIDVIDTIANVEKGAQDKPKTDIVLEKVSIFGKGDEYKNYDAAKTFNEGKAKIAENNKAFIAKEEAEKKKREEEFKANQEKLVESLKAGMQKTESGLYYKITKTAEGKAPKAGDNVSVHYAGKLVDGTEFDSSFKRNEPIEIPIGMGRVIKGWDEGILLLKEGETATLLIPPAMGYGERGAGGVIPPNSWLIFDVELVKVK

>Chryseobacterium-wan

MKLFNKNIILAAASISLMSCTPIYKKMNVDKETYEGLNDGLYANLQTTKGNMIVKFEDKKAPVTVANFIGLAEGKIDNKAKAKGVPYYDGTIFHRVIKDFMIQGGDPQGTGMGDPGYKFEDEKNDLHHTGKGVLSMANSGPNTNGSQFFITEVATPWLDGRHTIFGKVVKGEDVIDAIANVEKGAQDKPKTDIVLEKVSIFSKGDEYKNYDAAKTFTEGKAKIAENNKAYLAKEEAEKKKKEEEFKANQEKMVENLKAGMQKTESGLYYKITKTTDGKAPKAGDNVSVHYAGKLVDGTEFDSSFKRNEPIEIPIGMGRVIKGWDEGILLLKEGETATLLIPPAMGYGERGAGGVIPPNAWLIFDVELVKVK

>Cloacibacterium-nor

MKKIFLLTIAIITLINCTPIYKKMNIDKDFYNGLQDGVYAKMETSKGELIIQFFDQDAPVTVANFVGLAQGTIENKAKAKGVPYYDGIVFHRVIKNFMIQGGDPQGTGMGDPGYKFDDEKNDLKHEGKGYLSMANSGPNTNGSQFFITEVPTPWLDGRHTIFGKVIKGEDVIDTIANSETGAQDRPKEEIKIVKVTVFTKGDAYEKYDAAKIFNEGKAKIQENNKAYIAKQEAEAAKKLEDLKAGMTKTASGLLYKITKTNPEGKAPKAGDMVSVHYAGKLTNGQEFDNSFKRGEPIEIPIGVGQVIKGWDEGIQLLKEGEAATLLIPSELGYGTRGAGGVIPPNAWLIFDVELVKVK

>Croceibacter-atl

MNNGLYAKLETSKGDILIQLEFQKTPGTVANFVALAEGKQENSAKDLGTPYYDGLKFHRVIADFMIQGGDPQGTGAGGPGYNFDDEFHPELKHNKPGTLSMANAGPGTNGSQFFITHGETAWLDNKHTVFGYVVEGQDVVNKIKQGDLINHVEIIREGEDAEAFDAVETFKNFNESKAQRIADAKKREEEALAKATEGFTKTNSGLYYNITKKGDGKAAEKGKTVSVHYKGMLMDGTVFDSSFKRNEPIDFPLGVGQVIAGWDEGIQLLNVGDQATLIIPSDLAYGERGAGGVIPGGATLKFDVELVNVK

>Crocinitomix-cat

MKKLLLSALLFASVGLFAQKDLEDGLYAKMTTTKGEIMLALEFEKTPITVANFVALAEGNLEYDTIEISKPYYDGVTFHRVIANFMIQGGDPTATGSGGPGYSFPDEFDTSLTHKGPGILSMANAGPGTNGSQFFITHKATPHLDGKHSVFGHVVTGQEVVDKIEQGDAMEKVEIIRVGKAAKKFKASKTFSTKVTELKEAEAKILAERNGIFYEEMIKKFPEAKQTESGLMYQIIKKGDGVYPAKNKTAEVHYTGTFLDGNKFDSSVDRGQTFQCQVGQGRVIKGWDEGIPMCDVGGQIKLIIPYWLAYGENGRSSIPPKSTLIFDIEVFGVK

>Cruoricaptor-ign

MLSAAAAAILTSCTPIYKKMNVDKQTYEGLNDGLYANLQTSQGNMLVKFEDEKSPVTVANFIGLAEGKIDNNKKPKGVPFYDGTIFHRVIQDFMIQGGDAQGTGMGDPGYKFEDEKNDLKHTGKGILSMANSGPNTNGSQFFITQVPTPWLDGKHTVFGKVVEGEEVIDKIAAVEKGPQDKPKTDVVLEKVSVFSKGNQYKDYDATKVFNEGKGKIQENNRDFLRKQEAEATKQLEELKQGMETTASGLMYKITKKGSGPKVEQGQMVAVHYAGRLTNGMEFDNSFKRGEPIEFPVGTGRVIRGWDEGLLLLNVGDEATLLIPSNLGYGERGAGGIIPPNAWLIFDVQVMGAK

>Cryomorphaceae-bac

MNNGIYAKFTTPKGEILVQLEYEKTPGTVGNFVALAEGNLENKIKPQGTPYYNGLKFHRVISDFMIQGGCPSGTGSGNPGYKFEDEFHPELKHNKPGKLAMANSGPATNGSQFYITHVPTPWLDGKHTVFGSVIEGQDVVDAVEQGDEMSVEILRIGEETQNFNAVEAFRNFEGSREKREEELKRKQKELLETVAADFEETVSGLRYQILQKGNGKQAEKGSKVSVHYKGQLLDGTVFDSSYERNEPIDFTVGFGQVIEGWDEGIQFLKVGDKARFVIPSDLAYGESGAGGVIPKNATLIFDVELMKIK

>Desulfobacterales-bac

MKDGLYAKFDTTKGEIICALEYTKTPLTVTNFVGLAEGTKDLGGGAKMKGDKFYDGMKFHRVIPDFMIQGGCPLGTGTGGPGYTFPDEIDPSLTHKGPGILSMANAGPGTNGSQFFITHVPTPWLDGKHTVFGHVVAGQDVVNKIATGDVINSVEIVRVGAAAEAFKADQATFDALLAGFEKNQRDKERGAVELEKKQIAETWPGAITTPTGLKYVVVAEGQGEPPQKGAMVTVHYTGKLLSGKKFDSSYDRGQPIDFPVGRGQVIKGWDEALLSMKKGEKRVLIIPSQLGYGPAGRGPIPPNATMVFDVELVDFK

>Desulfobulbus-pro

MTRLPMLLLLSALLFCSGQPAFSAEEKTMKDGLYAKITTAKGDILLKLFYTKTPLTVINFVGLAEGTLHLGGSTKPTGTPFYNGLTFHRVIANFMIQGGCPLGTGTGGPGYTFPDEFDASLRHDGPGVLSMANAGPGTNGSQFFITHLATPHLDDKHTVFGRVVEGQEVVNKIDKGDAIKAITIIRVGKEAEAFKTDQAAFDAALKAIADREANARKAQQEKITKMIKEQWPKAVRSDSGLYSQVEQKGEGNPPPAGTVIKAHYTGRLLLGNRKFDSSYDRGEPIAFPVGTGRVIRGWDEALSQMTKGEKRTLIIPPELAYGERGAGGVIPPNAWLVFDVELVGF

>Desulfocapsa-sul

MHKIGTLLIATLLLLFTLIGDNLMAENKLADGMYAKFVTNKGEIICALEYNKTPITVANFVGLAEGTKELGGGAGKAGVRFYDGLTFHRVIGDFMIQGGCPLGTGTGGPGYTFPDEIDPTLKHSSPGILSMANAGPGTNGSQFFITHVPTPHLDGKHTVFGHVVSGMDVVNKIDDKIETIEIIRVGADAEAFKSDQAAFDALLASQEARAKEKELAAMEEAMELINGKYPDAITTASGLKYVVVAEGEGNTPAAGAMVKVHYTGTLLDGSKFDSSVDRGTPIEFPVGQGRVIKGWDEALLTMKKGEKRVLIIPANLGYGPSGRGPIPPNATMIFDVELIDF

>Desulfotalea-psy

MHRVSRQLLITLIFLLLFGGEGMANSNLKDGLYAQFNTSKGVIICSLEFEKTPLTVANFVGLAEGTKELGGGSGKKGARFYDGLTFHRVIPNFMIQGGCPLGTGTGGPGYNFPDEFDSTLTHDAPGVLSMANAGPGTNGSQFFITHVATPWLDGKHTVFGRVVEGQDVVDKIEGKDALESITIIRVGAKAEAFKSDQAAFDDLLGSIDSRQKEKELQSMEASLNQIKEQWPAAITTDSGLQYVVVEAGEGEATPNVGDVVTVHYTGKLLDGTKFDSSVDRGQPIDFPVGRGQVISGWDEALLSMTKGEKRVLIIPAKLGYGAAGRGPIPANATMVFDVELVDFK

>Dokdonia-sp-1

MKDGIYAKIHTPKGEIILKLEHEKTPGTVGNFVALAEGNLENSQKPQGTPYYDGLKFHRVIPDFMIQGGCPLGTGTGDGGYKFDDEIHPDLKHDAPGILSMANAGPGTNGTQFFITHIPTDWLDGKHTVFGNVVEGQEVVNAVAQGDEITKIEIIRVGAEAEAWNAVEAFRTFEGSRTKRVEEEKAAQAAEIEALATGFETTASGLRYQIIQKGTGAKAEKGKTVSVHYKGALPDGTVFDSSFKRNQPIDFQLGVGQVIPGWDEGISLLNVGDKARLVIPSDLGYGSAGAGGVIPPNATLVFDVELVAVK

>Dokdonia-sp-2

MQDGIYAKIHTPKGEITLKLEHEKTPGTVGNFVALAEGNLENSQKPQGTPYYDGLKFHRVIPDFMIQGGCPLGTGTGDGGYKFDDEIHPDLKHDAPGKLSMANAGPGTNGTQFFITHIPTDWLDGKHTVFGNVVEGQDVVNSVAQGDEMEKIEIIRVGAEAEAWNAVEAFRTFEGSRAKRIEEERAAQAAEIEALATGFDTTDSGLRYQIIQKGDGVKAEKGKTVSVHYKGALPDGTVFDSSFKRNQPIDFQLGVGQVIPGWDEGIALLNVGDKARLVIPSDLAYGSAGAGGVIPPNATLVFDVELVAVK

>Elizabethkingia-ano

MKKVVFLCTVLLTLLNCKTLDLKEINLDKAAYEQLPEGLYGNLKTTKGDILVKFNDKESPVTVANFVGLAEGKIENSAKKKGEPFYNGTIFHRVIKDFMIQGGDPKGTGMGDPGYKFDDEKNDLKHTGKGILSMANSGPNTNGSQFFITEVATPWLDGRHTIFGKVVKGEQVIDDVANVEKGAQDKPKTDIVLEKVTIFTKGDAYKHYDAAKLFNEGKSKIAENNKVFVQKKEEEAKKKLEELKSGMTTTASGLMYKITKTTDGAQPVAGNTVSVHYTGKLTNGQVFDSSISRNEPIEFPVGTGRVIKGWDEGILLLKEGEEATFLIPPDLGYGARGAGGVIPPNAWLIFEVKLLKAKA

>Elizabethkingia-men

MDLKEINLDKAAYEQLPEGLYGNLKTSKGDILVKFNDKESPVTVANFVGLAEGKIENKSKKKGEPFYNGTIFHRVIKDFMIQGGDPKGTGMGDPGYKFDDEKNDLKHTGKGILSMANSGPNTNGSQFFITEVATPWLDGRHTIFGKVVKGENVIDDVANVEKGPQDKPKADIVLEKVTIFTKGDAYKHYDAAKVFNEGKSKIAESNKTFVQKKEEEAKKQLEELKNGMTTTASGLMYKITKTTEGEKPVAGNTVSVHYTGKLTNGQVFDSSISRNEPIEFPVGTGRVIKGWDEGILLLKEGEEATFLIPPDLGYGARGAGGVIPPNAWLIFEVKLVKAKA

>Elizabethkingia-mir

MPVKVIINAYLNKYRHIHYKNAYFSATKKTQFILYRSFMKKVVFLCTVLLTLLNCKTLDLKEINLDKAAYEQLPEGLYGNLKTTKGDILVKFNDKESPVTVANFVGLAEGKIENSAKKKGEPFYNGTIFHRVIKDFMIQGGDPKGTGMGDPGYKFDDEKNDLKHTGKGILSMANSGPNTNGSQFFITEVATPWLDGRHTIFGKVVKGEQVIDDVANVEKGAQDKPKTDIVLEKVTIFTKGDAYKHYDAAKLFNEGKSKIAENNKVFVQKKEEEAKKKLEELKSGMITTASGLMYKITKTTDGAQPVAGNTVSVHYTGKLTSGQVFDSSISRNEPIEFPVGTGRVIKGWDEGILLLKEGEEATFLIPPDLGYGARGAGGVIPPNAWLIFEVKLVKAKA

>Eubacterium-uni

MQDGIYVKITTEKGEILGQLHYKRTPGTVANYVALAEGNLENEAKPQGTPYYDGLTFHRVIPDFMIQGGDPAGTGAGGPGYKFDDEFHPELKHDKPGIFSMANAGPGTNGSQFFITHVPTDWLDGKHTVFGEVIEGMDVVNSVAQGHTIEKMEIIRVGEEAKAFNAVETFRKFNGAKAEREAAAKKAQEEAMGDLVAGFDKTESGLHYKIIQKGDGPKPTSGNTVAVHYKGMLADGTTFDSSYKRGNPIEFPVGMGHVIAGWDEGILMLNKGDKARFVIPSDLGYGAQGAGGVIPPNATLVFDVELMDIKG

>Eudoraea-adr

MQDGIYAKFNTSKGEITVKLTHDKTPGTVGNFVALAEGTMENSAKPKNTPYYDGLSSFHRVIPDFMIQGGCPQGTGTGDPGYKFDDEFHPDLKHSGPGVLSMANAGPGTNGSQFFITHVATPWLDNKHTVFGKVVEGQNVVDEIDQGDLINDLEILRIGNDAESWNAVEAFTTFESSRELRLQEEKNKAEAELDKVAAGFDKTNTGLRYKILKKGSGNQAQKGNTVSVHYEGSLTNGQIFDSSYQRNQPIDFQLGIGQVIPGWDEGISLLKVGDKARFVIPSELGYGSAGAGGVIPPNATLIFDVELMGVK

>Flagellimonas-sp

MQEGIYAKFNTTKGEILVKLTHDKTPGTVGNFVALAEGNMENAVKPQGKPYYDGLKFHRVIPDFMIQGGCPQGTGTGDAGYKFDDEFHPELTHEGPGVLSMANAGPGTNGSQFFITHVPTPWLDNKHTVFGHVESGQEVVDTIAQNDAIETLEIVRVGAEAEGWNAIEAFRTFEGSREKRIAEQKALAEAEMEKLAAGFNATESGLRYKMIQQGSGTKAEKGKTVSVHYEGSLPNGQVFDSSYSRNQPIDFTLGVGQVISGWDEGIALLKVGDKARFVIPSHLAYGSAGAGGVIPPDATLIFDVELMQVK

>Flavobacterium-aki

MENGIYAKFNTNKGTILVKLTHDKTPGTVGNFVGLAEGNLENHEKPQGKPYYDGLAFHRVIPDFMIQGGCPQGRGTGGPGYNFDDEFHPELKHDAPGVLSMANAGPGTNGSQFFITHVPTPWLDGKHTVFGHVVEGQDVVDAIGQNDKIETLEIIREGEEAKNWNAIEAFRTFEGSREKRLAEQKRQAEEAMEKLAAGFDKTESGLRYKIIQKGNGKKAEKGKKVSVHYKGALENGMEFDSSYKRKQPIDFTVGVGQVIEGWDEGILLLQVGDKARFVIPSDLGYGSRGAGGVIPPDATLIFDVELMDVK

>Flavobacterium-ant

MENGIYAKFNTPKGSILVKLTHDLTPGTVGNFVALAEGNLENTARKQGEPFYNGLKFHRVIPDFMIQGGCPQGQGTGNPGYKFDDEFHPSLKHDRPGVLAMANSGPGTNGSQFYITHIPTDWLDNKHTVFGHVVEGQDTVDTVAQGDILESVEIIRVGEEAQKWNAVEAFRVFEGSRAKREAAEREAGESAMEAIAAGFEKTASGLRYKMINKGTGKRAEAGKTVSVHYTGQLPDGKVFDSSYPRKKPIEFPLGKGHVIAGWDEGIALLSVGDKARFAIPSHLAYGSAGAGGAIPPNANLIFDVELMDVK

>Flavobacterium-aqu

MKFKFLFLFCLAVVNIQAQTKKPVAKPKAPVTKAIAKTDPNDGIFATISTTKGDIVVSLEYVKTPVTVANFISLAEGNNPNVKVERLKGKPFYDGLKFHRVINDFMIQGGDPDGNGSGGPGYAFKDEFVEELKFEKGGVLAMANSGPATNGSQFFITHKETPWLNGKHTIFGHVVSGMDNVNKIVQDDIMTKITITRKGDAAKKFNAVKVLADDVKKQEAKKAESQKVVKDKAAYFAANKAKATTTASGLKYVITKKGTGVKGAEGSAIYFHYAGYFEDGNLFDSSMASVAKAYGKYDANRDAQKGYQAFPFTVGKKDGMIPGFIEALDLMTDGEKAIFFLPSNLAYGEKGAGGVIPPNSTLIFEIETYQNQPVK

>Flavobacteriaceae-bac

MNNGIYAKFITPKGKILVQLEHEKTPGTVGNFVALAEGNLENKIKPQGTPYYNGLKFHRVISDFMIQGGCPSGTGSGNPGYKFDDEFHPELKHNKPGKLAMANSGPATNGSQFYITHVPTPWLDGKHTVFGSVIEGQDVVDAVEQGDEMSVEILRIGDEAQNFNAVEAFRNFEGSRARREVEAKRKQKELLDTVAAGFDETASGLRYQILQKGDGKQAEQGSKVSVHYKGQLLDGTVFDSSYKRKEPIDFTLGIGQVIEGWDEGIQLLKVGDKARFVIPSDLAYGERGAGGVIPPNATLIFDVELMKVK

>Flavobacteria-BAL

MKNIFKIVVIVLSSISFAVAQNKSEEGIFAEFNTTKGKIVVLLEYKKTPITVSNFISLAEGNNIQVSEKLKGKPYYNGLKFHRVIADFMIQGGCPKGDGTGDPGYKFDDEFVADLKHSEKGILSMANAGPATNGSQFFITHRATPHLDGKHTVFGHVVSGIEVVDKIAKDDVITSVKIIRVGKEAKKFNAQKTFSDYFLNKAAADKLKAEKLKEAKEKAIAEFANAGTTASGLKYIVLQEGTGNKPVASSNVKVHYTGMFLDGKVFDSSVQRGETIDFGLNQVIKGWTEGVQLMPEGSKYKFYIPSNLAYGERGAGGVIPPNTDLIFEIELIKINQ

>Flavobacterium-BBF

MIAYLCSLKKEDMQEGIYAKFNTTKGSILIQLHHDKTPGTVGNFVALAEGNLENDAKPQGTPYYDGLTFHRVIPDFMIQGGDPQGSGAGGPGYKFDDEFHPELRHDGPGVLSMANAGPGTNGSQFFITHVETAWLDDKHTVFGKVVEGQDVVDAIAQGDSIETVEIIREGNAAQSFNAVEEFRQFEGAAKLREEQARKQAEQDLDEIAAGFDKTDSGLRYKIIQEGNGAKAESGKTVSVHYKGMLPNGKVFDSSFERKQPIDFQLGAGQVIAGWDEGIALLKVGDKARLVIPSHIGYGSAGAGGVIPPNATLVFDVELVGIK

>Flavobacterium-bei

MENGIYAKFNTPKGSILVKLTHDKTPGTVGNFVGLAEGNLENDAKPQGKPYYNGLKFHRVIPDFMIQGGCPVGTGVGGPGYQFDDEFHPELTHSGPGVLSMANAGPGTNGSQFFITHVETPWLDGKHTVFGHVVEGQDVVDAVAQGDEIESIEIIREGDEAKNWNAVEAFRVFEGAREKRIEEAKQKAEAEMEKLAAGFDKTESGLRYKIIQKGNGKKAEKGKTVSVHYTGALDDGRVFDSSYKRKDPIAFTLGIGQVISGWDEGIALLQVGDKARFVIPSHLGYGSNGAGGVIPPDATLVFDVELMDVK

>Flavobacterium-bra

MENGIYAKFNTSKGSILVKLTHDLTPGTVGNFVALAEGNLENSIKPQGTKYYDGLKFHRVIPDFMIQGGCPQGTGTGGPGYKFDDEFHPTLKHDQPGVLSMANAGPGTNGSQFFITHVATPWLDNKHTVFGHVVEGQDIVDAIAQGDVLEQLEIIRQGDDAQKWNAVEAFRVFEGSRAKREAQLRAEAEAKMEKLAAGFEKTASGLRYQMIQKGSGKKAENGKTVSVHYAGQLEDGKVFDSSYTRKKPIEFPLGRGHVIEGWDEGIALLQVGDKARFVIPSHLGYGANGAGGVIPPNATLIFDVELMDVK

>Flavobacterium-cau

MQDGIYAKFNTSKGVILIKLTHDKTPGTVGNFVGLAEGNLENDIKPQGKPYYDGLKFHRVIPDFMIQGGCPLGTGTGDPGYKFDDEFHPELKHDKPGVLSMANAGPGTNGSQFFITHVPTDWLDNKHTVFGQVVEGQDIVNEIVQGDIIESIEIVRVGEEAEKWNAVEAFRTFEGSREKRIAEQKKMAEEALEKLAAGFDKTESGLRYKMIQKGNGKKAEKGKTVSVHYSGSLENGMVFDSSYKRKQPIEFTLGRGQVIEGWDEGIALLQVGDKARFVIPSYLGYGSRGAGGVIPPDATLIFDVELMDVK

>Flavobacterium-chu

MKFKFLFLFCLAVLNIQAQATKKPSAKSKTAITKPAATANPAEGIFATIATTKGDIVLTLEYVKAPVTVANFISLAEGKNPNVKVEKLKGKPFYDGLKFHRVINDFMIQGGDPDGNGSGGPGFSFKDEFVKELVFDKGGILAMANSGPATNGSQFFITHKETPWLNGKHTIFGHVVSGMDNVNKIVQDDIIKKITITRKGAAAKKFDAVKVLAEDVKKDEAKKLESQKVIKDKAAYFTSTKANAVTTASGLKYVITKKGTGVKGAEGSTIYFHYAGYFEDGNLFDSSMPNVEKAYGKYNANRDAQGGYKAFPFTVGKKDGMIPGFLEALDMMTDGDKAIFFLPSNLAYGEAGAGGVIPPNATLIFEIETYSKQP

>Flavobacterium-col

MQDGIYVKFNTSKGAILVKLTHDLTPGTVGNFVGLAEGNLENSVKSQGQKYYDGLKFHRVIPDFMIQGGCPLGTGTGDAGYKFDDEFHPSLKHDRPGVLSMANAGPGTNGSQFFITHVATPWLDGKHTVFGHVIEGQDVVDAVLQDDLIESVEIIRVGAEAEKWNAIEAFRTFEGSRVKRLEEAKRLAEEAVDKLSAGFERTESGLRYKIIQKGEGKKAEKGKTVSVHYQGSLENGQVFDSSYKRKQPIDFPLGKGYVIEGWDEGIALLQVGDKARFVIPSYLGYGDRGAGGVIPPNATLVFDVELMDVK

>Flavobacterium-dae

MKTKLLTLVAFFALFTLQAQETSKKITKKKTEKAAPIKEDGIYATINTNKGAIVLQLYFDKTPVTVANFVSLAQGKNEFVSDKKRKGKPFYDGLKFHRVISDFMIQGGDPMGNGSGDPGYAFKDEFLSDLKFDKGGILAMANSGPKTNGSQFFITHKETSWLNGKHTIFGHVVQGMDVVNSIAQDDVIKNIIITQKGSLAKKFKAEKVFANYYNNKAEEERKEAAAKAEAERIQKEKMAALESEKLAYFNTQKATATTTPSGLVYKIIQKGNGAKPAAGTTVYFHYSGFFENGKLFDSSREEIAKNYNQYNEMRKMQGGYNPFPFEIGKKDGMIPGFIEAINLLSFGDKIIAYIPSKLAYGESGAGGVIPPNATLIFEMEIFDKQK

>Flavobacterium-def

MENGIYAKFNTSKGSILVKLTHDLTPGTVGNFVALAEGNMENKVKPQGQKFYDGLNFHRVIADFMIQGGCPKGTGTGDPGYKFDDEFVPSLKHDRPGVLSMANSGPGTNGSQFFITHVPTPWLDGKHTVFGHVVEGQNIVDAVAQGDALESVEIIRVGEEAQKWNAIEAFISLKGARLKREAALKAESEAKMEQLAAGFDKTESGLRYKMIQKGEGKKAEAGKTVSVHYEGSLENGKVFDSSYPRKKPIEFKLGIGQVIEGWDEGIALLQVGDKARFVIPSDLAYGPSGAGGVIPPHATLIFDVELMDVK

>Flavobacterium-ens

MQDGIYAKFNTSKGVILVKLTHDKTPGTVGNFVGLAEGKLENDVKPQGKPYYDGLKFHRVIPDFMIQGGCPLGTGVGGPGYQFDDEFHPELKHDGPGVLSMANAGPGTNGSQFFITHIETAWLDNKHTVFGHVVEGQDIVDAIAQGDEIETLEIVRVGEEAQKWNAVEAFRTFEGSREKRLAEQKQLAEEALEKLAAGFQKTDSGLRYQIIQKGNGKKAEKGKMVSVHYQGALDNGMVFDSSYKRKQPIEFALGRGQVIEGWDEGIALLQVGDKARFVIPSYLAYGSRGAGGVIPPDATLVFDVELMDVK

>Flavobacterium-fil

MKKIIYVLIFTFSIVAQAQKENGIYAEINTIKGKILLKLEYEKTPITVANFIALAEGTNPDVAEKFKGKPYFNGLKFHRVIASFMIQGGDPDGNGSGGPGYKFFDEITDLKHDKPGILSMANAGPATNGSQFFITHVPTPHLDGKHTVFGHVIEGQEVVNAIAQNDVMDKITIIRVGEAAKKFDAPSVFKSRSAIEEAMKKKKEEDLIKEKQQFFDFVKKTYPKATILPSGLAYILEKEGTGNKAVAGNTVSVHYLGELADGKKFDSSYDRNQPIEFKLGQRMVIPGWEEGIALLNKGAKTKLIIPYWLAYGESGRPPVIPAKATLIFNTELVEIK

>Flavobacterium-fri-1

MKLKLLFLVCIGMLNAQAQSIKKTVPAKKISTTTKTVVKKPIVVEGIFATISTNKGDITVQLDYQKAPITVANFVALAEGKNTFVTDEKLKGKPFFDGLKFHRVINDFMIQTGDPTGTGAGGTGFAFKDEFTDVKFDKGGILAMANSGPATNSSQFFITHKETPWLNGKHTIFGHVTEGIDIVNSVVQGDVITKITISRKGASAKYFDAPKVFANYYTNKEADTKKQAAIEEETKRNLAPKQTVVIAAKKAFFDVTKTTATTTESGLTYKIIQKGSGVKPVDGSTFYFHYAGYFEDGNLFDSSYEDVNKAYGKYDERRAAQNGYQAFPFEAGKKDGMIPGFIEGLSLMSYGDKTLLFIPANLAYGERGAGGVIPPNTNLIFELEMFEKPTPPKK

>Flavobacterium-fri-2

MENGIYAKFNTSKGAILVKLEHELTPGTVGNFVALAEGNMENKVKPQGTKFYDGLSFHRVIPDFMIQGGCPQGTGTGDPGYKFDDEFHPSLKHDKPGILAMANSGPGSNGSQFYITHVPTSWLDGKHTVFGHVVEGQDIVDAVAQGDKLETLEIVRVGDEAKNWNAIEAFITFKGARNKRDAALKADAEAAMEKLAAGFEKTESGLRYQFIQRGDGKQAQAGKTVSVHYEGSLENGKVFDSSYPRKKPIEFKLGQGQVIEGWDEGIALLKVGDKARFVIPSDLGYGSRGAGGAIPPNATLIFDVELMDVK

>Flavobacterium-fry

MENGIYAKFNTTKGAILVKLTHDLTPGTVGNFVGLAEGNVENKVKPQGVKFYDGLKFHRVIPNFMIQGGCPQGTGTGDPGYKFDDEFHPSLKHNRPGVLAMANSGPGTNGSQFYITHIPTDWLDNKHTVFGHVVEGQDIVDAVEQGDVLESLEIIREGEEAKNWNAIEAFITFKGSRNKRDAAVKAEVEAAMEKLAAGFEKTESGLRYQFIQRGEGKKAESGKTVAVHYEGSLENGKVFDSSYPRKKPIEFKLGQGQVIEGWDEGIALLRVGDKARFVIPSDLGYGSRGAGGAIPPNATLIFDVELMEVK

>Flavobacterium-gil

MKSKITLLLFLGLFNLYAQPKKVGPAKKPATTAVKTTTPTEGIFATIATNKGNITLELFYKKTPVTVANFISLAEGKNPFVTIEKLKGKPFFDGLKFHRVISDFMIQGGDPNGNGSGTPGYSFKDEFTDSKFDKGGILAMANSGPGTNGSQFFITHKETSWLNGKHTIFGQVTQGMNVVNTIAQDDVITKITITRKGEAAKKFDAVKVFSDYYSNKATDEKLQKEKEEKERAAKNAIALKEFANGQTTASGLKYIVLKEGTGATPKIDSNVKVHYTASFIDGTVFDSSIKRGEPIDFNLNQVIPGWTEGVQLMKEGAKYKFYVPYTLAYGERGYPGAIPPKSDLIFEVELIKINQ

>Flavobacterium-hao

MQDGIYAKFNTPKGSILVKLTHDKTPGTVGNFVGLAEGQLENSARPMGKPYYDGLKFHRVIPDFMIQGGCPQGQGTGGPGYNFDDEFHPELRHDGPGVLSMANAGPGTNGSQFFITHVETGWLDGKHTVFGHVVEGQDVVDAIAQGDAIESIEIVRVGDEAKNWNAIEAFRTFEGSREKRIAEQKRLAEEALEKLAAGFQKTESGLRYQIIQKGSGKQAEKGKKVSVHYQGALENGQVFDSSYKRKQPIDFTLGVGQVIEGWDEGIALLKVGDKARFVIPSYLGYGSRGAGGVIPPNATLVFDVELMDVK

>Flavobacterium-hib-1

MKFKFLFLFCLAVVNIQAQTTKKPVAKPKAPATKAVTKSTSQADPNDGIFATISTTKGDIVLSLEYVKTPVTAANFISLAEGKNPNVKVERLKGKPFYDGLKFHRVINDFMIQGGDPDGNGSGGPGYSFKDEFVEELKFEKGGVLAMANSGPATNGSQFFITHKETPWLNGKHTIFGHVVSGMDNVNKIVQDDIMTKITITRKGAAAKKFDAVKVLADDAKKQDAKKAEAQKVITQKAAYFAATKAKATTTASGLKYVITQKGSGVKGAEGSNIYFHYAGYFEDGKLFDSSMANVAKAYGQYDANRDAQKGYQAFSFAVGKKDGMIPGFLEALDMMTDGEKAIFFLPSNLAYGEKGAGNVIPPKTTLVFEIETYKEQPTK

>Flavobacterium-hib-2

MENGIYAKFNTSKGSILVKLTHDLTPGTVGNFVALAEGNMENKVKPQGQKFYDGLTFHRVIPDFMIQGGCPKGTGTGDPGYKFDDEFHPSLKHDRPGVLAMANSGPASNGSQFYITHVPTSWLDNKHTVFGHVIEGQDIVDAVAQGDALESVEILRVGEEAEKWNAIEAFVGLKGARLKRDAALKAESEAKMEQLAAGFDKTDSGLRYKMINKGEGKKAEAGKTVAVHYEGSLENGKVFDSSYPRKKPIEFRLGQGQVIEGWDEGIALLQVGDKARFVIPSDLAYGAAGAGGVIPPNATLIFDVELMEVK

>Flavobacterium-hyd-1

MKLKILFLFCLGVLNLQAQTKKPVPGKKTTITKVETKPSVNEGIFATIATKKGNIVLQLEYIKAPITVANFVSLAEGTNPYVTIDKLKGKPFYDGLKFHRVINDFMIQGGDPDGNGSGGPGYAFKDEFDSSLRFDKGGILAMANSGPTTNGSQFFITHKETPWLNDKHSIFGHVVEGMDVVNLILQDDVMITVKITRKGAMAKKFDAPKVFAAYYDNKAEDAKKQALIDAEKAKQAQAAALEQERIYKEKYATVIAAKKAYFETAKATATTTPSGLSYKIVQKGTGVKPVDGSTFYFHYAGYFEDGTLFDSSFEEIAKTYGKHDTNRAAQGGYQAFPFQAGKKDGMIPGFLEGLDLMTYGEKAVFFLPSKLAYGERGAGGVIPPNTTLIFELEIYKDQPTPKK

>Flavobacterium-hyd-2

MENGIYAKFNTSKGSILVKLAHDLTPGTVGNFVGLAEGNLENKIKPQGNKFYDGLKFHRVIPDFMIQGGCPQGTGTGGPGYKFDDEFHPSLKHDRPGVLAMANSGPATNGSQFYITHVPTSWLDGKHTVFGHVVEGQDIVDAIAQGDDLTNVEIVRVGEEAEKWNAIEAFIAFKGERNKRDAAMKAEAEAAIEKLAAGFEKTESGLRYQFIQRGDGKKAENGKTVAVHYEGSLDNGKVFDSSYPRKKPIEFRLGQGQVIEGWDEGIALLKVGDKARFVIPSHLGYGPSGAGGVIPPNANLIFDVELMDVK

>Flavobacterium-ind

MQDGIYAKFNTPKGSILVKLTHDKTPGTVGNFVGLAEGQLDNEAKPMGKPYYDGLKFHRVIPDFMIQGGCPQGTGVGGPGYQFDDEFHPELRHDKPGVLSMANAGPGTNGSQFFITHVETPWLDGKHTVFGHVVEGQAVVDAVAQGDTIESIEIVRVGAEAEKWNAIEAFRTFEGSREQRLAEEKRLAEEALEKLAAGFQKTESGLRYQIIQKGSGKQAEKGKKVSVHYQGALENGMVFDSSYKRKQPIDFTLGVGQVIEGWDEGIALLQVGDKARFVIPSYLGYGSRGAGGVIPPNATLVFDVELMDVK

>Flavobacterium-joh-1

MKKIFLLLLAVSSFYSCKNEHSNLPDGLYADIETNKGHIIVELDYKKAPITVANFVTLAEGKNEFVTKDYLKSKPFYNGLKFHRVIENFMIQSGDPEGTGSGDAGYKFKDEFSDLKFDKAGILAMANNGPGTNSSQFFITHVDTPWLDNKHTIFGHVVDEKGQEVVNKVVQGDTIVSVSIIRNGEAAKKFDAVKVFHDYFAEAAKEKSKFAGAQKEKVDYYASIKPKATKTSSGLEYVITEKGSGKKPATGAQVYIHYAGFLEDGTLFDSSIEDVNKTFGKFDAARAEAKGYQPIPFQAGRKDGLIPGFIEGIEKLSFGDKAVLFIPSHLAYGATGAGGVIPPNANIIFEVQLLEKP

>Flavobacterium-joh-2

MKFKFLFLFCLAVLNIQAQTKKPAAKTAPAKTVAATDPNDGIFATISTTKGDIVLSLEYVKAPVTVANFITLAEGTNPNVKASLKGKPFYNGLKFHRVINDFMIQGGDPDGNGSGGPGFSFKDEFVDDLKFEKGGVLAMANSGPATNGSQFFITHKDTPWLNGKHTIFGHVVSGMDNVNKIVQDDVMTKIVITRKGAAAKKFDALKVLSDDVKKEAAKKEEAKKVVAAKAAYFAATKAKATTTPSNLKYVITKKGTGVKGAEGSTIYFHYAGYFEDGTLFDSSMAEVAKAYGKYDPNRDAQGGYKAFPFTVGKKDGMIPGFIEALDMMTDGEKAIFFLPSNLAYGEKGAGGVIPPNATLIFEIETYQNQPVK

>Flavobacterium-joh-3

MENGIYAKFNTSKGAILVKLTHDLTPGTVGNFVALAEGNMENKVKPQGQKFYDGLNFHRVIADFMIQGGCPKGTGTGDPGYKFDDEFVPSLKHDRPGVLSMANSGPGTNGSQFFITHVPTPWLDGKHTVFGHVVEGQDIVDAVAQGDALESVEIIRVGEEAQKWNAIESFISLKGARMKREAALKAESEAKMEQLAAGFDKTESGLRYKMIQKGEGKKAEAGKTVSVHYEGSLENGKVFDSSYPRKKPIEFKLGIGQVIEGWDEGIALLQVGDKARFVIPSDLAYGPSGAGGVIPPHATLIFDVELMDVK

>Flavobacterium-lim

MQDGIYAKFNTSKGVILVKLTHDKTPGTVGNFVGLAEGNLENDVKPQGKPYYDGLKFHRVIPDFMIQGGCPLGTGTGDAGYKFDDEFHPELKHDRPGVLSMANAGPGTNGSQFFITHIPTEWLDNKHTVFGHVVEGQDIVDAIVQGDIIESLEIVRVGADAEKWNAIEAFRTFEGSREKRIAEQKRMAEEALEKLAAGFQKTESGLRYQIIQKGNGKQAEKGKTVSVHYQGALDNGMVFDSSYKRKQPIEFALGRGQVIEGWDEGIALLQVGDKARFVIPSYLAYGSRGAGGVIPADATLVFDVELMDVK

>Flavobacterium-omn

MENGIYAKFNTTKGAILVKLAHDLTPGTVGNFVGLAEGNLENKVKPQGVKFYDGLKFHRVIPNFMIQGGCPQGTGTGDPGYKFDDEFHPSLKHNRPGVLAMANSGPGTNGSQFYITHIPTDWLDNKHTVFGHVVEGQDVVDAVEQGDVLESLEIIREGEEAKNWNAIEAFITFKGSRNKRDAALKADVEAAMEKLAAGFEKTESGLRYQFIQRGEGKKAESGKTVAVHYEGSLENGKVFDSSYPRKKPIEFKLGQGQVIEGWDEGIALLRVGDKARFVIPSDLGYGSRGAGGAIPPNATLIFDVELMEVK

>Flavobacterium-phr

MENGIYAKFNTSKGSILVKLTHDLTPGTVGNFVALAEGNMENKVKPQGQKFYDGLNFHRVIADFMIQGGCPKGTGTGDPGYKFDDEFVPSLKHDRPGVLSMANSGPGSNGSQFFITHVPTPWLDGKHTVFGHVIEGQEVVDAVAQGDALETLEIIRVGEEAQKWNAIEAFIGLKGARLKREAALKAESEAKMEQLAAGFDKTESGLRYKMIQKGDGKKAEAGKTVSVHYEGSLENGKVFDSSYPRKKPIEFKLGIGQVIEGWDEGIALLQVGDKARFVIPSDLAYGPSGAGGVIPPNATLIFDVELMDVK

>Flavobacterium-psy-1

MKNKLNLLLVFLFVIANGYAQKGAKKVVKSKTITTQTTTPKYTEGIFAEFDTSKGKILVQLEYAKTPITVANFVSLAEGTNTTVKNDLKGKPFYNGLKFHRVIADFMIQGGDPLGNGSGDPGYKFKDEFTDAKFEKAGILAMANSGPATNGSQFFITHKDTPWLNGKHTIFGYVVSGQDVVNTIAQDDVINKVVIIRKGALAKKFNASSIFTDYYAAKDINAKIEAEKAEKERAIANEVALKEFATGQTTTSGLKYIVLKEGSGLIPTAASNVKVHYTGSFTSGKVFDSSVQRGQPIDFNLNQVIKGWTEGLQLMKEGAKYKFFIPYNLAYGEQGYPGAIPAKSDLIFEVELIKINSDTKQ

>Flavobacterium-psy-2

MNKITKLVLSLTTLVLFSCNSKHDKLSDGLYAEMQTSKGKILLQLEFEKTPITVANFVSLAEGTNTFVEEKFIKKPFYDGLKFHRVIADFMIQGGDPDGNGSGGPGYKFNDEFRDDLIMNKAGILAMANGGPGTNGSQFFITHKETNYLNGRHTVFGHVIEGQDVVNKVAQDDEIIKIEIIRKGEAAKKFDAPKVFKDYFDKQLVAQKENDTKYAKVIADKIAYFATVKAKATKTESGLMYAIVSKGTGKKPAAGTTFFFHYAGYFEDGNLFDSSYENVNREFGKFDPSRSEQNGYQPFPFEAGKKEGLIPGFIEGLEKMSFGDKAVVFIPSALGYGPQGMGGVIPPNTNLIFELEMLEKMPQK

>Flavobacterium-psy-3

MENGIYAKFNTAKGSVLVKLTHDLTPGTVGNFVALAEGNLENKVKPQGTPYYDGLTFHRVIPDFMIQGGCPQGTGTGDGGYKFDDEFHPSLKHDRPGILSMANAGPGTNGTQFFITHVPTSWLDGKHTVFGHVIEGQDVVNAVEGNDVLKTLEIVRVGEEAQKWNAVEAFRTFEGSRAKREAAEKAEAEAKMEQLAAGFDKTESGLRYKMIQKGSGKKAENGKTVSVHYEGSLESGKVFDSSYPRKKPIDFKLGQGQVIEGWDEGIALLQVGDKARFVIPSHLAYGSRGAGGAIPPNATLIFDVELMDVK

>Flavobacterium-rei

MKFKFLFLFCLAVVNIQAQSTKKPAAKPKAAVTKTAAKAVTNPTDGIFATISTTKGDIVLSLEYVKAPVTVANFISLAEGKNPNVKVDRLKGKPFYDGLKFHRVINDFMIQGGDPDGNGSGGPGYSFKDEFVDDLKFEKGGVLAMANSGPATNGSQFFITHKDTPWLNGKHTIFGHVVSGMDVVNKIVQDDVMKKITITRKGAAAKKFDAVKVIGDDAKKDEAKKAEGQKVVTAKAAYFAATKAKATATASGLKYVITQKGTGVKSAEGSTIYFHYAGYFEDGNLFDSSIPAVAKAYGKYDANRDAQKGYQAFPFTVGKKDGMIPGFIEALDMMTDGEKAIFFLPSNLAYGEKGAGGVIPPNATLIFEIETYNTQPAK

>Flavobacterium-riv

MENGIYAKFNTNKGAIVVKLTHDLTPGTVGNFVGLAEGNLENDQKPQGKPYYNGLKFHRVIPDFMIQGGCPIGRGTGGPGYQFDDEFHPSLKHDAPGVLSMANSGPGSNGSQFFITHVATPWLDNKHTIFGNVIEGQDVVDAIAQDDVIETLEIIRVGDEAKNWNAIEAFRTFEGSREKRIAEGKKQAEEAMEKLAAGFDKTDSGLRYKIIQKGDGKKAEKGKKVSVHYKGSLESGEVFDSSYTRKQPIDFQLGVGQVISGWDEGIALLQVGDKARFVIPSHLGYGSRGAGGVIPPDATLIFDVELMDVK

>Flavobacterium-sal

MPFRNLKQNKMQDGIYAKFNTSKGVILIKLTHDKTPGTVGNFVGLAEGNLENDIKPQGKPYYDGLKFHRVIPDFMIQGGCPLGTGTGDAGYKFDDEFHPELKHDRPGVLSMANAGPGTNGSQFFITHVPTDWLDNKHTVFGHVVEGQDIVNEIVQGDIIESLEIIRVGAEAEKWNAVEAFRTFEGSREKRIAEQKKMAEAALEKLAAGFDKTESGLRYKMIQKGNGKQAEKGKTVSVHYSGSLENGMVFDSSYKRKQPIEFSLGRGQVIEGWDEGIALLQVGDKARFVIPSYLGYGSRGAGGVIPPDATLIFDVELMDVK

>Flavobacterium-seo-1

MKFRILFLAFFALLTVQAQETSKKTVKKATPKTSIKQDGIFATITTNKGAIVLALDFQKAPVTVANFISLAQGKNEFVTDKKLQKKPFYDGLKFHRVISDFMIQGGDPQGTGAGGPGYSFKDEFIPELKFDKRGILAMANSGPKTNGSQFFITHKETPWLNGKHTIFGHVVEGMDVVNKIAQDDVIKNITITQKGTMAKKFKAEKVFANYYNNKEQEEKKEAEAKAEAERIQKEKTAALVAEKMAYFSNEKATATTTPSGLVYKIVKKGMAVKPAAGTTVYFHYSGFFEDGNLFDSSLTEVAKSYGKYDERRFVQGGYNPFPFEIGKKDGMIPGFIEAINLLSFGDKIIAFIPAKLAYGEAGAGGVIPPNATLIFEIEIFDKQN

>Flavobacterium-seo-2

MENGIYAKFNTSKGSVLVKLTHDLTPGTVGNFVALAEGNLENKVKPQGTKYYDGLTFHRVIPDFMIQGGCPLGSGTGDPGYKFDDEFHPSLKHDKPGVLSMANAGPGTNGSQFFITHVPTSWLDGKHTVFGHVVEGQDVVDAVAQGDALETLEIIRVGEEAEKWNAVEAFRTFEGNRKKRLEAEKNAAEEAMEKLAAGFEKTESGLRYQFIQRGEGKKAENGKTVSVHYTGQLPDGKVFDSSYPRKKPIEFPLGQGNVIEGWDEGIALLQVGDKARFVIPSHLGYGSRGAGGVIPPNATLIFDVELMDVK

>Flavobacterium-sin

MENGIYAKFNTTKGAILVKLTHDLTPGTVGNFVGLAEGNLENKVKLQGVKFYDGLKFHRVIPDFMIQGGCPQGTGTGDPGYKFDDEFHPSLKHNRPGVLAMANSGPGTNGSQFYITHIPTEWLDNKHTVFGHVVEGQDVVDAVEQGDILESLEIIREGEEAKNWNAIEAFITFKGSRNKRDAALKAEAEAEMEKLAAGFEKTESGLRYQFIQRGEGKKAESGKTVAVHYEGSLENGKVFDSSYPRKKPIEFRLGQGQVIEGWDEGIALLRVGDKARFVIPSHLGYGSRGAGGAIPPNATLIFDVELMEVK

>Flavobacterium-sol

MENGIYAKFNTPKGSILVKLTHDLTPGTVGNFVALAEGNLENKAKPQGTPYYNGLNFHRVIPDFMIQGGCPLGTGTGDPGYKFDDEFHPTLKHNRPGVLSMANSGPGTNGSQFFITHIPTDWLDNKHTVFGHVVEGQDVVDSVAQGDALESVEIVRVGAEAEKWNAVEAFRTFEGSRAKREAAEREAAEAKMEELAAGFDKTESGLRYKMIQKGNGKKAENGKTVSVHYSGQLENGKVFDSSYTRKKPIEFPLGRGNVIEGWDEGIALLQVGDKARFVIPSHLGYGSRGAGGVIPPNATLIFDVELMDVK

>Flavobacterium-sp-1

MENGIYAKFNTVKGSVLVKLEHELTPGTVGNFVGLAEGDLENKVKPQGTKFYNGLKFHRVIPDFMVQGGCPQGTGTGDPGYKFDDEFHPSLKHDKPGILSMANSGPGTNGSQFYITHVPTSWLDGKHTVFGHVVEGQDVVDAIAQGDLLESVEIVRVGEEAEKWNAIEAFITFKGARNKRDIALKADAEAAMEKLAAGFEKTESGLRYQFIQRGEGKQAVAGKTVSVHYEGSLESGKVFDSSYPRKKPIEFPLGQGNVIEGWDEGIALLRVGDKARFVIPSNLGYGSRGAGGAIPPNATLIFDVELMDVK

>Flavobacterium-sp-2

MENGIYAKFNTSKGSILVKLTHDLTPGTVGNFVALAEGNMENKVKPQGQKFYDGLTFHRVIPDFMIQGGCPKGTGTGDPGYKFDDEFHPSLKHDRPGVLAMANSGPASNGSQFYITHVPTSWLDGKHTVFGHVIEGQDIVDAVAQGDALENVEIIRVGEEAEKFNAIEAFVGLKGARLKRDAALKAESEAKMEQLAAGFDRTDSGLRYKMINKGDGKKAEAGKTVAVHYEGSLESGKVFDSSYPRKKPIEFRLGQGQVIEGWDEGIALLQVGDKARFVIPSDLAYGAAGAGGVIPPNATLIFDVELMEVK

>Flavobacterium-sub

MENGIYAKFNTSKGTILVELTYDKTPGTVGNFVGLAEGNLENNEKPQGKPYYDGLAFHRVIADFMIQGGCPQGKGTGGPGYQFDDEFHPDLRHDTPGVLSMANAGPGSNGSQFFITHVATPWLDNKHTVFGKVIEGQDVVDAIQQNDKIETLEIIRVGEDAQKWNAIEAFRTFEGSREKRIAEAKKQADEALDKIAAGFQKTESGLRYQIIQKGDGKQAEKGKTVSVHYKGALVNGQEFDSSYKRKQPIEFPLGKGNVIEGWDEGIALLKVGDKARFVIPSHLGYGSRGAGGVIPPDATLIFDVELMDVK

>Flavobacterium-suc-1

MKSKLLFLLFFGIVSINAQVKKVATPAKKPAAKTAVNPTKVAPGEGLFATIETNKGTIVLALEFQKTPVTVANFVSLAEGTNPYVNKEKLKGKPFYDGLKFHRVIKDFMIQGGDPEGNGSGDPGYKFKDEFTDLKHDKGGILSMANSGPATNGSQFFITHKETPWLNGKHTVFGHVTEGMNVVNAIEQNDVITKVTITRKGALAKKFNAAKVFTDYYANKAEDAKKQAAIDAENKAKKAALEAEAKKAYEAKFGPVIKAKADYIASVRASGTKTPSGLIYQIIQKGTDVKPADGTPIYFHYAGYFEDGNLFDSSYQDVNKAYGKFDANRAAQNGYQPFPFEAGKKTGMIPGFIEALGILNLGEKMIAVMPSNLCYGERGAGGVIPPNANLVFELEIMDKKPETK

>Flavobacterium-suc-2

MENGIYAKFNTSKGEILVKLTHDLTPGTVGNFVALAEGNMENKVKPQGVKYYDGLSFHRVIPDFMIQGGCPQGTGTGDPGYKFDDEFHPSLKHDRPGVLSMANAGPGTNGSQFFITHVPTSWLDNKHTVFGHVIEGQSVVDAVAQGDQLETLEIIRVGDEAKNWNAIEAFISLKGTRMQREAALKAEAEAKMEQLAAGFEKTASGLRYQFIQRGSGKKAENGKTVAVHYEGSLENGKVFDSSYPRKKPIEFRLGQGQVIEGWDEGIALLQVGDKARFVIPSDLAYGPSGAGGVIPPHATLIFDVELMDVK

>Flavobacterium-swi

MKTKLNLLLVFLLVIANGYAQKGTKNVEAKTTANGIFAEFDTSKGKIVVQLEYVKTPITVANFISLAEGTNTIVKADLKGKPFYNGLKFHRVIKDFMIQGGDPLGNGSGDPGYKFKDEITDLKHTKGGILSMANSGPATNGSQFFITHKDTPWLDGKHTVFGSVVSGMDIVNKIAQDDVINKITISRSGAEAKKFDAAKVFANYYTTKDAEAKIQAEKEEKERAIANEIALKEFANGQTTASGLKYIVLKEGTGLAPTPESNVKVHYTGSFTNGKVFDSSVQRGEPIDFGLNQVIKGWTEGLQLMKEGAKYKFFIPYTLAYGEQGYPGAIPAKSDLIFEVELIKINVATK

>Flavobacterium-uro

MQDGIYAKFNTAKGSILVKLTHDKTPGTVGNFVGLAEGQLENNAKPMGKPYYDGLKFHRVIPDFMIQGGCPQGIGTGGPGYQFDDEFHPELKHDKPGVLSMANAGPGTNGSQFFITHVPTNWLDGKHTVFGHVVEGQDVVDAVEGNDSLDSIEIIRVGAEAEKWNAIEAFRTFEGSREKRIAEQKRLAEEAMEKLAVGFQKTESGLRYQIIQKGSGKQAEKGKKVSVHYQGALDNGMVFDSSYKRKQPIEFTLGIGQVIEGWDEGIALLQVGDKARFVIPSYLGYGSQGAGGVIPGDATLVFDVELMDVK

>Flavobacterium-xin

MENGIYAKFSTSKGAILVKLAHDLTPGTVGNFVGLAEGNLENKVKPQGTKFYDGLKFHRVIPDFMIQGGCPLGTGTGDPGYKFDDEFHPSLKHDKPGVLAMANSGPGTNGSQFYITHIPTPWLDGKHTVFGQVVEGQDIVDAVAQGDVLESLEIVRVGEEAQKWNAIEAFITFKGARNKRDASLKADAEAEMEKLAAGFEKTESGLRYQFIQRGEGKQAANGKIVSVHYTGQLPDGKVFDSSYPRKKPIEFPLGQGNVIEGWDEGIALLRVGDKARFVVPSHLGYGSRGAGGAIPPNATLIFDVELMDVK

>Fluviicola-taf

MKKGLKYISAFVLLCITSASLFAQTVALPKDSEPGIYAAFVTTKGTIITKLEADKTPMTVANFVGLAEGNFTIFDSIKISKPFYDGLKFHRVIKDFMIQGGDPQGTGMGGPGYKFPDETRADLTHSGPGILSMANSGPATNGSQFFITHKETSWLNGKHTVFGHVVEGQDVVNKIAQDDVMTKVIIIRNGKAYKSWNATQTFKTAYAKVQAEQEIKKAEQAKLDAIEKERISKCAAMSEAEYSVYLLAEIRKKYPTAQQTASGLVYVIQTQGNGEKAKKGDKVSTQYIGTFLNGTKFDSSRDRNQPLDFTHDSGQMIPGYDEAISIMSKGARGVFVIPYFKAYGAAGRPGAIPPYSDLVFDIELLNIMPAAATPNTPIQIEGTQDK

>Formosa-aga

MQDGLYAKFNTTKGAVVVALEFEKTPGTVGNFVALAEGNLENSVKAQGKPYYDGLKFHRVIPDFMVQGGCPLGTGTGDAGYKFDDEFHPELKHDRPGILSMANAGPGTNGSQFFITHVETPWLDNAHTVFGHVVEGQDVVDAIAQGDSIETLEIIRVGAAAESFNAVEAFRTFEGSREKRLAEARAEQDAALDKIAAGFSKTDSGLRYQILQASKSGKKAKSGQTVSVHYKGQLQDGTVFDSSYKRKEPIEFPIGVGQVISGWDEGIQLLEVGDKARLVIPSDLGYGSRGAGGVIPPDATLIFDVELMEVK

>Formosa-hal

MQDGLYAKFKTTKGDILVALEFEKTPGTVGNFVALAEGNLENSAKPQGKPYYDGLKFHRVIPDFMIQGGCPQGTGTGNPGYKFADEFHPDLKHDRPGILSMANAGPGTNGSQFFITHVETPWLDNAHTVFGHVVEGQDIVDAIAQGDAIENLEIIRVGKAAEDFNAVEAFRTFEGAREKLLAEAKAKQEAELDKISAGFNKTDSGLRYQVLQASKSGKKAKTGQTVSVHYKGQLQDGTVFDSSYKRKEPIEFPIGVGQVIPGWDEGIQLLEVGDKARLVIPSDLGYGAQGAGGVIPPHATLIFDVELMDVK

>Formosa-sp

MNDGLYAKFNTSKGDILVNLEFQKTPGTVGNFVALAQGNLENSVKNQGDPYYNGLKFHRVIPDFMIQGGCPQGTGTGNPGYKFDDEFHPDLKHDKPGILSMANAGPGTNGSQFFITHIPTDWLDGKHTVFGNVIEGQDKVDTIAQGDVLETLEIIAVGADAEAFQAVEAFRTFEGSREKNLEKAKAAVEAELDKLAAGFEKTESGLRYQILQKGSGSKAAKGATVSVHYKGQLTDGTVFDSSYKRKEPIEFALGTGQVIAGWDEGVALLNVGDKARFVIPSHLGYGAQGAGGVIPPNANLIFDVELVEVK

>Gaetbulibacter-sae

MQDGLYAKFNTSKGEILVALEYKKTPGTVGNFVALAEGNLENKVKPQGTPYYNGLKFHRVIPDFMIQGGCPQGTGSGNPGYQFDDEFHPDLKHDGPGVLSMANAGPGTNGSQFFITHVETPWLDNNHTVFGKVVEGQNVVDAIAQGDTIDTLEIVRVGAEAEGFNAVEAFRTFEGSREKRLAEEQAAAEAELDKLAAGFNKTESGLRYQIIQEGNGAKAQKGQNVSVHYKGQLTDGTVFDSSYKRNQPIDFPVGVGQVISGWDEGIQLLKVGDKARFVIPSHLGYGSRGAGGVIPPNATLIFDVELMNVK

>Galbibacter-mar

MDNGIYAKFGTTKGEILVKLTYDKTPGTVGNFVALAEGNMENSVKSQGQPYYNGLNFHRVINDFMIQGGCPLGTGTGDAGYKFDDEFHPELRHDKPGVLSMANAGPGTNGSQFFITHVATPWLDDKHSVFGFVESGQDVVDAIEQGDKLETLTIVRVGEDAQKWNAIEAFRTFEGERERRIAEAKRQAEEALDKLAAGFESTDSGLRYMIIQKGDGPKAEKGQMVSVHYKGQLQDGQVFDSSYARKQPIDFTLGIGQVISGWDEGVGLLQVGDKARFVIPPHLGYGSRGAGGVIPPDAILIFDVELMKVK

>Gelidibacter-mes

MQDGLYAKFITNKGDILVNLEYKRTPGTVGNFVALAEGNLENSVKPQGTPYYDGLKFHRVIPDFMIQGGCPKGTGTGDPGYKFEDEFHQDLRHDAPGVLSMANSGPGSNGSQFFITHIATPWLDDKHTVFGNVIEGQDVVDAIVQGDQIEKLEIVRVGEEAEDFNGVEAFRTFEGSREERIAQERKANEAELDKLATGFDKTKSGLRYKIINKGTGKAAEKGKTVSVHYKGQLADGTVFDSSYKRKQPLEFQVGVGQVISGWDEGICLLNVGDKARLVIPSDLGYGAQGAGGVIPPNAILVFDVELMDVK

>Gillisia-lim

MQDGLYAKFHTSKGEILVELEYKKTPGTVGNFIGLAEGNIENKAIPQGKPYYDGLKFHRVIPDFMIQGGDPKGNGTGGPGYNFEDEIYPELTHDKPGILSMANAGPGTNGSQFFITHIATPWLDGKHTVFGKVIEGQDAVNKIAQGDKLEKLEIIREGEDAKKFNAVEAFRSFSGAKAEREAAAKKQQEEMVGEISQGFEKTSSGLRYKIEEKGTGAKAEKGKTVSVHYKGMLPDGTVFDSSYKRNQPIDFPLGEGHVISGWDEGIQLLNEGGQARFVIPSHLAYGERGAGGVIPPNATLIFDVELVKVK

>Gillisia-mar

MQDGLYAKFHTSKGEILVKLENEKTPGTVGNFVGLAEGNLENKAIPQGKPYYDGLKFHRVIPDFMIQGGDPKGNGTGGPGYNFEDEIHPELTHDAPGKLSMANAGPGTNGSQFFITHIATPWLDGKHTVFGSVVEGQDVVDAIAQGDKLDKVEIIRQGAEAEKFNGVEEFRSFNGAKAEREAAAKKKQEELMGEMSQGFEKTASGLRYKIEQKGDGVQAQKGKTVSVHYKGMLPDGSVFDSSYTRNQPIDFKLGKGQVIQGWDEGIQLLSVGDQARFVIPSHLAYGERGAGGTIPPNATLIFDVELVAVK

>Gramella-ech

MNDGLYAKFHTSKGEILVALEYEKTPGTVGNFVGLAEGKIENEPKAAGEPYYDGLKFHRVIPDFMVQGGDPQGTGAGGPGYQFEDEIHPELKHDAPGKLSMANAGPGTNGSQFFITHVETPWLDGKHTVFGNVVEGQEVVDKIQQGDKLEKVEIVREGSDAENFDAAKAFQDFNSEKEKREEEAKKKAEAELDKLATGFEKTESGLRYQIINKGDGVQAEKGKNVSVHYKGQLADGTVFDSSYKRNKPLEFPVGVGHVIPGWDEGIQLLQVGDKARMVIPSHLGYGERGAGGVIPPNAVLIFDVELMDVK

>Gramella-for-1

MNEGLYAKFHTSKGEILVELEYEKTPGTVGNFVGLAEGNIENEPKAKGEAYYDGLKFHRVIPDFMVQGGDPQGTGAGGPGYQFDDEIHPELKHDAPGKLSMANAGPGTNGSQFFITHVETPWLDGKHTVFGNVVEGQDIVDKIQQGDKLEKVEIIREGSAAENFKATEAFQNFTAEKAGREEEEKKNAEAEVDKLATGFQKTDSGLRYQIIQKGDGAKAEKGKTVSVHYKGQLADGTVFDSSYKRNKPLEFPIGVGHVIPGWDEGIQLLQVGDKARMVIPSHIAYGERGAGGVIPPNAVLIFDVELMEVK

>Gramella-for-2

MKTKSIFLLLAVAVSLFSCNDEYPELEDGMYAEFNTSMGPVIAELYFEETPMTIASFVSLTEGTSKMADSTYKDKKYYDGLIFHRIIDGFVIQGGDPTGTGSGGPGYKYPDEFVDSLSHDSKGILSMANAGPGTNGSQFFITLGPVAQLDGKHTVFGKVVKGQDVVDSIGKVETGPRDRPTKDIVMNEVNIIRKGSAAKNFDAPKVFENQLAEIDAEKEAEAKKKQEMAAKKNEEFKSLEEKADSLDSGLKIYFENKGEGEKPKNGQKVKVSYEGYFADGTIFDTSKKELAQEMGIYDHRRDTSGQYGPMTTVYGPDAPMIPGFKDALQEMKVGDKAVVFIPSAMGYGERGAGGVIPPNTDLIFKIEMVEIVDSSK

>Gramella-sp

MNDGLYAKFHTSKGEILAELEYQKTPGTVGNFVGLAEGKIENKAKSQGEPYYDGIKFHRVIPDFMVQGGDPQGTGVGGPGYKFDDEIHPDLKHDAPGKLSMANAGPGTNGSQFFITHVETPWLDGKHTVFGSVVEGQEIVDKIEQGDKIEKLEIIRKGEAAENFDAAGAFKDFNAEKAQREAEEKKKAEAELDKIATGFERTESGLRYKIIQKGDGKKAEKGNSVSVHYKGQLADGTVFDSSYKRNKPLEFPIGVGHVIPGWDEGIQLLQVGDKARMVIPSHLAYGERGAGGVIPPNAVLVFDVELMDVK

>Hyalella-azt

...MIQGGDPKGAGYGGPGYSFPDEFAGNTKKHDTKGILSMANSGPNTNGSQFFITTVPTPHLDGRHTVFGRVIEGLDVLEAIENVPTGANDKPKDDVKIISIEIIRAGKYKNYDASKTFKEELANLESKKKALLAKQEEETKKALGSITNGMKTTASGLMYKFTSENGGAKPGKGNLVKVHYTGKFVNGQVFDSSVSRGEPIEFPLGNGMVIPGWEEGIGLLGKGDKAVLVIPPSLAYGEQGAGGGIIPPNATLIFEVELVDFK

>Hyunsoonleella-jej

MQDGLYAKFNTSKGEILVALEYKKTPGTVGNFVALAEGNLENSVKPQGTPYYDGLKFHRVIPDFMIQGGCPNGTGAGNPGYQFDDEFHPDLKHDGPGVLSMANAGPGTNGSQFFITHTETAWLDGKHTVFGKVTEGQDVVDAVAQGDTIEHLEIVRVGAEAEAFNAIEAFRTFEGSREKRLAEERAKQEAELEKVAAGFEKTDSGLRYQIIQKGDGVKAEKGKTVSVHYKGQLMDGTVFDSSYKRNQPIDFPLGVGQVISGWDEGISLLNVGDKARLVIPSHLAYGSRGAGGVIPPDANLIFDVELMNVK

>Imtechella-hal

MQNGIYAKFYTSKGDILVKLTHDKTPGTVGNFVGLAEGNLENTAKPQGKPYYDGLTFHRVIADFMIQGGCPEGTGVGGPGYQFDDEFHPELKHNGPGVLSMANAGPGTNGSQFFITHVDTAWLDNKHTVFGFVESGQDVVDTIAQGDVIEKLEIIRVGEEAQKWNAIEAFRTFEGSREQRLAEEKRREEEAIDKVAAGFDKTPSGLRYKIIQKGNGTQAEKGKTVSVHYKGMLVDGTVFDSSYKRNQPIDFALGVGQVIQGWDEGISLLQVGDKARLVIPPQLGYGSRGAGGVIPPNATLVFDVELMKVK

>Jejuia-pal

MQDGLYAKFNTSKGEILVALEYKKTPGTVGNFVALAEGNLENSAKPQGTPYYDGLKFHRVIPDFMIQGGCPQGSGAGNPGYQFDDEFHPELKHDGPGVLSMANAGPGTNGSQFFITHTETAWLDGKHTVFGKVAEGQDVVDAVAQGDVIESVEIIRVGEDAEGFNAVEAFRTFEGSREKRLAEERAKQEAELKKVAAGFEKTDSGLRYQIIQKGNGVKAEKGKTVSVHYKGQLIDGTVFDSSYKRNQPIDFPLGVGQVIPGWDEGIGLLNVGDKARLVIPSNLAYGSRGAGGVIPPDANLIFDVELMDVK

>Kordia-alg

MEDGLYAKFHTSKGEILVSLTYKKTPGTVGNFVALSEGQLENKAKPQGTPYYDGLKFHRVIPDFMIQGGCPLGTGTGSPGYNFDDEIHPELTHSEPGVLSMANAGPGTNGSQFFITHVPTPWLDGKHTVFGHVASGQEVVDTIAQGDTIEKVEIIRQGADAEAFNAVEAFRLFNGAKAEREKAAKEKAEKELDEIATGFDKTESGLRYKIIQEGNGTKAEAGKTVFVHYKGMFPDGGVFDSSYRTNTPIDFPLGEGRVIPGWDEGIALLKVGDKARFVVPPHLAYGARGAGGVIPPNATLMFDVELMDVK

>Kriegella-aqu

MQDGIYAKFNTSKGEILVKLTHDKTPGTVGNFVALAEGNQENTAKSKGEPYYDGLKFHRVIPDFMVQGGCPQGTGTGDAGYKFDDEFHPDLKHDVPGVLSMANAGPGTNGSQFFITHVPTPWLDNKHSVFGRVENGQEIVDAIAQGDAIESLEIIRVGEEAENWDAKKAFETFKNSGKERLAEEKAKQAAELDKVAAGFDETESGLRYKIIQKGAGAKAEKGQKVSVHYEGSLLNGQVFDSSYKRNQPIDFQLGVGQVIRGWDEGISLLEVGDKARFVIPSDLAYGSAGAGGVIPPDATLIFDVELMNVG

>Lacinutrix-jan

MQDGLYAKFNTNKGTILVNLEFKKAPGTVGNFVALAEGNMENSAKPQGTPYYDGLKFHRVIADFMVQGGCPQGAGTGNPGYKFDDEFHPDLKHSGPGILSMANSGPGTNGSQFFITHIATDWLDGNHTVFGNVIEGQDIVNAIAQDDKIETLEIVRVGGSAEKYNAIEAFRTFEGSREKRIAAERAAKAAELDKYSSGFEQTESGLRYQILQKGTGTQAEKGKTVSVHYKGTLTDGTVFDSSYKRNEPIDFALGMGQVIAGWDEGVSLLKVGDKARFVIPSDLGYGSRGAGGAIPPDATLIFDVELMNVK

>Lacinutrix-sp

MNNYKQMIKIFIVALVVGITSASCQDNYKDLEDGLYAEFQTTKGTMVAKLYFEKAPVTVANFVGLAEGTHPSLADSLKGKPFYDGITFHRVMDKFMIQGGDPTASGMGSAGYKFHSEFDQELSHDKAGILSMANSGGLATNGSQFFITEVPLKRLDAFLADGTLKNCNAPRTSCHPVFGELVKGLEVQDSISNVAVSKERSSANKPLEDVVINKLTIIRKGSAAKAFDAPAVFTEQEPLLPQRIEEIKKKQEEIAKEKAKIAADSFKKANADLKGEVYESPTGMVMITTKEGNGVKPKPSDNVYINCAGYFEDGTLFYTTWKDVAKANGTYDEKADENGFYKAFDRKYNTSAGLIPGFREAFLRMKIGDKAKVFIPSFLGYGAAANGPIPANSNLIFDIELMSIK

>Leeuwenhoekiella-bla

MQDGIYAKFHTPKGEILVKLEHEKTPGTVGNFVALAEGNLENSAKKQGNPYYDGLKFHRVIPDFMIQGGCPQGTGTGNPGYQFDDEFDSSLKHDAPGKLSMANAGPGTNGSQFFITHTATPWLDGKHTVFGSVVEGQDVVDAIAQDDTMDKVEIIRQGADAENFNAVEAFRTFEGAREKRIAEERAKKAEALNKLSEGFKETESGLRYQIIQKGDGKKAEKGKMVSVHYKGQLADGTVFDSSYKRNQPLDFQVGVGQVISGWDEGIGLLQVGDKARFVIPSDLGYGSRGAGGVIPPDAILVFDVELMDVK

>Leeuwenhoekiella-mar

MQDGIYAKFHTSKGEILVKLEHEKTPGTVGNFVALAEGNLENSAKKQGTPYYDGLKFHRVIPDFMIQGGCPQGTGTGNPGYQFDDEFDASLKHDAPGKLSMANAGPGTNGSQFFITHTATPWLDGKHTVFGNVIEGQDIVDAIAQGDQIETLEIIREGEEAQDFNAVEAFRTFEGAREKRIAEERAKKAEALNKLSEGFKETKSGLRYQIIQEGNGKKAETGKTVSVHYKGQLADGTVFDSSYKRNQPLDFQVGVGQVIAGWDEGIGLLKVGDKARFVIPSDLGYGSRGAGGVIPPDAILVFDVELVDVK

>Leeuwenhoekiella-pal

MQDGIYAKFHTSKGEILVKLEHEKTPGTVGNFVALAEGNLENSAKKQGTPYYDGLKFHRVIPDFMIQGGCPQGTGTGNPGYQFDDEFDASLKHDAPGKLSMANAGPGTNGSQFFITHTATPWLDGKHTVFGNVIEGQDIVDAIAQGDQIETLEIIREGEEAQDFNAVEAFRTFEGAREKRIAEERAKKAEALNKLSEGFKETKSGLRYQIIQEGNGKKAETGKTVSVHYKGQLADGTVFDSSYKRNQPLDFQVGVGQVIAGWDEGIGLLKVGDKARFVIPSDLGYGSRGAGGVIPPDAILVFDVELVDVK

>Lishizhenia-tia

MTFRIIAVALISLLITSCVSTKIPNFKLEEGIYAEIETNRGVMLLNLEYKKAPLTVANFVGLAEGNLTVFDTVTFDQPYYDGLIFHRVIQNFMIQTGDPKANGTGGPGYRFFDETDNGLTHHDAGVLSMANAGPNTNGSQFFITHRSTAHLNGIHTVFGHIVVGQDIVDQTKQGDTIKELKIIRVGKEARKFDATKTFKIEYEKRKYLFEEEQKRKAELQKQNMARMQKCKTMNISEYKEYFKSIVLEKDSTAVQTASGLMYAVLEEGEGKNPERGDKLQVHYIGTHFYGDKFDSSYDRNQPLSINYQIQGMIPGFDEALALSKKGSKIVAYIPYYLAYGAQGRPGIGPYADLIFSVSLVDIQ

>Lutibacter-mar

MNNGLYAKFNTSKGVILVNLEFEKTPGTVGNFVALAEGNLENNAKPQGKPYYDGLKFHRVIADFMIQGGCPLGTGTGSPGYSFDDEFHPTLKHNAPGILSMANSGPASNGSQFFITHVATPWLDNKHTVFGNVVEGQDVVDAIAQNDIIESIEIIKVGEAAEKFNAVEAFRTFEGSRAKREAEAKAAMKAQMDKIAAGYDETPSGLRYKILQEGTGKKATKGSMVSVHYKGQLLDGQVFDSSYQRKEPIEFQVGVGQVIAGWDEGILLLNVGDKARFVIPSNLAYGSRGAGGVIPPDATLIFDVELMNVL

>Lutibacter-pro

MDNGLYAKFNTSKGVIIVNLEFKKTPGTVGNFVALAEGNLENNAKPQGTPYYDGLKFHRVIADFMVQGGCPLGTGTGSPGYSFDDEFHPDLKHDKPGILSMANSGPASNGSQFFITHVATPWLDQKHTVFGNVIEGQAIVDSIAQDDILESVEIIRVGEQAEKFNAVEAFRIFEGSRAKREAEAKKEAKEAMDKIAAGYTETTSGLRYKILQEGNGKKAEKGKTVSVHYKGQLADGQVFDSSYSRKQPIDFTLGVGQVIAGWDEGIQLLKVGDKARFVIPSNLAYGSQGAGGVIPPDATLIFDVELIDVK

>Mangrovimonas-yun

MQDGLYAKFNTSKGDILVALEFEKVPGTVGNFVALAEGNLENSAKPQGTPYYNGLKFHRVIPDFMIQGGCPQGTGTGSPGYKFDDEFHPDLKHDGPGVLSMANAGPGTNGSQFFITHVETPWLDNMHTVFGKVVEGQDVVDAISQGDVMDAVEIIRIGEAAENFNAIEAFRTFEGSREKRIAAEREAKRAELDKLAAGFEETASGLRYQIIQKGAGKKAEKGKTVAVHYKGQLADGTVFDSSYKRNQPLEFTVGVGQVIAGWDEGIGLLQVGDKARFVIPSDLGYGSRGAGGVIPGDATLVFDVELMDVK

>Maribacter-aqu

MEDGIYAKFNTNKGEILVKLTHDKTPGTVGNFVALAEGDKENSAKGKGEPYYNGLKFHRVIPDFMIQGGCPQGRGTGDAGYKFDDEFHPELKHDAPGVLSMANSGPGTNGSQFFITHVPTPWLDNKHTIFGKVESGQDVVDAIAQDDVIESLEIVRVGDEAKKWDALKAYEDFNASGEARLADEKAKQDAELDKIAAGFDATPSGLRYKMIQKGDGAKAVKGEKVSVHYEGSLLDGQVFDSSYKRNSPIDFQLGIGQVIPGWDEGIALLQVGDKARFVIPSDLAYGSAGAGGVIPPNATLIFDVELMKVG

>Maribacter-ori

MEEGIYAKFNTNKGEILVKLTHEKTPGTVGNFVALAEGNKENSAKSKGEPFYNGLKFHRVIPDFMVQGGCPQGRGTGDAGYKFDDEFHPDLKHDAPGVLSMANSGPGTNGSQFFITHVPTPWLDNKHTVFGKVEAGQDVIDAIEQDDIIESLEIVRVGEEAKNWDAVKAFENFNSSGEARLSAEKAKQDAELDKVAAGFESTASGLRYKMIQKGNGAKAEKGKQVSVHYEGSLLSGDVFDSSYKRNSPIDFQLGIGQVIPGWDEGISLLKVGDKARFVIPSDLAYGSAGAGGVIPPNATLIFDVELMKVG

>Maribacter-sp

MKKAYLFVLTIALALTSCKSSKHADLGDGIFANIQTTKGDMMVRLEHDKTPVTVASFISLAEGNSPFVSENFKDKKYFDGVIFHRVMKDFMIQGGDPTGTGTTGPGYKFKDEFVDSLKHDRAGLLSMANPGPPNTNGSQFFITHKATPWLDGRHTIFGELITGMDVLDSIANVATSQAPQKDKPVVDVVMNTVEIIRNGKEAKKFDAVQIMTDYFAEEEERVAKLKKEGEELKKVYDKMIPQFISELAESKKKAKTFPSGLQILVLVDGKGEKPIIGKQVLVDYAGFLENGKLFDTSKSEVAKKHLKYEDLNRMKANSGGFSPAPMPYSPDAPLFPGFKEALLTMKVGDKIRAFMPPHLGLGEQGGGPIPPNSNLIFDMEITGIVE

>Maribacter-sta

MEEGIYAKFNTNKGEILVKLTHDKTPGTVGNFVALAEGNKENSAKGKGEPYYNGLKFHRVIPDFMIQGGCPQGRGTGDAGYKFDDEFHPELKHDAPGVLSMANSGPGTNGSQFFITHVPTPWLDNKHTIFGHVAAGQEVVDDIAQDDVIESLEIVRVGDTAEKWNALKAFEDFNASGEARLAAEKAKKEAELDKVAAGFESTASGLRYKMIQKGDGDKAEKGQKVSVHYEGSLLDGQVFDSSYKRNSPIDFQLGIGQVIPGWDEGISLLQVGDKARFVIPSDLAYGSAGAGGVIPPDATLIFDVELMKVG

>Mesoflavibacter-zea

MQDGIYAKFNTSKGEILVKLTHDKTPGTVGNFVALAEGNMENNAKPQGTPYYDGLKFHRVIPDFMIQGGCPQGTGTGSPGYQFDDEFHDDLKHGAPGVLSMANAGPGTNGSQFFITHVPTPWLDGKHTVFGHVESGQEVVDAIAQGDIIDSLEIVRVGDEAEQWNAIEAFRTFEGAREKRIAEQKAKAEAQMEKLAAGFDKTDSGLRYKIIQKGNGAQAEKGKTVSVHYEGSLDNGQVFDSSYKRNQPIDFQLGVGQVISGWDEGISLLKVGDKARFVIPSNLAYGSRGAGGVIPPDATLIFDVELMNVK

>Muricauda-ant

MQDGIYAKFNTPKGEILVKLTHDKTPGTVGNFVALAEGDMENAARPQGKPYYDGLKFHRVIADFMIQGGCPLGTGTGDPGYKFDDEFHPELTHNGPGVLSMANAGPGTNGSQFFITHGPTPWLDNKHTVFGFVEHGQEVVDAIAQGDEIESLEIVRVGDEAQNWNAIEAFRVFEGAREKRIAEAKARAEAEMEKLAAGFEKTPSGLRYKIVQKGNGPKAENGKIVSVHYEGSLSNGQVFDSSYKRKQPIDFTLGIGQVIAGWEEGIALLSVGDKGRFVIPSHLAYGSAGAGGVIPPDATLIFDVELMAVK

>Muricauda-lut

MQNGIYAKFNTSKGEILVKLTHDKTPGTVGNFVALAEGNMKNSAKPEGQPYYDGLKFHRVINDFMIQGGCPQGTGTGSPGYQFDDEFHHDLKHDAPGVLSMANAGPGTNGSQFFITHVATPWLDNKHTVFGHVEHGQEVVDAIEQGDTINNLEIVRVGEEAQNWDAVEAFKNFEALKQKRLVAERAKAEAELEKLAAGFEKTESGLRYKIMQKGSGANAEKGKKVSVHYEGMLTDGRVFDSSYQRKQPIDFQLGIGQVMPGWDEGIALLKVGDKARFVIPPHLAYGSRGAGGVIPPNATLLFDVELVKVG

>Muricauda-rue

MKKLFYIIPVFLLVIIGCKSSKYADLGDGIFADIQTTKGDIIIQLEYKKTPVTVANFVSLAEGKNPFVNDEYKDKKFYDGIVFHRIIKDFMIQGGDPTGTGSGNIGYTFKDEFDDSLRHSKKGILSMANRGPKTNSSQFFITHKATPWLDDKHTVFGEVVAGMDVVDTIANVETGAGDKPTTDIVMDHVEIVRNGKEARQFDAVQIMSDYFEEAKAAEEAFKKMKEDLAAQFTEQINEAEETSSGLRILTLEEGEGEQPKVGQKVLVNYAGWLFNGDLFDSNIQEIAEQFNQLNPGRRDQGGYTPFPMDYSPDAQLAAGFREGLLTMKVGDKVRLFIPPHLGYGDNDYGPIPGGSTLVFDIEIVGIQ

>Muricauda-zha

MQDGIYAKFNTSKGEILVKLTHDKTPGTVGNFVALAEGNMENSAKPQGTPYYDGLKFHRVIADFMIQGGCPSGTGTGDPGYKFDDEFHPELVHDKPGVLSMANAGPGTNGSQFFITHVATPWLDNKHTVFGHVVEGQDIVDAIAQGDQIESLEIVRIGDEAENWNAIEAFRVFEGARERRIAEAKAQAEAEMEKLAAGFDKTDSGLRYKIIQKGSGAKAEKGNTVSVHYEGALTNGQIFDSSYKRKQPIDFTLGVGQVIPGWDEGIGLLKVGDKARFVIPSHLAYGSAGAGGVIPPNATLIFDVELMGVK

>Myroides-gua

MENGIYAKFNTTKGVVIAKLTHDKTPGTVGNFVALAEGNLENDFRQQGKPYYDGLKFHRVIPDFMVQAGCPQGTGTGGPGYHFDDEIDSTLTHSKPGVLSMANAGPGTNGSQFFITHVATPWLDGKHTVFGHVVEGQDIVDAIAQGDLIDSIEIIREGEEAKNWNAVEAFRTFEGSRAARIAEERAAAAEALEKVAAGFDKTESGLRYQMIVKGDGKKAEKGKIVAVHYKGSLENGQEFDNSYKRKKPIEFPLGQGHVIEGWDEGIALLQVGDKARFVIPSYLGYGERGAGGAIPPNATLIFDVELMDVK

>Myroides-inj

MENGIYAKFNTSKGEILVKLTEDKTPGTVGNFVALAEGTLENTARPQGQPYYDGLKFHRVIPDFMIQGGCPQGTGTGGPGYTFDDEFDPSLKHDKPGVLSMANAGPGTNGSQFFITHVPTPWLDGKHTVFGHVVKGQDIVDAITQDDKIESIEIIREGDEAKKWNAVEAFRVFEGARERRSVEQRAKADADADKIAEGFEKTPSGLRYQMIVEGTGKKAEKGKTVSVHYKGTLADGKEFDNSYKRKKPIDFPLGQGYVIEGWDEGIALLNVGGKARFVIPSYLGYGENGAGGVIPPNATLVFDVELMDVK

>Myroides-mar

MENGIYAKFNTSKGAILVKLTEELTPGTVGNFVALAEGNLENTARPQGKPYYDGLKFHRVIPDFMIQGGCPLGQGSGGPGYKFDDEFHPALRHDGPGVLSMANAGPGTNGSQFFITHVATPWLDGKHTVFGKVIEGQDIVDAVAQGDILESVEIVRVGEAAEKWNAIEAFRTFEGAREKREAEAKKAALVEVEKLAAGFEETPSGLRYQMIVNGTGKQAEKGKTVSVHYKGSLANGQEFDSSYKRKKPIEFPLGQGYVIEGWDEGIALLKVGDKARFVIPSYLGYGEAGAGGVIPPNAVLVFDVELMDVK

>Myroides-odorati

MENGIYAKFNTSKGAILVKLTEELTPGTVGNFVALAEGNLENSARPQGKPYYDGLKFHRVIPDFMIQGGCPLGQGSGGPGYKFDDEFHPALRHDGPGVLSMANAGPGTNGSQFFITHIATPWLDGKHTVFGKVVEGQDIVDAVAQGDILESVEIVRVGETAEKWNAIEAFRTFEGAREKREAEAKKAALVEVEKLSAGFEETPSGLRYQMIVKGTGKKAEKGKTVSVHYKGALANGQEFDSSYKRKKPIEFPLGQGYVIEGWDEGIALLNVGDKARFVIPSYLGYGEAGAGGVIPPNATLVFDVELMDVK

>Myroides-odoratu

MENGIYAKFNTSKGEILVQLTEDKTPGTVGNFVALAEGKLENSARPQGKPYYDGLKFHRVIPDFMIQGGCPQGIGSGGPGYTFDDEFDATLKHDGPGVLSMANAGPGTNGSQFFITHVATPWLDGKHTVFGRVISGQDIVDAITQDDVIESIEIVRVGEEAQKWNAVEAFRTFEGAREKRIAEQKKAGEEALEKVAAGFERTESGLRYQMIVNGSGKQAEKGKTVAVHYKGALDNGMEFDNSYKRKKPIEFPLGMGHVIEGWDEGISLLRVGDKARFVIPPYLGYGERGAGGVIPPNAILVFDVELMDVK

>Myroides-pha

MENGIYAKFNTSKGAILVKLTEDKTPGTVGNFVALAEGNLENNARPQGKPYYDGLKFHRVIPDFMIQGGCPQGTGSGGPGYQFDDEFHPSLRHDKPGVLSMANAGPGTNGSQFFITHVATPWLDGKHTVFGHVVEGQDVVDAVAQGDLIESIEIVRVGAEAEKFNAVEAFRVFEGSRARREQEAKIAADKAVEELAAGFETTESGLRYQMIIKGTGKKAEKGKIVSVHYKGTLADGKEFDNSFKRKKPIEFPLGQGYVIEGWDEGIALLQVGDKARFIIPSYLGYGENGAGGVIPPNATLVFDVELMDVK

>Nitrospira-bac

MAAETKLDDGLYAKIITPKGDITLKLEFEKTPLTVTNFVGLAEGTKTFKDSKGRTSGRYYDGLKFHRVIPNFMIQGGCPLGSGTGGPGYNFPDEFDPSLKHDSPGILSMANAGPGTNGSQFFITHVPTPWLDNKHSVFGHVVSGQDVVNAIKQGDSITRIEIIRVGDKAEAFKADQQSFDNLLGELDKNKAAREKERLEKDTALIKTKWPNLQTTDSGLMYEVVAKGSGTNKPRPGDTVSAHYTGMFLDGRKFDSSVDRGEPISFPVGAGRVIKGWDEALLDMTKGEKRILVIPPQLAYGSQGRGPIPPNSTLVFEVELLDF

>Nonlabens-dok

MQEGIYAKFDTSKGEILVKLHHDKTPGTVGNFVALAEGNLENTAKDQGTPYYDGLKFHRVIPDFMIQGGDPQGTGSGGPGYKFDDEFHPELRHDGPGVLSMANAGPGTNGSQFFITHIETAWLDDKHTVFGKVIEGQDVVDAIAQGDEIKTVTIVREGDAAQSFNAVEEFRQFEGAAKAREEQARKQADQEIDEIAMGFDKTESGLRYKIINKGDGAKAEKGKTVSVHYKGMLPNGKVFDSSYERKQPIDFALGMRQVIAGWDEGIQLLQVGDKARLVIPSHIAYGSAGAGGVIPPNATLVFDVELVAVK

>Nonlabens-mar

MQEGIYAKFHTSKGEILVQLHYKRTPGTVGNFVALAEGNLENDAKPQGTPYYDGLKFHRVIPDFMVQGGDPQGTGSGGPGYQFDDEFHPELRHDGPGVLSMANAGPGTNGSQFFITHIETAWLDDKHTVFGKVVEGQDVVDAIAQGDSIEKLEIVREGEDAKKFNAVESFRQFEGAGKAREEQARKQAENELDEIAAGFDKTESGLRYKIINKGSGAKAEKGKMVSVHYKGMLPNGKVFDSSFERKKPIDFTLGVGQVIAGWDEGIQLLQVGDKARFVIPSHIAYGSAGAGGVIPPNATLVFDVELVAVK

>Nonlabens-sed

MQEGLYAKFDTTKGEILVQLHYDKTPGTVGNFVALAEGNLENQAKPQGTPYYDGLKFHRVIPDFMIQGGDPQGTGAGGPGYKFDDEFHPELRHDGPGVLSMANAGPGTNGSQFFITHVETAWLDDKHTVFGKVMEGQDVVDAIEQGDEIKKVSILRQGENAEAFNAVESFRQFEGAARAREEQAKKQAEQELEEIAAGFNKTESGLRYQIINHGEGKQAEKGKTVSVHYKGMLANGKVFDSSYSRNQPIDFPLGMGQVIPGWDEGISLLKVGDKARLVIPSHLGYGASGAGGVIPPNAVLVFDVELMDVK

>Nonlabens-ulv

MRLVYDIRFRESVIINNKTSFKLESKITTQLLKIRIMKKVHGLLLLLAVILTAASCEDKYADAPDGIYAEIVTDKGTMLAELYYEAAPLTVANYVALAEGNHPQLGVDSLKGKPYYDGLLFHRVMKDFMIQGGDYTGTGSGNVGYKFDQEIVDTLNHNAKGILSMANAGPNTNGTQFFIMHKETPFLNGKYNVFGKVVEGLAVIDSIAAVPVNAQANNRPIDDVKMQTIRIIRKGKAAKKWDAVEVFKTVQEDKAMAAQEAAELLQKRMAQAPEMQAKKAAQLKEWKAKATKLKDSDVLVYKISEGSGVQPAMGSEVFIDYSGFFMDGKLFDTSDVEIAQTYGNYNERKDQAGAYQEIPVKYDPATPMGAVGFKYAYLTMNYGDKIVAFVPSDLGYGERGAGNVIPPNTELIFEMEILPQD

>Ochrovirga-pac

MNNGLYAKFNTSKGEILVNLEFEKTPGTVGNFVALAEGNMENSAKPQGTPYYDGLKFHRVIPDFMIQGGCPQGTGTGNPGYSFDDEIVAELKHDAPGKLSMANAGPGTNGSQFFITHVATPWLDGKHTVFGSVVKGQDIVDAVAQDDIIESLEIIRVGEAAEKFNAIEAFRTFEGAREARIKAEIEAREQELEKLAAGYEKTASGLRYKIVQKSDSGQKANKGQMVSVHYKGQLSDGTVFDSSYKRKEPIEFALGVGQVIPGWDEGIQLLEVGDKARLVIPSDLAYGARGAGGVIPPNATLIFDVELVKVK

>Olleya-mar

MQDGLYAKFNTNKGEILVALEFEKTPGTVGNFVALAEGNMENEVKPQGTPYYDGLKFHRVIDNFMIQGGCPQGSGTGHPGYKFDDEFHPDLKHSGPGILSMANSGPGTNGSQFFITHVATDWLDGNHTVFGNVVKGQDIVDAIAQGDHIETLEIVRVGEAAENFNAIEAFRTFEGAREKRIAAEREAKRAELDKLAAGFEETKSGLRYQIIQKGNGKKAEKGNQVSVHYKGQLADGTVFDSSYKRNQPLDFQVGVGQVISGWDEGIQLLQVGDKARFVIPSDLGYGSRGAGGVIPPDAVLVFDVELVDVK

>Owenweeksia-hon

MKDGMYARFETSKGLIRVELHFDKTPMTVANFVGLAEGDIENDARDKGQPYYDGLKFHRVIDNFMIQGGDPQGTGAGGPGYNFPDEFDNSLTHDGPGVLSMANAGPGTNGSQFFITHVETAWLDNKHTVFGKVIEGQGVVDAIKQGDLIESLTIDRIGETAQAFDAVATFNEYINKQDEMKQKAKDAAMAKIDALSEGFDKTKSGLRYKITTKGEGKKPVKGKNVSVHYKGMLENGDVFDDSAMRGQPITFPVGVGQVINGWDEGIMLLNEGDEARLVIPPALGYGARGAGGVIPPNAWLIFDVKLVKAG

>Polaribacter-atr

MNNGIYAKFTTPKGEILVQLEHEKAPGTVGNFVALTEGNLENAVKEQGTPYYDGLKFHRVIPEFMVQGGCPLGTGTGNPGYKFDDEFHPDLKHDAPGKLAMANSGPATNGSQFYITHVPTPWLDGKHTVFGSVIEGQDVVDAIAQGDEITTVEIIKVGAEAEAFNAVEAFRTFEGSREKREAEEKAKQKALLDTVAAGYDETASGLRYQILQKGTGKKATKGAGVSVHYKGQLLDGTVFDSSYKRKEPIDFNVGVGQVISGWDEGIQLLQVGDKARFVIPSNLAYGSAGAGGVIPPDATLIFDVELMDVK

>Polaribacter-dok

MNNGIYAKFKTPKGEILVQLEHEKAPGTVGNFVALTEGNMDNAVKPQGTPYYDGLKFHRVIPDFMIQGGCPQGTGTGNPGYKFDDEFHPDLKHDAPGKLAMANSGPATNGSQFYITHVPTPWLDGKHTVFGTVVEGQDVVDAVAQGDEMTVEIIKVGDEAENFNAIEAFRTFEGAREKREAAEKAKQKELLDSVAAGYDETESGLRYKILQNGEGKQATKGAGVSVHYKGQLLDGTVFDSSYKRKQPIDFNVGVGQVISGWDEGIQLLKVGDKARFVIPSNLAYGAQGAGGVIPPNATLIFDVELMDVK

>Polaribacter-irg

MKDGIYAKFTTPKGEILVQLEHEKTPGTVGNFVALSEGNLENSVKGQGTPYYDGLQFHRVIPDFMVQGGCPQGSGTGNPGYKFDDEFHPDLKHDAPGKLAMANSGPATNGSQFYITHVPTPWLDGKHTVFGAVIEGQDVVDAIAQGDALSAVEILRVGAVAEAFNAVEAFRTFEGSREKREVEEQAKQKELLDSVAIGYDETASGLRYQILQEGTGKQATKGAGVSVHYKGQLLDGTVFDSSYKRKEPIDFNLGVGQVIAGWDEGIQLLKVGDKARFVIPSNLAYGSAGAGGVIPPDATLIFDVELMGVK

>Polaribacter-MED

MNNGIYAKFKTPKGEILVQLEHEKAPGTVGNFVALTEGNMENAAKPQGTPYYDGLKFHRVISDFMIQGGCPQGTGTGNPGYKFDDEFHPDLKHDAPGKLAMANSGPATNGSQFYITHVPTPWLDGKHTVFGSVVEGQDIVDAVAQGDEMTVEIVRVGEEATNFNAIEAFRTFEGAREKREAEEKAKQKELLDSVAAGYDETASGLRYKILQNGNGKQATKGAGVSVHYKGQLLDGTVFDSSYKRKQPIDFNVGVGQVISGWDEGIQLLKVGDKARFVIPSNLAYGAQGAGGVIPPNATLIFDVELMDVK

>Polaribacter-rei

MNNGIYAKFKTPKGDILVQLEHEKTPGTVGNFVALAEGNLENSSKEQGTPYYDGLKFHRVIPDFMIQGGCPQGTGTGNPGYKFDDEFHPDLKHDAPGKLAMANSGPATNGSQFYITHVPTPWLDGKHTVFGSVIEGQDVVDAVAQGDEMSVEIIKVGEEAENFNAIEAFRTFEGAREKREAEAKAAQKEMLDKVAAGYDETASGLRYKVLQKGDGKQATKGAKVSVHYKGQLLDGTVFDSSYKRKQPIDFNVGVGQVISGWDEGIQLLKVGDKARMVIPSNLAYGSAGAGGVIPPNATLIFDVELMDVK

>Polaribacter-sp

MNNGIYAKFTTSKGDILVNLEFEKTPGTVGNFVALAEGNLENSVKDQGTPYYDGLKFHRVIPDFMIQGGCPQGTGTGNPGYKFDDEFHPDLKHNRPGVLAMANSGPATNGSQFYITHVPTPWLDNKHTVFGAVVEGQDIVDAVAQGDELTSIEIIRVGTEAEAFNAVEAFRTFEGSREKREAEAKAAQKEMLDKVAAGYDETASGLRYQILQKGSGKKATKGAGVSVHYKGQLLDGTVFDSSYKRKQPIDFNVGVGQVISGWDEGIQLLQVGDKARFVIPSNLAYGSAGAGGVIPPDATLIFDVELMDVK

>Polaribacter-vad

MNNGIYAKFTTTKGDILVQLEHEKTPGTVGNFVALAEGNLENAVKEQGTPYYNGLKFHRVIPDFMIQGGCPQGTGTGNPGYKFDDEFHPDLKHDAPGKLAMANSGPATNGSQFYITHVPTPWLDGKHTVFGSVIEGQDIVDAVAQGDELKSIEIIRVGDAAEKFNAVEAFRTFEGSREKREAEEKAKQKELLDSVAAGYDETASGLRYKVLQKGDGKKATKGAGVSVHYKGQLLDGTVFDSSYKRKQPIDFNVGVGQVISGWDEGIQLLQVGDKARFVIPSDLAYGSAGAGGVIPPDATLIFDVELMDVK

>Pricia-ant

MQDGIYAKFNTSKGEILVKLTHEKTPGTVGNFVALAEGKQENKVKPKGEPYYDGLKFHRVIPDFMIQGGCPQGIGTGDAGYKFDDEFHPDLNHSKPGVLSMANSGPGTNGSQFFITHVPTPWLDNKHTVFGHVESGQDIVDDIAQGDHIESLEILRVGDKAKDWDAVAAFETFKSSKEKRIAEQKKNQEAELDKISAGFESTESGLRYKMIQEGNGAKAQKGQTVSVHYEGSLINGQVFDSSYKRKQPIDFQLGAGQVISGWDEGISLLKIGDKARFVIPSELGYGSAGAGGVIPPNATLLFDVELMDVK

>Psychroflexus-gon

MVNGIYAHFNTSKGKIIVQLTFDKTPGTVGNFIGLAEGKIKNTAKDLGTPYYNDLKFHRVIDNFMVQGGDPMGNGTGGPGYQFDDEIHPELKHDRAGVLSMANAGPGTNGSQFFITHGPTGWLDGKHTVFGYVLEGQDIVDSIEMKDALNSVEIERVGDEAKAFDAVESFENFKASKDEREARQNAEHEEQLEAISKGFQKTKSGLRYQIINEGSGPQPKKGQNISVHYKGSLVNGNVFDSSYKRKEPIEFPVGAGHVIEGWDEGLLLLKEGTKAQFVIPPDLAYGDQEVGGVIPANSILIFDLELMKVKS

>Psychroflexus-sed

MENGIYAHFNTEKGKITVQLTYDKTPGTVGNFIGLAEGKIENSAKDLGTPYYDGTKFHRVIENFMIQGGDPKGNGTGGPGYQFDDEIHPDLKHNRPGVLSMANAGPGTNGSQFFITHEPTPWLDGKHSVFGYVVEGQDVVDSIEVNDKLNSLEIERVGEEAEAFDAVERFEKFKSSKAEREAELKAEASKKLDEISKGYKETESGLRYQIINEGSGPKPEKGQNVSVHYKGSLVDGTVFDSSYKRKQPIEFPVGAGHVIEGWDEGLLLLNKGTKAQFVIPPHLAYGDREVGGVIPANSILVFDLELVDIK

>Psychroflexus-tor

MRTLRLSFAALVGILLFSCSSQYPDLEDGLYAEFQTSMGDFVTELHYDKVPMTVGNFVALAEGEHPLVDEEYQDQKFYDSIIFHRVIDKFMIQGGDPLGTGQGGPEYEFADEIDSVLTHKKGVLSMANAGADTNGSQFFITLVPTPHLDGKHSVFGELVVGMEVVDSIGKVETKKPGDKPVEDIVIETVNIIRKGSDAKNFDAVDAFTTGAEKAKVAKVEEEVARKAVLEEASEGFKVTDSGLRYLITEKNPNGTSPKAKDMVSVHYTGYLLDGTKFDSSLDRNQPIEFPVGTGRVIRGWDEGIMLLKTGEKAELVIPSELAYGPRQTGPIPPNSILKFEVELIDIVNK

>Psychroflexus-tro

MENGLYAHFNTAKGKITVKLTHDKTPGTVGNFVGLAEGKIQNSAKDLGTPYYDGLVFHRVIDNFMIQGGDPQGNGTGGPGYQFDDEIHPELKHNQSGVLSMANAGPGTNGSQFFITHEPTPWLDGKHTVFGYVVQGQDVVDSIKMKDDLNSVEIERVGEEAKAFDAVKSFEKFNASKAEREAELKAEAARKLDEISKSYKETESGLRYQIINKGSGAKPKKGQTVSVHYKGSLEDGTVFDSSYKRKQPIEFPVGAGHVIEGWDEGLLLLNQGTKAQFVIPPHLAYGDREVGGVIPANSILIFDLELVDIK

>Psychroserpens-bur

MQDGLYAKFNTTKGEILVALEFQKTPGTVGNFVALAEGNLENEVKPQGTPYYDGLKFHRVIPDFMVQGGCPQSTGTGNPGYKFDDEFHPDLKHDAPGILSMANAGPGTNGSQFFITHIETPWLDNNHTVFGKVITGQDVVDAIAQGDAIDSLEIIRVGSNAENFNAVEAFRTFEGSREKRVVAERDAASAELDKLSAGFEETKSGLRYQIIQKGTGKAAEKGKTVSVHYKGQLADGTVFDSSYKRNAPLDFQVGVGQVIAGWDEGICLLNVGDKARLVIPSDLGYGSAGAGGVIPPDATLVFDVELMDVK

>Psychroserpens-dam

MQDGLYAKFNTTKGEILIALEFQKTPGTVGNFVALAEGNLENEVKPQGTPYYDGLKFHRVIPDFMIQGGCPQGTGTGNPGYKFDDEFHPDLKHDKPGILSMANAGPGTNGSQFFITHIATDWLDNKHTVFGNVIEGQDVVDAIAQDDAINTIEIIRVGTEAENFNAVEAFRTFEGSREQRIAAEREAAKAELDKLAAGFDETESGLRYQIIQKGTGKSAEKGNMVSVHYKGQLADGTVFDSSYKRNAPLDFQVGVGQVIAGWDEGICLLNVGDKARLVIPSDLGYGSAGAGGVIPPDATLVFDVELMDVK

>Riemerella-ana-1

MKKVLSLCVALLAILSLTNCNPIYKKMNIEKEFYNALPEGVYAKMETSKGSMIIQFNDKESPVTVANFVGLAQGTIENKAKKKGEPYYDGIIFHRVIKDFMIQGGDPTGTGMGDPGYKFDDEKNDLKHEGKGYLSMANSGPNTNGSQFFITEVATPWLDGRHTVFGKVIQGLEVIDAIANVEKGAQDKPKQDVVIQKVEVFTKGDSYKNYDAAKIFNEGKSKIQERNKAYIAKKEAEAAKKLEELKAGMTATASGLLYKITKSTEGKAPKAGDMVAVHYAGRLTNGQEFDNSFKRGEPIEFPVGTGRVIKGWDEGILLKEGEQATLLIPSNLAYGERGAGGVIPPNAWLLFDVELVKVK

>Riemerella-ana-2

MNIEKEFYNALPEGVYAKMETSKGSMIIQFNDKESPVTVANFVGLAQGTIENKAKKKGEPYYDGIIFHRVIKDFMIQGGDPTGTGMGDPGYKFDDEKNDLKHEGKGYLSMANSGPNTNGSQFFITEVATPWLDGRHTVFGKVIQGLEVIDAIANVEKGAQDKPKQDVVIQKVEVFTKGDSYKNYDAAKIFNEGKSKIQERNKAYIAKKEAEAAKKLEELKAGMTATASGLLYKITKSTEGKAPKAGDMVAVHYAGRLTNGQEFDNSFKRGEPIEFPVGTGRVIKGWDEGIMLLKEGEQATLLIPSNLAYGERGAGGVIPPNAWLLFDVELVKVK

>Riemerella-columbin

MKKILILCFAFVALISLTNCNPIYKKMNLDKEFYNALPDGVYAKMETSKGDMIIQFFDKEAPVTVANFVGLAEGKIENKAKKKGEPYYDGIIFHRVIKDFMIQGGDPTGTGMGDPGYKFDDERNDLKHEAKGYLSMANSGPNTNGSQFFITEVATPWLDGRHTVFGKVIKGLEVIDTIADVEKGAQDKPKEDVVIKKVEVFTKGDAYKSYDAAKIFNEGKGKIQEQNKAYDAKKEAEAAKKLEELKQGMTTTASGLMYKKTHETDGKAPKAGDSVAVHYAGRLVNGQEFDNSFKRGEPIEFPVGMGRVIKGWDEGIMLLKEGEKATLLIPSDLAYGARGAGGVIPPNATLIFDVELVKVK

>Riemerella-columbip

MKKIVSLCFATIAILTLTNCNPIYKKMNIDKSFYEALPDGVYAKMETSKGDMIIKFFDKESPVTVANFVGLAQGKIENKAKTKGVPFYDGLIFHRVIKNFMIQGGDPQGTGMGSPGYKFDDEKNDLKHDAEGYLSMANSGPNTNGSQFFITHVPTPWLDGKHTIFGKVVKGIEVIDAIANVKTAAQDRPEQDVKIEKVEIFTKGEAYKHYDAAKTFNEGKAKIQENNQAYFEKKAAEEAKKLERMKSEMTQTPSGLFYKITQTTEGKAPKAGDVVAVHYSGKLLNGMEFDNSYKRHEPIDFPVGTGRVIKGWDEGIMLLKEGEKATLLIPPALGYGARGAGGVIPPNAWLLFDVELVKVK

>Robiginitalea-bif-1

MQEGIYAKFHTTRGEILVKLTHEKTPGTVGNFVALAEGDQPNSARDAGKPYYDGLKFHRVIPDFMIQGGCPSGTGTGDPGYKFDDEIHPDLKHNQPGVLSMANAGPGTNGSQFFITHVPTPWLDGKHTVFGHVASGQEVVDSIAQGDRIETLEIVRVGDAAESWDALAAFDEFRDAVRKKEEAAKAEMEQQLESHAEGFETTPSGLRYKMLETGDGEKPSRGDRVAVHYEGSLLNGTVFDSSVRRGDPIEFLLGEGQVIPGWDEGIQLLRVGDKARLLIPAELAYGSRGAGGVIPPNAPLLFDVELVAIR

>Robiginitalea-bif-2

MKQTFSLLLLIGLLATGCKSNQYADLGDGLYAEMQTNQGEIILRLEYEQTPVTVANFVTLAEGTSPFVSEEFKGKPYYDGVTFHRVMKDFMIQGGDPTGTGRGTPGYRFSNEIVDSLVHDRKGILSMANSGGTKTNGSQFFITHAPTPWLDGIHTVFGEVVQGIEVVDSIAAVPVDPASNKPLDSVVMETVEIVRKGRDAKDFDAVEVMRNYFAQEEAAEAARRQRLQDYVAEFADQKARAESLPSGLQYLVLNEGEGPKPSIGQRVLVNYSGWLEDGTLIDSSDESVAREFGELDRLMQMHRGSLTPYPMPYSPDTQLIAGFKEALLLMEVGDKWRVFIPSHLAYGDQGNGPVPPGADMIFDLEIIEIEGSE

>Runella-lim

MTEKEGIYAIFDTAKGEITVELLYEKCPMTVANFVGLAEGTMENSAKPLGQPFYDGLKFHRVISKINGDSSDFMVQGGDPQGTGSGGPGYRFPDEIDATLRHDRPGVLSMANAGPGTNGSQFFITHVPTPWLDGKHTVFGFVLDGQEVVNQMKTNDAINAVKIVRQGEKAQAFKADKAALDAHIGQLAEKQKEAEKAEVETFKQWALANYPTAQFTASGLGYVVNNEGTGKEAVAGKNVSVHYTGSFQNGQVFDSSHSRRQPIDFRLGEGRVIKGWDEGIALMKEGAKYTLLIPYQLGYGKNGYGPIPAKATLIFETELVKVG

>Salegentibacter-mis

MNEGLYAKFYTTKGQILVELEYEKAPGTVGNFVGLAEGEIENEAFPQGKPYYDGLKFHRVIPNFMVQGGDPQGTGVGGPGYKFEDEIHPELKHDAPGKLSMANAGPGTNGSQFFITHVETPWLDGKHTVFGSVIQGQDVVDSIKQGDEIEKLEIIRSGEKAENFNAVESFRQFDGAKAKREEAAKKREEELLGKLSQGFKTTESGLRYKIEKKGEGAKPEKGQTVSVHYKGMLTDGSVFDSSYKRNQPLEFPVGVGHVISGWDEGILLLNVGDQARFVIPSHLAYGERGAGAVIPPNAALVFDVELVAVK

>Salegentibacter-sale

MNDGLYAKFYTTKGQILVELEYEKVPGTVGNFVGLAEGKLENDAFPQGKPYYNDLKFHRVIPNFMVQGGDPQGTGVGGPGYKFEDEIHPDLKHDAPGKLSMANAGPGTNGSQFFITHTETPWLDGKHTVFGSVIEGQDVVDSIKQGDKIEKLEIIRSGENAQNFNAVESFRQFDGAKAKREDAAKKREDELLGKLSEGFKTTESGLRYKIEKKGEGAKPEKGQTVSVHYKGMLTDGSVFDSSYKRNQPLEFPVGVGHVISGWDEGILLLNVGDQARFVIPSHLAYGEQGAGGVIPPNAALVFDVELVAVK

>Salegentibacter-sali

MNDGLYAKFYTTKGQILVQLEFEKAPGTVGNFVGLAEGKLENEAFPQGKPYYNDLKFHRVIPNFMVQGGDPQGTGVGGPGYKFEDEIHPDLKHDAPGKLSMANAGPGTNGSQFFITHTETPWLDGKHTVFGSVIEGQDVVDSIKQGDKIEKLEIIRSGESAQNFNAVEAFRQFDGAKAERETAAKKREEELLGELSQGFKTTESGLRYKIEKKGEGAKPEKGQTVSVHYKGMLTDGSVFDSSYKRNQPLEFPVGVGHVISGWDEGILLLNVGDQARFVIPSHLAYGEQGAGGVIPPNAALVFDVELVAVK

>Salinimicrobium-cat

MNDGLYAKFHTSKGEILVELEYDKTPGTVGNFVGLAEGNLENEAKPQGNPYYDGLIFHRVIPDFMIQGGDPKGTGVGGPGYQFEDEIHPDLKHDRPGILSMANAGPGTNGSQFFITHVATPWLDGKHTVFGHVIEGQDVVDAIKQGDKIEKLEIIREGEAAKNFNAVETFRQFNGAKAEREAAAKRQQEELMGELAQGFEKTSSGLRYKMVSEGNGKKAEKGKTVSVHYKGMLADGTVFDSSYKRKQPIEFPLGKGHVIEGWDEGIQLLKEGDKARFVIPSHLGYGARGAGGVIPPNATLVFDVELMKVK

>Sediminibacter-sp

MQDGLFANFHTNKGDILVALEFEKAPGTVGNFVALAEGNLENSVKPQGQPFYDGLKFHRVIPDFMIQGGCPQGTGTGNPGYKFDDEFHPDLKHDGPGVLSMANSGPGTNGSQFFITHVETPWLDNAHTVFGKVVEGQDVVDAIVQGDKIETLEIVRKGDAAEKFNAIEAFRVFEGSREKKIQEAREAAKAELEQLGAGFEETKSGLRYKVIQKGDGKPAKKGDMVSVHYKGQLADGTVFDSSYKRNKPLDFQVGVGQVISGWDEGICLLNVGDKARLVIPSDLGYGSAGAGGVIPPNATLVFDVELMAVK

>Sediminicola-sp

MQDGIYAKFNTSKGEILVKLTHDKTPGTVGNFVALAEGNMENSVKPQGTPYYDGISFHRVIPDFMIQGGCPLGTGTGDGGYKFDDEFHKDLKHDAPGVLSMANAGPGTNGTQFFITHIATPWLDNKHTVFGHVAEGQDIVDAIAQGDKIKNLEILRVGEAAEKWNAIEAFRTFEGSREKRLAEEKAKQAEELDKVAAGFDETASGLRYKIIQKGNGAKAEKGQTVSVHYEGALVDGTVFDSSYKRNQPIDFKLGVGQVIPGWDEGISLLKVGDKARFVIPSNLAYGSAGAGGVIPGDATLVFDVELMKVK

>Soonwooa-bua

MKKLLFVPVVLTLLNCKTLEIDKETYNSLSDGLYAKMQTSKGEMLIKLEDEKAPVTVANFVGLAEGKIPNKAKADKVPFYDGTIFHRVIKDFMIQGGDPQGTGMGDPGYKFADEKNDLKHTGKGILSMANSGPNTNGSQFFITEVATPWLDGRHTIFGKVVKGEDVIDTIANVEKGPNDKPKEDIVLQKLTVFSKGDQYKKYDPAKTFEEGKAKIEEKNKAFAAKEEADRLKKLQEFQANQDKLVNELKAGMQSTPSGLFYKITKSNPSGKAPKTGDVVSVHYAGKLVDGEEFDNSFKRGEPIQIPIGVGQVIKGWDEGIQLLKEGEAATLLIPASLGYGERGAGGVIPANAWLIFDVELVKVGG

>Sphaerochaeta-coc

MSNKLADGLYAAIHTTKGDIILSLAYDKVPMTVANFVGLAEGALNLEKKGTPYYDGLVFHRVIPEFMIQGGCPKGTGTGGPGYYFPDEFDDSLLHDGPGTVSMANAGPGTNGSQFFITHVATPRLNGKHSVFGHVVEGQDVVDKIKQGDKINSINILRVGADAENYSVTQEAFSALVEKVARVAEEKKEQERKAVDQELKNRWPDAVKTPSGLRYVIVQEGKGTDSPARGAKVTVHYTGSLLNGKVFDSSTQRGTPAQFKIGEVIEGWNEALLTMHKDEKRTLIIPPELGYGTHGYPGVIPPDSYLVFDVHLISW

>Sphaerochaeta-glo

METKNLTDGLYAVLHTNKGDITLLLEYEKTPMTVANFIGLAEGTLNINGKNKPFYSNIKFHRVIENFMIQSGCPKGNGTGGPGYTFPDEFDDSLKHTGPGVLSMANAGPGTNGSQFFITHVATPWLDGKHSVFGRVVEGLDVVNAIAQGDSIKTVEIVRKGSQAEAFVVSREAFTQYVLAAEEKNHKRESNEKAKLEDELKNRWPDAILTSSGLRYVVKKAGDGKKSPVQGQKVTVHYTGSLLDGRIFDSSVRRGSPAQFAIGEVIEGWNEALMTMSAGEQRTLIIPPDLGYGTMGYPGVIPPNSYLVFDVELIKF

>Sphaerochaeta-ple

MEKKELADGLYAVMHTAKGDILLSLEYKKTPMTVANFIGLTEGTLNINNEGKPFYNGLTFHRVIENFMIQGGCPKGNGTGGPGYTFPDEFDDSLKHVAPGCLSMANAGPGTNGSQFFITHVATPWLDGKHTIFGHVLEGQDIVNAIAQGDKLTSVEILKVGSEAEAFEVSRETFSKHVAAAEEKAKEREKKESSKIENELKNRWPDAIVTESGLRYVVTCKGTGTKNPKRGQTVTVHYTGTLLDGRVFDSSVRRGTPAQFSIGEVIEGWNEALVTMTAGEKRTLIIPPQLGYGTMGYPGVIPPNSYLVFDVELIKF

>Spirochaeta-afr

MRYIRMIITAAVLAAVAGCTSANAIPEGGRMSQGELVKLTAADGSTVDHELENGLYAVMETSKGSILLNLEYQRTPLTVTNFVGLAEGTIDNDRGDGPFYDGLTFHRVIENFMIQGGDPQGTGSGGPGYRFPDEIRSELRHDAPGILSMANAGPGTNGSQFFITHEPTPWLDGRHTVFGAVVAGQDVVNAVAQGDTIEQLRILRVGDEAQQFTADQSDFDSRLEELRREEMQGQEAFMEEQRQDIADRFDDLQDGPDGLQYTITAAGSGEPAREGQTVRINYTGSFVHGQVFDSSEGREPLEFQLGGGQIIPGFDLAVRGMQPGEKRTAVIPPHLAYGEQGAGGVIPPNAYLVFEIELLD

>Spirochaeta-lut

MSQHPDGLYARMDTTKGDILLSLEFEKTPVTVMNFVGLAEGSLDTDAKKGQPFYDGLTFHRVIDDFMIQGGDPKGNGTGGPGYRFPDEFHSQLRHDQPGILSMANSGPNTNGSQFFITHVPTPWLDDKHSVFGRVVEGMDVVNAIEQGDSINTLEIIRKGEAAENFQVTQELFDQELQNAAERQAAYQQEQQEADRKRAEAIIPDAQVDSNGIRYKITQEGSGSSPDPGQTVRVHYTGAFLDGRVFDTSQQRGPAEFPIGVGRLIPGWDIMVPQMKVGEKRIFVLPPEFAYGDQGAGGVIPPGAYLYFEIELLDIVE

>Spirochaetes-bac

MAKDLQDGLYAKITTNRGDIVVSLSYDKTPMTVSNFVGLAEGVLNLKSPGTPYYDGLKFHRVINDFMIQTGCPQGTGTGGPGYTFPDEIVDGLKHTGPGVLSMANAGPGTNGSQFFITHVPTPWLDGKHSVFGQVVEGLDIVNSIAQGDVMKKVEILRVGNDAEAFVVSKESFANEVVKAQDLENSRLSKIQEVLERELQNRWPDAIKTPSGLRYVVTQEGKGTKTPKNGANVTVHYTGTLLDGKMFDSSVRRGEPAQFAVGQVIEGWNEALVTMKKGEKRTLVIPPELGYGQQGYPGVIPPNSYLVFDVELLDF

>Tamlana-nan

MKDGLYAKFNTTKGEILVALEYKKTPGTVGNFVALAEGNLENKVKPQGTPYYNGLKFHRVIPDFMIQGGCPQGTGSGNPGYQFDDEFHPDLKHDGPGVLSMANAGPGTNGSQFFITHVETPWLDNNHTVFGKVVEGQDVVDAIAQGDVIDTLEIVKVGAEAEAFNAVEAFRTFEGAREKRIEAEKKAAEAELDKLATGFSKTESGLRYQIIQEGNGKKAEKGKTVSVHYKGQLADGTVFDSSYKRNAPIDFPLGMGQVISGWDEGIQLLKVGDKARLVIPSHLGYGSRGAGGVIPPNATLVFDVELMDVK

>Tenacibaculum-dic

MNNGIYAKFTTPKGAILVNLEYEKTPGTVGNFVALAEGNLDNTAKPQGTPYYNGLKFHRVISDFMIQGGCPQGTGTGNPGYKFEDEFHPDLKHDAPGKLSMANAGPGTNGSQFFITHTPTPHLDGNHTVFGNVVEGQNVVDAIAQNDTMDIEIIRVGETAEKFNAVEAFRTFEGSREKREAEEKAKQAELLDTVAKGYDETPSGLRYRILQNGDGKQATKGANVSVHYKGQLLDGTVFDSSYKRKQPIDFAVGVGQVIAGWDEGILLLKVGDKARFVIPSNLAYGKAGAGGVIPPNATLIFDVELMAVK

>Tenacibaculum-mar

MNNGIYAKFTTPKGEILVNLEYEKTPGTVGNFVALAEGNLENSAKPQGTPYYNGLKFHRVIPDFMIQGGCPQGTGTGNPGYKFDDEIHPELKHNAPGKLSMANAGPGTNGSQFFITHVPTPWLDGKHTVFGNVIEGQEIVDAIAQGDDMQVEIIRVGEEATSFNAVEAFRSFEGAREKREAAEKAKQKELLDSVAAGYDETPSGLRYKILQNGSGKQATKGANVAVHYKGQLLDGTVFDSSYKRKQPIEFAIGVGQVIAGWDEGIQLLKVGDKARLVIPSHLAYGAAGAGGVIPPNATLIFDVELMDVK

>Tenacibaculum-ovo

MNNGIYAKFTTPKGDILVNLEFEKTPGTVGNFVALAEGNLENEAKPQGTPYYNGLKFHRVIGDFMIQGGCPQGTGTGNPGYKFDDEFHPDLKHDAPGKLSMANAGPGTNGSQFFITHVATPHLDGNHTVFGSVTEGQSVVDAVAQGDSMDVEIIRVGETAEKFNAVEAFRSFEGSREKREVEEKAKQKELLDSVAKGYDETPSGLRYKILQNGDGKQATKGANVSVHYKGQLLDGTVFDSSYKRKQPIDFAIGMGQVIAGWDEGIQLLKVGDKARFVIPSNLAYGSAGAGGVIPPNATLIFDVELMDVK

>Tenacibaculum-sp

MDNGIYAKFTTPKGDILVNLEYEKTPGTVGNFVALAEGNLENSAKPQGTPYYNGLKFHRVIADFMIQGGCPQGTGTGNPGYKFDDEIHPELKHDAPGKLSMANAGPGTNGSQFFITHVATPWLDGKHTVFGSVIEGQDVVDAVEQEDTMDVEIIRVGEVAEKWNAVEAFRTFEGAREKREAEAKAKQKELLDSVAAGYDETPSGLRYKILQNGDGKQATKGAMVSVHYKGQLLDGTVFDSSYKRKQPIDFAIGVGQVISGWDEGIQLLKVGDKARLVIPSDLAYGAQGAGGVIPPNATLIFDVELMNVK

>Treponema-azo

MVLMAESAFAASDDAALGDGLFARITTAKGDIVIRLEYQKVPLTVCNFVALAEGKMTTAGGKRYYDGLTFHRVIADFMIQGGDPVGNGTGGPGYKFPDEFDPSLRHNGPGVLSMANAGPDTNGSQFFITHVATPHLDDHHTVFGRVVQGQQVVNAIRQGDRIERVTIIRNGPQANAFKADQAAFDALLRNASAAKTSKLSSQKSAALAEIEKKYPGAVTTASGLKYIVQKQGSGAKPTAGKTVSAKYKGMFLSGEVFDNSDVHGGATDFQVGVRRIIPGMDEALLDMAPGEKRTVIIPPELAYGERGAGNGAIPPNSFLVFELELVKIKELLSVK

>Treponema-bre

MKKLSAILLCAGYIICAGSCSPMEKSMKAIEGKDGVFAIMQTSRGNIVLELYYKQTPLTVTNFVGLAEGTLNATNGKPFYDGLKFHRVIADFMIQGGDPKGNGTGGPGYRFSDEFVDELKHDVPGTLSMANAGAGTNGSQFFITHVPTPWLDGKHTVFGRVVEGQDVVNKIKQGDLIEKVTIVRQGADAQAFTASQDDFDERSAAAKKAAVEKKEKGRAATIALIEKNFPGARKTADGIYYVVTKEGSGSKVGKGKAVSVHYKGYLLDGSVFDSSEGRGTLDFSTAAGQMIPGFDTMVQDMKTGEKRTIVLPPEQAYGSAGAAGVIPGDAYIAFDVELVRVK

>Treponema-cal

MSKGISTALSAHLNRVLFLLLASTAASLPSDSYLGEGLFARISTDRGDIIVKLEYKKTPLTVCNFTGLAEGKLNATGGKPYYNSLTFHRVISDFMIQGGDPLGNGTGGPGYKFPDEIVDDLKHDSPGVLSMANAGPNTNGSQFFITHKATPWLDGKHTVFGHVIEGQEVVTAIKQGDKIKSITIIRNGAEAKAFKNDQAAFDSLLKNAVAASAERIKAKRQADLASIATKYPGAQTTDNEVRFIIIKEGNGAKPQPGSTVSVNYKGMFLNGEVFDASDFHGGPIQFQVGTGRVIPGWDQMVLDMKKGEKRLIILPPERAYGEQGAGGVIPPNAFLVFEMELVAIK

>Treponema-den

MKKLWIMIIAIAFMILVAGTAAAIIITNSGSEKGDKNMNNLKNIEALKEDGLYAAIDTDKGLIVLKLFYKETPLTVCNFVGLAEGTLDAAKGKPFYDGLTFHRVIADFMIQGGDPTGTGSGGPGYRFPDEIVEDLKHDGPGVLSMANAGPGTNGSQFFITHVETPWLDGKHTIFGRVVEGQNVVDSIQQGNKIKTVKIIRTGNEAKAFKTDQEAFYKYLAETKESEKRRAEAFAKKMEDLIKTKYSPAKLDDDGVYSFVVKQGKGDTPKQGQTLTMKYKGSLLENGKVFDDSDMHKPLEFPVGLGRVIPGFDSQSAKMTLGEKRIIIIPPHLAYGEAGAGGVIPPNAYLVFELELLNIK

>Treponema-mal

MSKATDSIKDKDGVFAVMETTRGDIVLELYYKQTPLTVTNFVGLAEGALDAAKGKPFYDGLKFHRVISKKNGDEQDFMIQGGDPQGTGRGGPGYVFPDEIVSDLRHNKAGILSMANAGAGTNGSQFFITIVPTPWLDGKHTVFGRVVAGQDVVNSMLVNDVIKKVTIIRNGSEAKKFTATQADFNRLKKEAGEKVFKEIEAKFPGAKKDANGIYYLVKKDGSGNKAGKGRQAAVHYTGSLLNGQVFDSSQGRSPLRFTTGAGQMIPGFDVMVQDMKLHEKRTIVLPPNMAYGANGIPGVIPGNAYLVFEVELTELR

>Treponema-pha

MTGIKQFAGFKQQKYKKNTEEKMRTNKLLLIILSIAFILIATATATAIIFTGKTTKEGSIMDETLEATLKDGLYAVMETDKGKIVLQLFYDKTPLTVCNFVGLAEGKLDAAKGKPFYDGLKFHRVIADFMIQGGDPLGNGTGGPGYKFADEFVPSLKHSGPGILSMANSGPNTNGSQFFITHVATPWLDGKHTVFGKVVEGMPVVNKIAQGDKIISIKIFRKGEAAKKFIADQEHFDTYQEAAGKMAEVHKENQKKAMIAKIKEKWPKAQQTGNGIFYLVTKEGSGATAQRGQTLTMKYKGSLLETGKVFDDSDMHEPIQFQAGSGQLIPGFDQQAAEMKKGEKRTIILPPELAYGSRGAGGVIPPDAYLVFELELLAIQ

>Treponema-pri

MRILTKLSIGSVLLALMAGTASCAPGGSSSTSPAAPRNSANAALGDGLFARIHTGRGDIVLRLEYQKTPMTVCNFVALAEGKMNAAGGKPFYDGLTFHRVIADFMIQGGDPVGNGSGGPGYRFPDEFDSSLKHDGPGVLSMANAGPGTNGSQFFITHVATPWLDGKHTIFGRVVQGQDVVNAIKQGDKIESVTIIRNGQAANDFKADQAAFDTLRQNSAAAEAAKARAGRDADIAQIKTRYPDANVTPSGIYYIIQKAGTGAKPEKGKTVSVNYKGMFLNGEVFDNSELRGEPLQFPVGAGRVIQGWDETLLDMKLGEKRLVVIPPELAYGERGAGNGAIPGNSFLVFEMELVQIR

>Treponema-put

MKMKKLWIMIIAIAFMILVAGTAAIIITNSGLEKGDKKMSNLKNIEALKEDGLYAAIDTDKGLIVLKLFYKDTPLTVCNFVGLAEGILDAAKEKPFYDGLTFHRVIADFMIQGGDPTGTGSGGPGYKFPDEIVENLKHDGPGVLSMANAGPGTNGSQFFITHVETPWLDGKHTVFGRVVEGQNVVDAIQQGDKIKTVKIIRTGNEAKAFKTDQEAFYKYLSDSKESEKRRAEAVAKKMEGLIKTKYSPAKLDDDGVYSFVIKQGKGDTPKQGQTLTMKYKGSLLENGKVFDDSDMHKPLDFPVGVGRVIQGFDSQAAKMTLGEKRIIIIPPHLAYGEAGAGGVIPPNAYLVFELELLNIK

>Treponema-sac

MKKIILAGIIGCLLVSFGCGSSNNKKAAAKTASSSTGVKNMDSNVLKGKEGVFAVMETSKGVIYLELFYKKTPLTVTNFVGLAEGTLDAAAGKPFYDGLKFHRVIADFMIQGGDPKGTGCGGPGYCFTDEFRDGLKFDKPGYLAMANSGPETNGSQFFITHVPTDWLNGKHTIFGQVVDSASQDVVNKIAQDDKIVSVKIVRQGADAEKFTATQGDFDRLARVVKKVNEEAFTEGCDKSSNGTFFKILKEGAGSKIGKGKTVFVEYKGYLANGMVFDASSGMMKGAHEGLEFRTNGGQMIPGFDIQVQDMKVGETRKIVIPPELAYGSRGIPHAGIPGGAYIAFDVMVVSAK

>Treponema-soc

MEKSIKSIQGKDGVFAVMETSRGNIVLELYYKDTPLTVVNFVGLAEGTLDAAKGKPFYDGLTFHRVISKGNGDDQDFMIQGGDPRGNGTGGPGYSFPDEIVDKYAFTSGGLLAMANSGANTNGSQFFITIVPTPWLTGKHTIFGKVLEGQDIVNKTKQGDAIKKITIVRNGEEAKKFTASQSDFNKLSADALKAAAARKEAAFADQIKTIEKNFANFEKNADGIYYKIKKAGSGEKTGKGKKVSVDYKGYLIDGRVFDSSEGRAPLSFTTAAGQMIPGFDAMVQDMKKGESRTIVIPPDLAYGERGYPGVIPENAYIAFDITLQ

>Treponema-suc

MKKFIGALLFVCVLIFYGGCKPMKSLEGKEGLFAVINTSKGDIVLELFYKQAPLTVTNFVGLAEGTLDAAKGKPFYDGLKFHRVIKDFMIQGGDPKGNGTGGPGYNFEDEIVDGLEFTGSGVLAMANAGPGTNGSQFFITHVETPWLNGHHTIFGHVVDEASQATVNSIAKDDEIKSIKIIRQGAEAEKFTATQTDFDRFALEAQKKALEAKEKANASKIAEVEKNFPGFEKDKNGIYYKVIREGTGNKCGARKSVATEYKGYLVDGSVFDQSKGRGPLEFQTGAGQMIPGFDIMVQDMKLGEKRTVVIPSDLAYGDRGYPGVIPGGAYIAFDIELVKIK

>Treponema-vin

MRLNKIAVLVLPVTFLIIATAAAAAIILTGDSEKGRKMNAISAELQKDGVYAVIDTNKGDIVLELFYKETPMTVCNFVGLAEGTLDAAKGKPFYDGLTFHRVIANFMIQGGDPDGKGTGGPGYRFPDEFVDNLKHNTAGILSMANAGPGTNGSQFFITHVPTPWLDGKHTVFGRVVKGQDVVNKIVQGDRMNSISIIRKGADAQKFTATQKDFNAYLAGAEERAKQRTAQRREKNEAFIKQKFPNAVRTESGIFYTITKEGKGAQAQIGKTLTMKYKGSLLDGTVFDDSDMHEPLKFVAGAGQLIAGFDQQAAQMAVGEKRTIVIPPELAYGSRGAGGVIPPDSYLVFDLELLSVK

>Ulvibacter-lit

MQDGIYAKITTEKGEILGKLTYKETPGTVGNFVALAEGNLENKAKPQGTPYYDGLKFHRVLQDFMIQGGDPAGTGAGGPGYKFDDEFHPELKHDKPGIFSMANAGPGTNGSQFFITHVPTDWLDNKHTVFGEVVEGQDVVDSVAQGDTMLSVEIIRVGDDAKNWNAVEAFRQFTGSRAEREAAARKKQEEAMADLVQGFDKTDSGLYYKHIQKGDGASPEKGQTVAVHYKGMLADGTEFDSSYKRGNPIEFAVGVGQVIEGWDEGILLLKTGDKARFVIPSNLAYGEAGAGGVIPPNATLIFDVELMNVKG

>Vitellibacter-aqu

MQDGIYAKITTEKGEILIKLTHDKTPGTVGNFVALAEGNLENSAKPQGTPYYDGLKFHRVIPDFMIQGGDPNGTGAGGPGYNFDDEFHQDLRHDTPGVLSMANAGPASNGSQFFITHVATPWLDNKHTVFGNVVEGQDVVDSVAQGDTMQKVEIIRVGEEAKKWNAVEAFRSFTGEREQRIAKMKEAQEAELKKISEGFDRTDSGLLYKIIQKGNGKKAEKGKTVSVHYKGALTDGTEFDSSYKRKQPIDFQLGVGQVIAGWDEGIQLLQVGDKARFVIPSHLAYGERGAGGVIPPSATLIFDVELMDVK

>Wenyingzhuangia-fuc

MNNGLYAKFNTSKGAILVNLEFEKTPGTVGNFVALAEGNMENNARPQGKPYYDGLKFHRVIADFMIQGGCPQGTGSGNPGYKFDDEIVAELKHDTPGVLSMANAGPGTNGSQFFITHTATPWLDGKHTVFGKVVEGQDIVDAVEQGDLIESLEIVRVGEAAEKFNAIEAFRKFEGAREAKLKAEKEAAEMELEKLAAGYDKTNSGLRYKVIQASGSGKKAAKGKMVSVHYKGQLADGTVFDSSYKRKEPIEFAIGVGQVISGWDEGIQLLEVGDKARLVIPSHLGYGPNGAGGVIPPNATLIFDVELVKA

>Winogradskyella-sp

MQDGLYAKFNTSKGEILVNLEFEKTPGTVGNFVALAEGNMENSAKPQGNPYYDGLKFHRVIPDFMIQGGCPQGTGTGNPGYKFDDEIHPDLKHDAPGKLSMANAGPGTNGSQFFITHIATDWLDGKHTVFGNVIEGQDIVDAIAQDDTIDSLEIVKVGEAAENFNAIEAFRTFEGAREKRIAAERDAAKAELDKLAAGFDETESGLRYQIIQKGDGQKAEKGNMVSVHYKGQLADGTVFDSSYKRNSPLDFQVGVGQVIPGWDEGICLLNVGDKARLVIPSDLGYGPAGAGGVIPPNATLVFDVELMGIN

>Weeksella-vir

MKKVILILTLLLITVSCSVKIPSTMSKEEFKNLEDGLYANMVTNKGTMLIKLYEEQAPMTVANFTGLAEGKIKNSAKAEGVPYYDGVIFHRVIKDFMIQGGDPDGRGTGGPGYSFEDEFDASLKHDKKGVLSMANSGPATNGSQFFITEVPTPWLDGRHAIFGQVVDGLDVIDTIANVEKDGQDKPKENVVIEKVEVIRKGEHYKEYDPAESFETARANHAKKLEEQKAKEAAEKAKEVERLKELESKAQATDSGLKYVIEKEGEGAKPVHGDAINVHYTLRLADGEKVDSSYDRQDPLRVTVGVTGLIQGWMEALTMFNRGSKVMLIIPSHLGYGDRGAGGVIPPNATLYFDMEVLED

>Winogradskyella-psy

MQDGIYAKFNTTKGAILVNLEFEKAPGTVGNFVALAEGNLENSVKPQGNPYYDGLKFHRVIPDFMIQGGCPQGTGTGNPGYKFDDEFHPDLRHSGPGVLAMANSGPGTNGSQFYITHIATDWLDNKHTVFGNVVEGQDIVDAIAQGDKIETIEIIRQGDAAEKFNAIEAFRTFEGSREKRIAEEREALRAELDKLAVGFDETKSGLRYQIIQKGDGKKAEKGNTVSVHYKGQLADGTVFDSSYKRNSPLDFQVGVGQVIAGWDEGICLLNVGDKARLVIPSDLGYGAAGAGGVIPPNATLVFDVELMGVK

>Xanthomarina-gel

MQDGLYAKFNTNKGEILVALEYKKTPGTVGNFVALAEGQMENSAKPQGRPYYDGLKFHRVIPDFMIQGGCPQGTGTGNPGYKFDDEFHPELKHSGPGVLSMANAGPGTNGSQFFITHVETPWLDNNHTVFGQVVTGQEVVDAIAQGDTIETLEIIRVGAEAEAFNAIEAFRTFEGAREKRVAAEREAKRAELDKLAQGFEETESGLRYQILQKGTGKQAEKGKTVSVHYKGQLTDGTVFDSSYKRKQPLEFPVGVGQVIPGWDEGIQLLQVGDKARLVIPSDLGYGSRGAGGVIPPDATLIFDVELMGVS

>Zobellia-gal-1

MQDGIYAKFNTDKGEILVKLTHDKTPGTVGNFVALAEGKQENTAKAKGEPYYDGLNFHRVIPDFMIQGGCPQGTGTGDAGYKFDDEFHPDLNHSEPGVLSMANAGPGTNGSQFFITHVPTPWLDNKHTVFGHVQSGQDVVDSIAQGDKINSVEIVRVGDDAEKWDALAAFENFKTSKEQRLAEEKAKQAAELDKVAAGFDETESGLRYKLIQKGDGPQAQKGQTVSVHYEGSLLNGQVFDSSYKRNQPIDFQLGVGQVIPGWDEGIALLKVGDKARLVIPSDLAYGSAGAGGVIPPNATLLFDVELMGVK

>Zobellia-gal-2

MRKFYYLITLAVLLASCKSQYAELGDGLFADIHTTKGDIIVKLEYKKTPVTVANFVSLAEGKNPFVTDSLKGKKFYDSIIFHRVIKDFMIQGGDPTGTGRGNPGYKFKDEFNDSLVHDRKGILSMANSGPKTNGSQFFITHKETPFLNGRHTVFGHVIEGLDVVDSIANVETSQDRMTQDRPLEDVIMTSVEIVRNGKEAKKFDAVKVMTDYFAEEEALIAAFNKMKSEFKAELEKQKAEAEELASGLKIMRTKEGEGDTPRTGQQVLVRYAGFLEDGTLFDSNYEEVATKYNQFDERRKQGGGYEPIPMEYSPDAALIPGFKEGLLNMKIGDKVRIFIPSHLGYGEQGAGPIPPNSNLVFDLEITGIAE

>Zobellia-uli

MQDGIYAKFNTDKGEVLVKLTHDKTPGTVGNFVALAEGKQDNTAKAAGEPYYDGLKFHRVIPDFMVQGGCPQGTGTGDAGYKFDDEFHPDLNHSKPGVLSMANAGPGTNGSQFFITHVPTPWLDNKHTVFGHVESGQDVVDAIAQGDQINSVEIVRVGDEAQNWDALASFEKFKSSKEVRLAEEKAKQAAELDKIAAGFDETESGLRYKMIQKGTGAQAEKGQTVSVHYEGSLLNGQVFDSSYKRNQPIDFQLGVGQVIPGWDEGIQLLKVGDKARLVIPSSLGYGSAGAGGVIPPNATLLFDVELMGVK

>Zhouia-amy

MQDGIYAKFNTSKGEILVKLTHDKTPGTVGNFVALAEGNMENDAKPQGNPYYDGLKFHRVIADFMIQGGCPQGSGFGGPGYQFDDEFHPELTHNRAGVLSMANSGPGSNGSQFFITHVETPWLDGKHTVFGFVESGQDIVDAIAQGDLIETLEIIRVGDEAENWNAIEAFRTFEGEREKRIAAEKAQKEEELEKVSAGFDKTASGLRYKIIEKGNGPKAEKGQTVSVHYEGALTNGQVFDSSFKRKQPIDFTLGVGQVIPGWDEGIGLLNVGDKARFVIPSELAYGSRGAGGVIPPDAVLVFDVKLVDAK

>Zunongwangia-man

MDNGIYAKFHTTKGEILVALEYEKAPGTVGNFVGLAEGNLENEAKPQGTPYYDGLKFHRVIPDFMVQGGDPQGTGVGGPGYKFDDEIHPELKHDAPGKLSMANAGPGTNGSQFFITHVETPWLDGKHTVFGSVVKGQDIVDSIAQGDKIEKLEIIREGEAAKNWNAVETFRQFNGAKAEREAAARKQQEELLGELAQGFEKTESGLRYKIEQEGDGKQAEKGKTVSVHYKGRLADGTVFDSSYKRNQPLEFPVGVGHVIAGWDEGILKLKVGDQARFVIPSHLGYGERGAGGVIPPNATLIFDVELMDVK

>Zunongwangia-pro-1

MDNGIYAKFHTTKGEILVALEYEKAPGTVGNFVGLAEGNLENEAKSQGTPYYDGLKFHRVIPDFMVQGGDPQGTGSGGPGYKFDDEIHPDLKHDAPGKLSMANAGPGTNGSQFFITHVETPWLDGKHTVFGSVVEGQDVVDSIAQGDKIEKLEIIREGEEAKNWNAVETFRQFNGAKAEREAAALKQQEELLGKLAQGFEKTDSGLRYKIEKEGDGKQAEKGKTVSVHYKGRLADGTVFDSSYKRNQPIEFPIGVGHVIAGWDEGILKLKVGDQARFVIPSHLGYGERGAGGVIPPNATLIFDVELMDVK

>Zunongwangia-pro-2

MKKFSLLLLSAIVLAFTACKDDYPDLEDGMYAKFDTSMGPFIAELYYEQTPITVASFVSLAEGNSTMVDSTYKNKNFYDGIIFHRIIDGFVIQGGDPTGTGRGGPGYRFPDETIDSLSHESKGILSMANAGPGTNGSQFFITLAPTTNLDGRHTVFGKVVKGQDVVDAIGKVETDPGDRPVKDVVINSVEIIRKGKSARKFDAPKVFENELQKIKEAEEEEARKLEEAKAENKAMFEKYQDEAKTLDSGLGIYILKEGEGPKPKIGQNVGVDYEGYFTDGGIFDTSKEEVAKKWDIFNEMRSMQGGYAPLNISYGPDAPMIAGFNEGVQQMKVGDQAILYIPSHLAYGERGRGPIEPNTDLVFIVDLVDIK
