# Supplementary material for: Bioinformatic Analysis Reveals Conservation of Intrinsic Disorder in the Linker Sequences of Prokaryotic Dual-family Immunophilin Chaperones
Source: Comput Struct Biotechnol J. 2017 Dec 30;16:6–14. doi: 10.1016/j.csbj.2017.12.002 (PMC5852385; doi:10.1016/j.csbj.2017.12.002)
Supplement: Supplementary Material 2 — List of all CFBP organisms. In the “Others” category, two multicellular organisms are highlighted (nematode, shrimp). The nematode (hookworm in man and other mammals) has a unique C-terminus and short N-terminus. In the arthropod (small shrimp), the CYN part is missing ~60 aa, including the “N-terminal loop” (19–24 in hCyPA). GAL66646.1 was not used because of its short N-terminus. [file mmc2.pdf]

Supplementary Material 2: List of all CFBP organisms

In the "Others" category, two multicellular organisms are highlighted (nematode, shrimp).
The nematode (hookworm in man and other mammals) has a unique C-terminus and short N-terminus.
In the arthropod (small shrimp), the CYN part is missing ~60 aa,
including the "N-terminal loop" (19-24 in hCyPA).
GAL66646.1 was not used because of its short N-terminus.

Species	Domain structure	Accession #	Phylogeny
**Phylum Spirochaetes**			
Borrelia crocidurae	CYN-FKBP	WP_025400716.1	Spirochetes
Borrelia crocidurae str. Achema	CYN-FKBP	AFI31197.1	Spirochetes
Borrelia duttonii	CYN-FKBP	WP_012538168.1	Spirochetes
Borrelia hermsii DAH	CYN-FKBP	WP_012422177.1	Spirochetes
Borrelia hispanica	CYN-FKBP	WP_024655403.1	Spirochetes
Borrelia persica	CYN-FKBP	WP_024653864.1	Spirochetes
Borrelia recurrentis	CYN-FKBP	WP_012538884.1	Spirochetes
Borrelia turicatae	CYN-FKBP	WP_011772360.1	Spirochetes
Sphaerochaeta coccoides	CYN-FKBP	WP_013740452.1	Spirochetes
Sphaerochaeta globosa	CYN-FKBP	WP_013608701.1	Spirochetes
Sphaerochaeta pleomorpha str. Grapes	CYN-FKBP	AEV29721.1	Spirochetes
Spirochaeta lutea	CYN-FKBP	WP_037548329.1	Spirochetes
Spirochaeta Africana	CYN-FKBP	WP_014456714.1	Spirochetes
Spirochaetes bacterium GWC2_52_13	CYN-FKBP	OHD32850.1	Spirochetes
Treponema azotonutricium ZAS-9	CYN-FKBP	WP_015710234.1	Spirochetes
Treponema brennaborense	CYN-FKBP	WP_013757191.1	Spirochetes
Treponema caldaria DSM 12168	CYN-FKBP	WP_013967678.1	Spirochetes
Treponema denticola	CYN-FKBP	WP_002680321.1	Spirochetes
Treponema maltophilum	CYN-FKBP	EPF32053.1	Spirochetes
Treponema phagedenis	CYN-FKBP	EFW38648.1	Spirochetes
Treponema primitia ZAS-2	CYN-FKBP	WP_015709565.1	Spirochetes
Treponema putidum	CYN-FKBP	WP_044979872.1	Spirochetes
Treponema saccharophilum	CYN-FKBP	WP_002706539.1	Spirochetes
Treponema socranskii	CYN-FKBP	ERF61397.1	Spirochetes
Treponema succinifaciens DSM2489	CYN-FKBP	WP_013700291.1	Spirochetes
Treponema vincentii ATCC 35580	CYN-FKBP	WP_006188840.1	Spirochetes
**Phylum Bacteroidetes (mostly Flavobacteria)**			
Apibacter mensalis	CYN-FKBP	WP_055425959.1	Flavobacteria
Aquimarina agarivorans	CYN-FKBP	WP_010523173.1	Flavobacteria
Aquimarina longa	CYN-FKBP	WP_062053470.1	Flavobacteria
Aquimarina macrocephali	CYN-FKBP	WP_024768822.1	Flavobacteria
Aquimarina megaterium	CYN-FKBP	WP_025666008.1	Flavobacteria
Aquimarina pacifica	CYN-FKBP	WP_025743611.1	Flavobacteria
Aquimarina spongiae	CYN-FKBP	WP_073315242.1	Flavobacteria
Aequorivita capsosiphonis	CYN-FKBP	WP_026450730.1	Flavobacteria

Aequorivita sp. D-24	CYN-FKBP	WP_062622682.1	Flavobacteria
Aequorivita sublithincola	CYN-FKBP	WP_014781106.1	Flavobacteria
Aequorivita vladivostokensis	CYN-FKBP	WP_045081509.1	Flavobacteria
Algibacter lectus	CYN-FKBP	GAL78289.1	Flavobacteria
Arenibacter certesii	CYN-FKBP	WP_026812083.1	Flavobacteria
Arenitalea lutea	CYN-FKBP	WP_019387329.1	Flavobacteria
Arenibacter palladensis	CYN-FKBP	SHF91915.1	Flavobacteria
Bacteroidetes bacterium MedPE-SWsnd-G1	CYN-FKBP	OIQ37634.1	Flavobacteria
Bacteroidetes bacterium OLB9	CYN-FKBP	KXK39650.1	Flavobacteria
Bergeyella zoohelcum	CYN-FKBP	WP_002663385.1	Flavobacteria
Bizionia argentinensis	CYN-FKBP	WP_008635427.1	Flavobacteria
Bizionia paragorgiae	CYN-FKBP	SDZ72777.1	Flavobacteria
Capnocytophaga canimorsus Cc5	CYN-FKBP	WP_013997216.1	Flavobacteria
Capnocytophaga canimorsus Cc5	CYN-FKBP	WP_013997324.1	Flavobacteria
Capnocytophaga cynodegmi	CYN-FKBP	CEN32541.1	Flavobacteria
Capnocytophaga gingivalis ATCC 33624	CYN-FKBP	WP_002665439.1	Flavobacteria
Capnocytophaga gingivalis ATCC 33624	CYN-FKBP	WP_002667121.1	Flavobacteria
Capnocytophaga sputigena Capno	CYN-FKBP	WP_002679844.1	Flavobacteria
Capnocytophaga ochracea F0287	CYN-FKBP	WP_015782565.1	Flavobacteria
Capnocytophaga sp. Oral taxon 324	CYN-FKBP	WP_009411812.1	Flavobacteria
Capnocytophaga sp. Oral taxon 324	CYN-FKBP	WP_009411339.1	Flavobacteria
Cellulophaga algicola DSM 14327	CYN-FKBP	WP_013551507.1	Flavobacteria
Cellulophaga algicola DSM 14327	CYN-FKBP	WP_013549201.1	Flavobacteria
Cellulophaga baltica	CYN-FKBP	WP_025614578.1	Flavobacteria
Cellulophaga geojensis KL-A	CYN-FKBP	EWH14126.1	Flavobacteria
Cellulophaga lytica DSM 7489	CYN-FKBP	WP_013619851.1	Flavobacteria
Cellulophaga lytica	CYN-FKBP	APU08853.1	Flavobacteria
Chryseobacterium angstadtii	CYN-FKBP	WP_048507577.1	Flavobacteria
Chryseobacterium antarcticum	CYN-FKBP	WP_051803836.1	Flavobacteria
Chryseobacterium aquaticum	CYN-FKBP	WP_056014146.1	Flavobacteria
Chryseobacterium arachidis	CYN-FKBP	WP_072959387.1	Flavobacteria
Chryseobacterium arthrosphaerae	CYN-FKBP	OCA73542.1	Flavobacteria
Chryseobacterium artocarpi	CYN-FKBP	WP_065394712.1	Flavobacteria
Chryseobacterium balustinum	CYN-FKBP	WP_079466867.1	Flavobacteria
Chryseobacterium bovis	CYN-FKBP	WP_076782045.1	Flavobacteria
Chryseobacterium caeni	CYN-FKBP	WP_034671276.1	Flavobacteria
Chryseobacterium carnipullorum	CYN-FKBP	WP_073335862.1	Flavobacteria
Chryseobacterium chaponense	CYN-FKBP	SIS91935.1	Flavobacteria
Chryseobacterium contaminans	CYN-FKBP	WP_073300115.1	Flavobacteria
Chryseobacterium cucumeris	CYN-FKBP	WP_062670346.1	Flavobacteria
Chryseobacterium culicis	CYN-FKBP	SEH32004.1	Flavobacteria
Chryseobacterium daeguense	CYN-FKBP	WP_034752384.1	Flavobacteria
Chryseobacterium formosense	CYN-FKBP	WP_034676143.1	Flavobacteria
Chryseobacterium gallinarum	CYN-FKBP	WP_053328736.1	Flavobacteria
Chryseobacterium gambrini	CYN-FKBP	WP_076390416.1	Flavobacteria
Chryseobacterium gleum	CYN-FKBP	WP_002980016.1	Flavobacteria
Chryseobacterium greenlandense	CYN-FKBP	WP_059136803.1	Flavobacteria

Chryseobacterium gregarium	CYN-FKBP	WP_034761027.1	Flavobacteria
Chryseobacterium haifense	CYN-FKBP	WP_084186087.1	Flavobacteria
Chryseobacterium hungaricum	CYN-FKBP	SDG10680.1	Flavobacteria
Chryseobacterium indologenes	CYN-FKBP	WP_047098396.1	Flavobacteria
Chryseobacterium indoltheticum	CYN-FKBP	WP_076558645.1	Flavobacteria
Chryseobacterium jejuense	CYN-FKBP	SDI33762.1	Flavobacteria
Chryseobacterium jeonii	CYN-FKBP	KIA90404.1	Flavobacteria
Chryseobacterium joostei	CYN-FKBP	WP_076355767.1	Flavobacteria
Chryseobacterium koreense	CYN-FKBP	WP_083993749.1	Flavobacteria
Chryseobacterium limigenitum	CYN-FKBP	SFZ90634.1	Flavobacteria
Chryseobacterium luteum	CYN-FKBP	WP_034707310.1	Flavobacteria
Chryseobacterium molle	CYN-FKBP	SHK62632.1	Flavobacteria
Chryseobacterium oleae	CYN-FKBP	SFN39639.1	Flavobacteria
Chryseobacterium oranimense	CYN-FKBP	WP_040999021.1	Flavobacteria
Chryseobacterium palustre	CYN-FKBP	WP_051190467.1	Flavobacteria
Chryseobacterium piperi	CYN-FKBP	WP_034687466.1	Flavobacteria
Chryseobacterium piscicola	CYN-FKBP	SIS72842.1	Flavobacteria
Chryseobacterium polytrichastri	CYN-FKBP	SHL10481.1	Flavobacteria
Chryseobacterium scophthalmum	CYN-FKBP	WP_074228863.1	Flavobacteria
Chryseobacterium shigense	CYN-FKBP	WP_076510030.1	Flavobacteria
Chryseobacterium soldanellicola	CYN-FKBP	SDR14823.1	Flavobacteria
Chryseobacterium solincola	CYN-FKBP	WP_039344647.1	Flavobacteria
Chryseobacterium sp. Hurlbut01	CYN-FKBP	KNB60550.1	Flavobacteria
Chryseobacterium taeanense	CYN-FKBP	SDH88102.1	Flavobacteria
Chryseobacterium taihuense	CYN-FKBP	SDM11487.1	Flavobacteria
Chryseobacterium taiwanense	CYN-FKBP	WP_039365462.1	Flavobacteria
Chryseobacterium tenax	CYN-FKBP	WP_034980653.1	Flavobacteria
Chryseobacterium treverense	CYN-FKBP	SFI64973.1	Flavobacteria
Chryseobacterium ureilyticum	CYN-FKBP	WP_076552336.1	Flavobacteria
Chryseobacterium wanjuense	CYN-FKBP	SEW20341.1	Flavobacteria
Cloacibacterium normanense	CYN-FKBP	WP_069798429.1	Flavobacteria
Croceibacter atlanticus	CYN-FKBP	WP_013187883.1	Flavobacteria
Crocinitomix catalasitica	CYN-FKBP	WP_084274406.1	Flavobacteria
Cruoricaptor ignavus	CYN-FKBP	SHI54158.1	Flavobacteria
Cryomorphaceae bacterium	CYN-FKBP	KRP28643.1	Flavobacteria
Desulfotalea psychrophila	CYN-FKBP	CAG36374.1	Flavobacteria
Dokdonia donghaensis (multiple strains)	CYN-FKBP	WP_021778660.1	Flavobacteria
Elizabethkingia anophelis	CYN-FKBP	OCW74537.1	Flavobacteria
Elizabethkingia meningoseptica	CYN-FKBP	EOR30682.1	Flavobacteria
Elizabethkingia miricola	CYN-FKBP	AJW61711.1	Flavobacteria
Eudoraea adriatica	CYN-FKBP	WP_019668468.1	Flavobacteria
Flagellimonas sp.	CYN-FKBP	WP_055397607.1	Flavobacteria
Flavobacterium aquidurense	CYN-FKBP	WP_055098333.1	Flavobacteria
Flavobacteria bacterium BAL38	CYN-FKBP	EAZ96145.1	Flavobacteria
Flavobacteria bacterium BBFL7	CYN-FKBP	WP_006795114.1	Flavobacteria
Flavobacteriaceae bacterium	CYN-FKBP	OIP45389.1	Flavobacteria
Flavobacteriales bacterium	CYN-FKBP	WP_013306554.1	Flavobacteria

Flavobacterium akiainvivens	CYN-FKBP	WP_054407344.1	Flavobacteria
Flavobacterium antarcticum	CYN-FKBP	WP_022827013.1	Flavobacteria
Flavobacterium beibuense	CYN-FKBP	KGO82584.1	Flavobacteria
Flavobacterium branchiophilum	CYN-FKBP	WP_014084292.1	Flavobacteria
Flavobacterium cauense	CYN-FKBP	ESU21328.1	Flavobacteria
Flavobacterium chungangense	CYN-FKBP	WP_031454950.1	Flavobacteria
Flavobacterium columnare	CYN-FKBP	AMA49476.1	Flavobacteria
Flavobacterium daejeonense	CYN-FKBP	WP_055090896.1	Flavobacteria
Flavobacterium defluvii	CYN-FKBP	SHH23133.1	Flavobacteria
Flavobacterium enshiense	CYN-FKBP	WP_023573905.1	Flavobacteria
Flavobacterium filum	CYN-FKBP	WP_051220716.1	Flavobacteria
Flavobacterium frigoris	CYN-FKBP	WP_007137991.1	Flavobacteria
Flavobacterium frigoris	CYN-FKBP	WP_007138038.1	Flavobacteria
Flavobacterium fryxellicola	CYN-FKBP	OAB27313.1	Flavobacteria
Flavobacterium gilvum	CYN-FKBP	AOW09633.1	Flavobacteria
Flavobacterium haoranii	CYN-FKBP	SHJ78198.1	Flavobacteria
Flavobacterium hibernum	CYN-FKBP	WP_041519349.1	Flavobacteria
Flavobacterium hibernum	CYN-FKBP	WP_041519676.1	Flavobacteria
Flavobacterium hydatis	CYN-FKBP	WP_035622385.1	Flavobacteria
Flavobacterium hydatis	CYN-FKBP	WP_035626885.1	Flavobacteria
Flavobacterium indicum	CYN-FKBP	WP_014388319.1	Flavobacteria
Flavobacterium johnsoniae	CYN-FKBP	AAM92026	Flavobacteria
Flavobacterium johnsoniae	CYN-FKBP	AAM92027	Flavobacteria
Flavobacterium limnosediminis	CYN-FKBP	WP_023579025.1	Flavobacteria
Flavobacterium omnivorum	CYN-FKBP	SDH25424.1	Flavobacteria
Flavobacterium phragmitis	CYN-FKBP	SFD64262.1	Flavobacteria
Flavobacterium psychrophilum	CYN-FKBP	WP_034098691.1	Flavobacteria
Flavobacterium psychrophilum JIP02/86	CYN-FKBP	YP_001296758.1	Flavobacteria
Flavobacterium psychrophilum JIP02/86	CYN-FKBP	YP_001296759.1	Flavobacteria
Flavobacterium rivuli	CYN-FKBP	WP_020211598.1	Flavobacteria
Flavobacterium reichenbachii	CYN-FKBP	WP_035684065.1	Flavobacteria
Flavobacterium saliperosum S13	CYN-FKBP	ESU26021.1	Flavobacteria
Flavobacterium seoulense	CYN-FKBP	WP_035657803.1	Flavobacteria
Flavobacterium seoulense	CYN-FKBP	WP_035658288.1	Flavobacteria
Flavobacterium sinopsychrotolerans	CYN-FKBP	SEN91439.1	Flavobacteria
Flavobacterium soli	CYN-FKBP	WP_026705641.1	Flavobacteria
Flavobacterium sp. ACAM 123	CYN-FKBP	WP_016991130.1	Flavobacteria
Flavobacterium sp. Leaf82	CYN-FKBP	KQO32644.1	Flavobacteria
Flavobacterium subsaxonicum	CYN-FKBP	WP_026991669.1	Flavobacteria
Flavobacterium succinicans	CYN-FKBP	WP_024981134.1	Flavobacteria
Flavobacterium succinicans	CYN-FKBP	WP_024981364.1	Flavobacteria
Flavobacterium swingsii	CYN-FKBP	SFA88116.1	Flavobacteria
Flavobacterium urocaniciphilum	CYN-FKBP	SEP61984.1	Flavobacteria
Flavobacterium xinjiangense	CYN-FKBP	WP_073207629.1	Flavobacteria
Fluviicola taffensis DSM 16823	CYN-FKBP	WP_013686748.1	Flavobacteria
Formosa agariphila	CYN-FKBP	CDF78794.1	Flavobacteria
Formosa haliotis	CYN-FKBP	WP_066219441.1	Flavobacteria

Formosa sp. Hel1_33_131	CYN-FKBP	AOR27263.1	Flavobacteria
Gaetbulibacter saemankumensis	CYN-FKBP	WP_027136884.1	Flavobacteria
Galbibacter marinus	CYN-FKBP	WP_008991540.1	Flavobacteria
Gelidibacter mesophilus	CYN-FKBP	WP_027126492.1	Flavobacteria
Gillisia limnaea	CYN-FKBP	WP_006989975.1	Flavobacteria
Gillisia marina	CYN-FKBP	WP_010231874.1	Flavobacteria
Gramella echinicola	CYN-FKBP	WP_026933765.1	Flavobacteria
Gramella forsetii	CYN-FKBP	CAL65762	Flavobacteria
Gramella forsetii	CYN-FKBP	WP_011710397.1	Flavobacteria
Gramella sp. LPB0144	CYN-FKBP	APG58900.1	Flavobacteria
Hyunsoonleella jejuensis	CYN-FKBP	SEQ10693.1	Flavobacteria
Imtechella halotolerans	CYN-FKBP	WP_008239677.1	Flavobacteria
Jejuia pallidilutea	CYN-FKBP	WP_045372778.1	Flavobacteria
Jejuia pallidilutea	CYN-FKBP	GAL66646.1	Flavobacteria
Kordia algicida OT-1	CYN-FKBP	WP_007093789.1	Flavobacteria
Kriegella aquimaris	CYN-FKBP	SDM97151.1	Flavobacteria
Lacinutrix jangbogonensis	CYN-FKBP	WP_034057582.1	Flavobacteria
Lacinutrix sp. 5H-3-7-4	CYN-FKBP	WP_013870480.1	Flavobacteria
Leeuwenhoekiella blandensis	CYN-FKBP	WP_009781285.1	Flavobacteria
Leeuwenhoekiella palythoae	CYN-FKBP	WP_072983594.1	Flavobacteria
Leeuwenhoekiella marinoflava	CYN-FKBP	WP_073099465.1	Flavobacteria
Lishizhenia tianjinensis	CYN-FKBP	SFT67407.1	Flavobacteria
Lutibacter maritimus	CYN-FKBP	SFS32105.1	Flavobacteria
Lutibacter profundi	CYN-FKBP	AMC10344.1	Flavobacteria
Mangrovimonas yunxiaonensis	CYN-FKBP	WP_036122544.1	Flavobacteria
Maribacter aquivivus	CYN-FKBP	SHJ89650.1	Flavobacteria
Maribacter orientalis	CYN-FKBP	SEL31201.1	Flavobacteria
Maribacter sp. HTCC2170	CYN-FKBP	WP_013306554.1	Flavobacteria
Maribacter stanieri	CYN-FKBP	SFR67453.1	Flavobacteria
Mesoflavibacter zeaxanthinifaciens	CYN-FKBP	WP_010517508.1	Flavobacteria
Muricauda antarctica	CYN-FKBP	SFB76710.1	Flavobacteria
Muricauda lutaonensis	CYN-FKBP	WP_045800951.1	Flavobacteria
Muricauda ruestringensis	CYN-FKBP	WP_014031611.1	Flavobacteria
Muricauda zhangzhouensis	CYN-FKBP	SDQ56770.1	Flavobacteria
Myroides guanonis	CYN-FKBP	SFJ87275.1	Flavobacteria
Myroides injenensis	CYN-FKBP	WP_010250164.1	Flavobacteria
Myroides marinus	CYN-FKBP	KUF40224.1	Flavobacteria
Myroides odoratimimus	CYN-FKBP	EKB03227.1	Flavobacteria
Myroides odoratus	CYN-FKBP	WP_060875118.1	Flavobacteria
Myroides phaeus	CYN-FKBP	SDH72867.1	Flavobacteria
Nonlabens dokdonensis	CYN-FKBP	AGC75181.1	Flavobacteria
Nonlabens marinus	CYN-FKBP	WP_041496246.1	Flavobacteria
Nonlabens sedimins	CYN-FKBP	WP_042280300.1	Flavobacteria
Nonlabens ulvanivorans	CYN-FKBP	GAL75631.1	Flavobacteria
Ochrovirga pacifica	CYN-FKBP	WP_010134755.1	Flavobacteria
Olleya marilimosa	CYN-FKBP	WP_028282856.1	Flavobacteria
Owenweeksia hongkongensis	CYN-FKBP	WP_014201615.1	Flavobacteria

Polaribacter atrinae	CYN-FKBP	WP_068449940.1	Flavobacteria
Polaribacter dokdonensis	CYN-FKBP	WP_053975013.1	Flavobacteria
Polaribacter irgensii	CYN-FKBP	WP_004570747.1	Flavobacteria
Polaribacter reichenbachii	CYN-FKBP	WP_068364573.1	Flavobacteria
Polaribacter sp. MED152	CYN-FKBP	WP_015480498.1	Flavobacteria
Polaribacter sp. Hel1_85	CYN-FKBP	WP_036821955.1	Flavobacteria
Polaribacter vadi	CYN-FKBP	WP_065319140.1	Flavobacteria
Pricia antarctica	CYN-FKBP	SDE04035.1	Flavobacteria
Psychroflexus gondwanensis	CYN-FKBP	WP_003436956.1	Flavobacteria
Psychroflexus sediminis	CYN-FKBP	SDG55100.1	Flavobacteria
Psychroflexus torquis	CYN-FKBP	AFU67563.1	Flavobacteria
Psychroflexus tropicus	CYN-FKBP	WP_019038212.1	Flavobacteria
Psychroserpens burtonensis	CYN-FKBP	WP_028873302.1	Flavobacteria
Psychroserpens damuponensis	CYN-FKBP	WP_040278254.1	Flavobacteria
Riemerella anatipestifer DSM 15868	CYN-FKBP	WP_013446672.1	Flavobacteria
Riemerella anatipestifer RA-YM	CYN-FKBP	EFT35731.1	Flavobacteria
Riemerella columbina	CYN-FKBP	WP_018676593.1	Flavobacteria
Riemerella columbipharyngis	CYN-FKBP	SDE47300.1	Flavobacteria
Robiginitalea biformata	CYN-FKBP	WP_015754216.1	Flavobacteria
Robiginitalea biformata HTCC2501	CYN-FKBP	WP_015755540.1	Flavobacteria
Runella limosa	CYN-FKBP	WP_028522983.1	Flavobacteria
Salegentibacter mishustinae	CYN-FKBP	WP_057481232.1	Flavobacteria
Salegentibacter salegens	CYN-FKBP	WP_079734691.1	Flavobacteria
Salegentibacter salinarum	CYN-FKBP	WP_079713874.1	Flavobacteria
Salinimicrobium catena	CYN-FKBP	SDL76680.1	Flavobacteria
Sediminibacter sp. Hel_I_10	CYN-FKBP	WP_026752871.1	Flavobacteria
Sediminicola sp	CYN-FKBP	WP_062055259.1	Flavobacteria
Soonwooa buanensis	CYN-FKBP	WP_079665888.1	Flavobacteria
Tamlana nanhaiensis	CYN-FKBP	WP_044625819.1	Flavobacteria
Tenacibaculum dicentrarchi	CYN-FKBP	WP_058884629.1	Flavobacteria
Tenacibaculum maritimum	CYN-FKBP	WP_024741596.1	Flavobacteria
Tenacibaculum ovolyticum	CYN-FKBP	WP_028889491.1	Flavobacteria
Tenacibaculum sp.	CYN-FKBP	WP_028891023.1	Flavobacteria
Ulvibacter litoralis	CYN-FKBP	SDE92800.1	Flavobacteria
Vitellibacter aquimaris	CYN-FKBP	WP_062622682.1	Flavobacteria
Wenyingzhuangia fucanilytica	CYN-FKBP	WP_068826390.1	Flavobacteria
Weeksella virosa DSM 16922	CYN-FKBP	WP_013598693.1	Flavobacteria
Winogradskyella psychrotolerans	CYN-FKBP	WP_020896060.1	Flavobacteria
Winogradskyella sp	CYN-FKBP	WP_045468090.1	Flavobacteria
Xanthomarina gelatinilytica	CYN-FKBP	WP_007648709.1	Flavobacteria
Zhouia amylolytica	CYN-FKBP	WP_038265805.1	Flavobacteria
Zobellia galactanivorans	CYN-FKBP	WP_013994175.1	Flavobacteria
Zobellia galactanivorans	CYN-FKBP	WP_013995030.1	Flavobacteria
Zobellia uliginosa	CYN-FKBP	WP_038238738.1	Flavobacteria
Zunongwangia mangrovi	CYN-FKBP	SFC62278.1	Flavobacteria
Zunongwangia profunda SM-A87	CYN-FKBP	WP_013071163.1	Flavobacteria
Zunongwangia profunda SM-A87	CYN-FKBP	WP_013070348.1	Flavobacteria

Others

Ancylostoma ceylanicum (Nematode)	CYN-FKBP	EPB65547.1	Nematode
Candidatus Ruthia magnifica	CYN-FKBP	WP_011737713.1	γ-proteobacteria
Candidatus Thioglobus	CYN-FKBP	WP_053820422.1	γ-proteobacteria
Chitinivibrio alkaliphilus ACht1	CYN-FKBP	ERP38862.1	Fibrobacteres
Desulfobacterales bacterium	CYN-FKBP	OGR19343.1	δ-Proteobacteria
Desulfocapsa sulfexigens	CYN-FKBP	AGF76656.1	δ-Proteobacteria
Desulfobulbus propionicus DSM 2032	CYN-FKBP	WP_015723644.1	δ-Proteobacteria
Desulfotalea psychrophila	CYN-FKBP	WP_011188886.1	δ-Proteobacteria
Hyalella azteca	CYN-FKBP	XP_018019590.1	Arthropod
Nitrospira bacterium	CYN-FKBP	KPK34464.1	Nitrospira
