# Supplementary material for: Bioinformatic Analysis Reveals Conservation of Intrinsic Disorder in the Linker Sequences of Prokaryotic Dual-family Immunophilin Chaperones
Source: Comput Struct Biotechnol J. 2017 Dec 30;16:6–14. doi: 10.1016/j.csbj.2017.12.002 (PMC5852385; doi:10.1016/j.csbj.2017.12.002)
Supplement: Supplementary Material 3 — Secondary structure map of Treponema denticola CFBP, based on homology modeling against bovine CyP40 (PDB 1iip) [1] and Arabidopsis thaliana FKBP42 (2if4) [2]. [file mmc3.docx]

**Supplementary Material 3**

Secondary structure map of *Treponema denticola* CFBP, based on homology modeling against bovine CyP40 (PDB 1iip) [1] and *Arabidopsis thaliana* FKBP42 (2if4) [2].

MKKLWIMIIAIAFMILVAGTAAAIIITNSGSEKGDKNMNNLKNIEALKEDGLYAAIDTDKGLIVLKLFYKETPLTVCNFVGLAEGTLDAAKGKPFYDGLTFHRVIADFMIQGGDPTGTGSGGPGYRFPDEIVEDLKHDGPGVLSMANAGPGTNGSQFFITHVETPWLDGKHTIFGRVVEGQNVVDSIQQGNKIKT**VKIIRTGNEAKAFKTDQEAFYKYLAETKESEKRRAEAFAKKMEDLIKTKYSP**AKLDDDGVYSFVVKQGKGDTPKQGQTLTMKYKGSLLENGKVFDDSDMHKPLEFPVGLGRVIPGFDSQSAKMTLGEKRIIIIPPHLAYGEAGAGGVIPPNAYLVFELELLNIK

(Color Key: Red = α-helix; Green = β-strand; Blue = Coiled-coil).

Alignment by BLAST

CYN alignment:

Bovine 30 GRIVLELFADIVPKTAENFRALCTGEKGIGPTTGKPLHFKGCPFHRIIKKFMIQGGDFSN 89

G IVL+LF P T NF L G + GKP + G FHR+I FMIQGGD +

T. den 61 GLIVLKLFYKETPLTVCNFVGLAEGT--LDAAKGKPF-YDGLTFHRVIADFMIQGGDPTG 117

Bovine 90 QNGTGGESIYGEKFEDENFH-YKHDKEGLLSMANAGSNTNGSQFFITTVPTPHLDGKHVV 148

G +F DE KHD G+LSMANAG TNGSQFFIT V TP LDGKH +

T. den 118 TG----SGGPGYRFPDEIVEDLKHDGPGVLSMANAGPGTNGSQFFITHVETPWLDGKHTI 173

Bovine 149 FGQVIKGMGVAKILENVEV

FG+V++G V ++

T. den 174 FGRVVEGQNVVDSIQ----

FKBP alignment:

A. tha 42 SEAEVLDEKVSKQIIKEGHGSKPSKYSTCFLHYRAWTKNSQHKFEDTWHEQQPIELVLGK 101

S A++ D+ V ++K+G G P + T + Y+ + F+D+ +P+E +G

T. den 246 SPAKLDDDGVYSFVVKQGKGDTPKQGQTLTMKYKGSLLENGKVFDDS-DMHKPLEFPVGL 304

A. tha 102 EKKELAGLAIGVASMKSGERALVHVGWELAYGKEGNFSFPNVPPMADLLYEVEVI 156

+ + G A M GE+ ++ + LAYG+ G +PP A L++E+E++

T. den 305 -GRVIPGFDSQSAKMTLGEKRIIIIPPHLAYGEAGAGGV--IPPNAYLVFELELL 356

**References:**

[1] Taylor P, Dornan J, Carrello A, Minchin RF, Ratajczak T, Walkinshaw MD (2001) Two structures of cyclophilin 40: folding and fidelity in the TPR domains. Structure 9:431-438

[2] Granzin J, Eckhoff A, Weiergraber OH (2006) Crystal structure of a multi-domain immunophilin from *Arabidopsis thaliana*: a paradigm for regulation of plant ABC transporters. J Mol Biol 364:799-809
